# Ratiometric quantitation of redox status with a molecular Fe_2_ magnetic resonance probe†
†Electronic supplementary information (ESI) available: Experimental details and additional physical data for **1**, **2**, **3**, and crystallographic information file (CIF) for LFe_2_(etidronate)·7H_2_O. CCDC 1531010. For ESI and crystallographic data in CIF or other electronic format see DOI: 10.1039/c7sc00562h



**DOI:** 10.1039/c7sc00562h

**Published:** 2017-04-19

**Authors:** Kang Du, Emily A. Waters, T. David Harris

**Affiliations:** a Department of Chemistry , Northwestern University , 2145 Sheridan Road , Evanston , IL 60208-3113 , USA . Email: dharris@northwestern.edu; b Center for Advanced Molecular Imaging , Northwestern University , 2145 Sheridan Road , Evanston , IL 60208-3113 , USA

## Abstract

We demonstrate the ability of a molecular Fe_2_ complex to enable magnetic resonance (MR)-based ratiometric quantitation of redox status, namely through redox-dependent paramagnetic chemical exchange saturation transfer (PARACEST). Metalation of a tetra(carboxamide) ligand with Fe^II^ and/or Fe^III^ in the presence of etidronate ion affords analogous FeII2, Fe^II^Fe^III^, and FeIII2 complexes. Both FeII2 and Fe^II^Fe^III^ complexes give highly-shifted, sharp, and non-overlapping NMR spectra, with multiple resonances for each complex corresponding to exchangeable carboxamide protons. These protons can be selectively irradiated to give CEST peaks at 74 and 83 ppm *vs.* H_2_O for the Fe^II^Fe^III^ complex and at 29, 40 and 68 ppm for the FeII2 complex. The CEST spectra obtained from a series of samples containing mixtures of FeII2 and Fe^II^Fe^III^ are correlated with independently-determined open-circuit potentials to construct a Nernstian calibration curve of potential *vs.* CEST peak intensity ratio. In addition, averaged intensities of phantom images collected on a 9.4 T MRI scanner show analogous Nernstian behavior. Finally, both the FeII2 and Fe^II^Fe^III^ forms of the complex are stable to millimolar concentrations of H_2_PO_4_^–^/HPO_4_^2–^, CO_3_^2–^, SO_4_^2–^, CH_3_COO^–^, and Ca^2+^ ions, and the FeIII2 form is air-stable in aqueous buffer and shows >80% viability in melanoma cells at millimolar concentration. The stability suggests the possible application of this or related complexes for *in vivo* studies. To our knowledge, this concentration-independent method based on a single Fe_2_ probe provides the first example of MR-based ratiometric quantitation of redox environment.

## Abstract 2

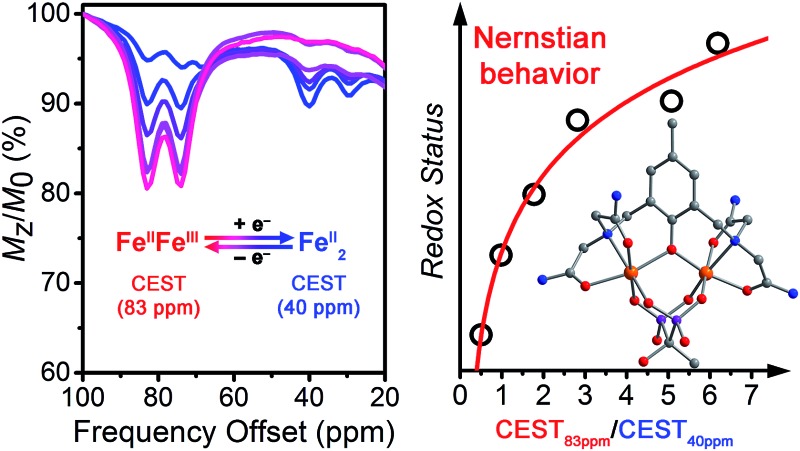
We demonstrate the ability of a molecular Fe_2_ complex to enable magnetic resonance-based ratiometric quantitation of redox status.

## Introduction

The redox status of intra- and extracellular environments is a vital biomarker for disease, as it provides a collective picture of the concentration of redox-active species, such as thiols, reactive oxygen or nitrogen species, signaling molecules (*e.g.* NO/H_2_O_2_), and redox-active proteins (*e.g.* superoxide dismutases), which are key participants in cell apoptosis and proliferation.^1^ For this reason, the ability to quantitatively interrogate redox environment represents an important challenge. One approach toward this end involves the employment of analytical methods, such as HPLC^2^ and fluorescence spectrometry,^3^ to determine the ratio of oxidized to reduced species in an extracted sample. Whereas these procedures provide important information regarding the redox-dependence of cancer-related cell activities in tissues^4^ and cultured cells,^5^ they nonetheless suffer from key disadvantages in that they require physical withdrawal of a sample and provide only a single spatiotemporal point, rather than global, measurement. Indeed, a non-invasive imaging method for spatiotemporal redox mapping would represent an invaluable tool for both diagnostic and pathological investigations of redox status.

Magnetic resonance imaging (MRI) is a powerful and non-invasive imaging technique, as it utilizes non-ionizing radio radiation that deeply penetrates tissue to provide high spatiotemporal image resolution.^6^ As such, MRI represents an ideal modality for redox mapping of tissue. Toward this end, numerous metallic molecular MRI probes have shown the capability to detect pO_2_/hypoxia,^7^ peroxide,^8^ thiols,^9^ NADH,^10^ and redox-active metals.^11^ In general, these probes show negligible contrast in one oxidation state but are activated upon oxidation or reduction to generate MRI contrast, with a number of activation mechanisms having been reported. For instance, lanthanide probes can feature redox-active pendent groups on the ligand that cause structural changes upon redox chemistry to turn contrast on or off.^9a,10^ In addition, transition metal probes can exploit metal-based redox processes, where a change in oxidation state of the metal center turns contrast on or off.^7c,e^

A key limitation of turn-on or turn-off probes is their inability to provide quantitative information about redox environment, owing to unpredictable, inhomogeneous probe concentration in tissue that results from variable biodistribution patterns. As an alternative, one can envision use of a single molecule that features two individually addressable “on” oxidation states, with the ratio of the two signals giving a concentration-independent measureable that can be used to quantitate solution redox environment. Toward this end, PARACEST represents a promising method for ratiometric redox quantitation.^12^ This technique employs paramagnetic probes with highly-shifted exchangeable protons that, upon selective irradiation, are delivered to bulk H_2_O to generate contrast. In principle, for a probe accessible in two CEST-active oxidation states, the ratio between the CEST effects of the redox states would provide a concentration-independent measure of solution redox environment.

**Fig. 1:**
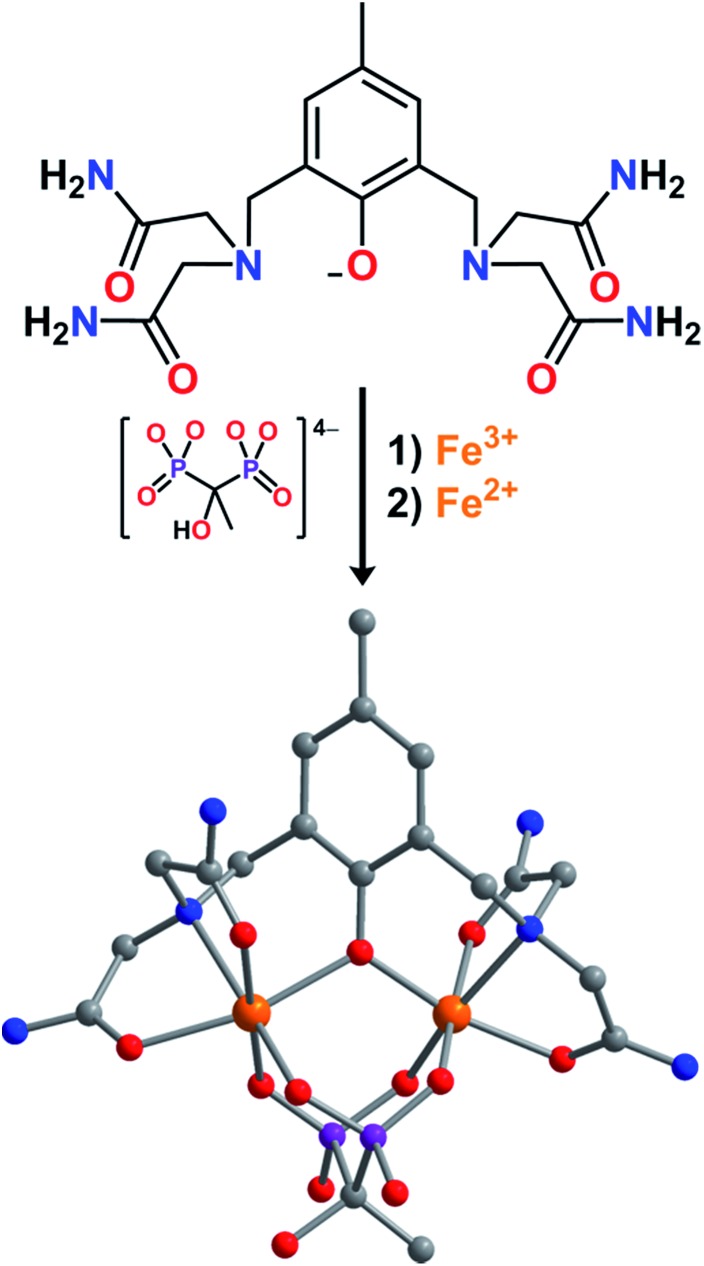
Reaction of L^–^, etidronate tetraanion, Fe^3+^, and Fe^2+^ to form LFe_2_(etidronate), as observed in LFe_2_(etidronate)·7H_2_O. Orange, purple, blue, red, and gray spheres represent Fe, P, N, O, and C atoms, respectively; H atoms are omitted for clarity.

In order for a molecule to display the PARACEST effect in two oxidation states, the metal center must first be paramagnetic in both states. One such scenario is high-spin Fe^II^ and high-spin Fe^III^. However, the electronic relaxation time (*τ*_s_) of high-spin Fe^III^ is usually too long (*ca.* 10^–10^ s) to give sharp ^1^H NMR spectra,^13^ another requirement for PARACEST. Alternatively, moving to a redox-active molecule with multiple metal centers offers a more straightforward and general strategy toward realizing multiple oxidation states with short *τ*_s_. Recently, we reported a dinucleating, tetra(carboxamide) ligand (HL; see Fig. 1) that gives rise to a Cu_2_ PARACEST agent.^14^ Herein, we report the Fe_2_ analogue and demonstrate PARACEST activity in both the Fe^II^Fe^III^ and Fe^II^Fe^II^ form that enables ratiometric quantitation of solution open-circuit potential. To our knowledge, this study provides the first example of ratiometric quantitation of solution redox environment using an MR probe.

## Results and discussion

The synthesis of the anionic complex [LFe_2_(etidronate)]^–^ was carried out through successive additions of two equivalents of [Fe(H_2_O)_6_]^2+^ and one equivalent of etidronic acid, in the presence of NMe_4_OH, to a solution of HL in methanol, to give the compound (NMe_4_)[LFeII2(etidronate)]·2.7H_2_O·THF (**1**) as a light yellow solid. The analogous mixed-valence Fe^II^Fe^III^ and univalence FeIII2 complexes were prepared similarly, but with 1 : 1 [Fe(H_2_O)_6_]^2+^ : [Fe(H_2_O)_6_]^3+^ and exclusively [Fe(H_2_O)_6_]^3+^, respectively, to afford the red compounds LFe_2_(etidronate)·0.7H_2_O·0.2THF (**2**) and [LFe_2_(etidronate)](NO_3_)·0.9H_2_O·1.5THF (**3**). The ancillary ligand etidronate improves the aqueous solubility of the neutral molecule LFe_2_(etidronate) relative to pyrophosphate, which was employed in the Cu_2_ analogue.^14^

Slow diffusion of THF vapor into a concentrated solution of **2** in H_2_O afforded plate-shaped single crystals of LFe_2_(etidronate)·H_2_O that were suitable for X-ray structural analysis. The structure features two distinct Fe centers, each in a distorted octahedral coordination environment comprising two carboxamide O atoms, one μ-phenoxo O atom, and one N atom from L^–^, along with two O atoms from a μ^2^–κ^4^ etidronate bridging ligand (see Fig. 1). The mean Fe–O bond distances for the two Fe centers are distinct at 1.992(5) and 2.125(6) Å, indicative of valence-localized high-spin Fe^III^ and Fe^II^, respectively. The Fe–O_L_–Fe angle of 118.6(3)° and Fe···Fe distance of 3.547(2) Å are consistent with related mixed-valence Fe_2_ complexes.^15^

**Fig. 2:**
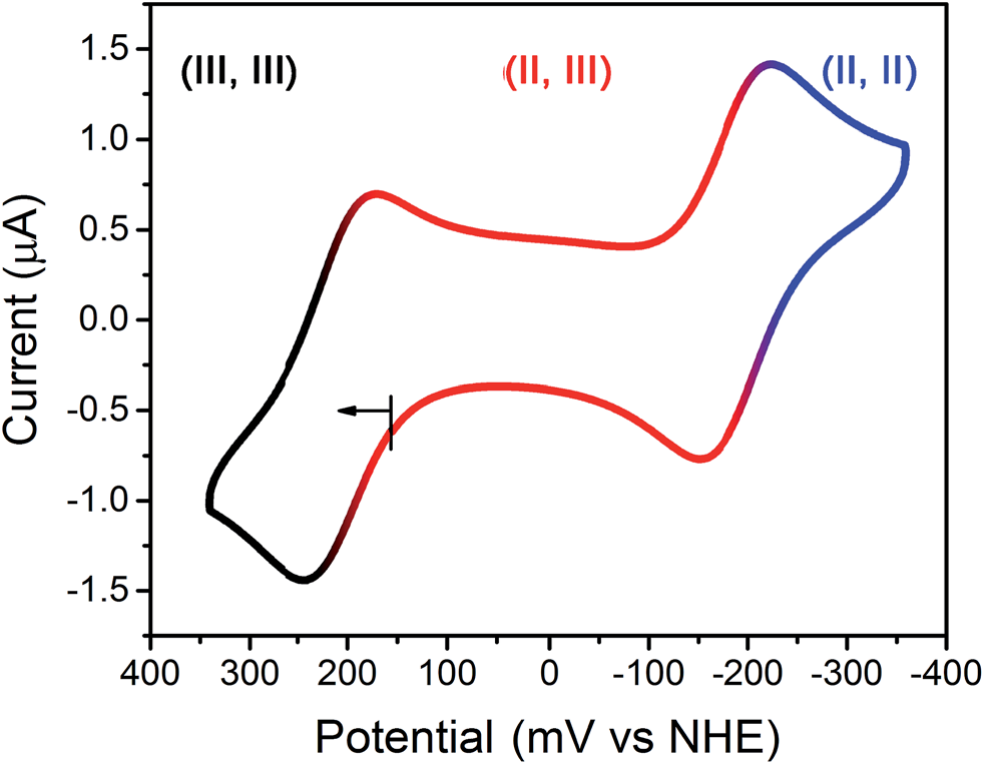
Cyclic voltammogram of **2** in solution containing 100 mM NaCl, 100 mM HEPES buffered at pH 7.4; scan rate = 50 mV s^–1^. Roman numerals represent Fe oxidation states.

To probe the redox chemistry of the Fe_2_ complex, a cyclic voltammogram was collected for an aqueous solution of **2** in HEPES buffer at pH 7.4. The voltammogram features two reversible processes at potentials of *E*_1/2_ = –187 and 209 mV *vs.* the Normal Hydrogen Electrode (NHE) (see Fig. 2). These processes are assigned to the Fe^II^Fe^II^/Fe^II^Fe^III^ and Fe^II^Fe^III^/Fe^III^Fe^III^ couples, respectively. The potential separation of Δ*E*_1/2_ = 396 mV corresponds to a comproportionation constant of *K*_c_ = 5.00 × 10^6^ for the reaction [LFe_2_(etidronate)]^+^ + [LFe_2_(etidronate)]^–^ → 2LFe_2_(etidronate), indicating that the mixed-valence complex is stable towards disproportionation. Similarly, a solution of **1** gave an identical voltammogram, albeit with a different open-circuit potential (see Fig. S1†). Importantly, the redox window observed here is wide and biologically relevant, consistent with the electrochemical potentials of intra- and extracellular environments, spanning from –300 to 0 mV *vs.* NHE.^12*g*^ Further, note that the broad library of bisphosphonate ligands offers the possibility to tune the potential window to target specific redox environments.

Mössbauer spectra were collected to investigate the nature of mixed-valency in **2**. At 80 K, the compound displays two quadrupole doublets with isomer shifts of *δ* = 1.23(1) and 0.480(5) mm s^–1^ and quadrupole splittings of Δ*E*_Q_ = 2.65(2) and 0.485(8) mm s^–1^, that we respectively assign to high-spin Fe^II^ and high-spin Fe^III^ (see Fig. S2†).^16^ The areal ratio between the two spectral components of 1 : 1.1(1) indicates a valence-trapped Fe^II^Fe^III^ electronic structure, in agreement with crystallographic analysis.^17^ In contrast, the spectrum for **1** at 80 K is best modeled with two doublets (*δ* = 1.325(8) and 1.158(7) mm s^–1^, Δ*E*_Q_ = 2.871(7) and 2.874(6) mm s^–1^) that correspond to two similar but inequivalent high-spin Fe^II^ centers (see Fig. S3†).

A solution of **2** in neutral D_2_O exhibits features at 21,277 cm^–1^ (*ε* = 861 M^–1^ cm^–1^) and 7318 cm^–1^ (*ε* = 83 M^–1^ cm^–1^) (see Fig. S4†), which we assign to ligand-to-metal charge-transfer (LMCT) and intervalence charge transfer (IVCT) bands, respectively, in accord with similar mixed-valence Fe_2_ complexes.^18^ Polar solvents often forestall electron transfer, and as such, the observation of IVCT in D_2_O is notable and highlights the stability and rigidity of the Fe_2_ complex. The IVCT full-width at half-maximum of Δ*ν*_1/2_ = 3043 cm^–1^ is lower than the theoretical linewidth of Δ*ν*01/2 = (2310(*ν*_max_))^1/2^ = 4112 cm^–1^, suggesting some degree of electron detrapping in **2**.^19^ Moreover, a linewidth analysis using the crystallographic Fe···Fe distance provides an estimate of the 298 K electron transfer rate as 6.7(1) × 10^10^ s^–1^ (see ESI† for details).^16a,20^ The solution spectrum closely resembles that of the solid-state diffuse reflectance (see Fig. S5†), suggesting that the crystallographic structure is retained in solution. In contrast to **2**, only a shoulder at 22,000 cm^–1^ (*ε* = 48 M^–1^ cm^–1^) is present in the spectrum for **1** (see Fig. S4†). This observation is consistent with a univalence FeII2 configuration and agrees with literature examples.^21^

To assess magnetic interactions in the Fe_2_ complexes, variable-temperature magnetic susceptibility measurements were carried out for solid-state samples of **1** and **2** (see Fig. S6†). At 300 K, *χ*_M_ *T* = 7.17 cm^3^ K mol^–1^ for **1**, consistent with two non-interacting high-spin Fe^II^ centers. For **2**, *χ*_M_ *T* = 6.87 cm^3^ K mol^–1^ at 300 K, consistent with high-spin Fe^III^ and Fe^II^. For both compounds, *χ*_M_ *T* decreases with decreasing temperature, albeit more rapidly for **2**, indicative of antiferromagnetic superexchange. These interactions were modeled with the spin Hamiltonian *Ĥ* = –2*J*(*ŝ*_Fe1_·*ŝ*_Fe2_),^22^ to give exchange coupling constants *J* = –0.8(3) cm^–1^ for **1** and *J* = –3.6(5) cm^–1^ for **2**. The stronger coupling in **2** is likely due to the shorter Fe^III^–O bond relative to the Fe^II^–O bond in **1**. These values are comparable to those previously reported for structurally-similar Fe_2_ complexes.^15^ Solution magnetic moments for **1**, **2** and **3** were measured as *χ*_M_ *T* = 7.3(3), 7.0(6) and 8.9(3) cm^3^ K mol^–1^ at 310 K, respectively, consistent with the solid-state magnetic data and the presence of exclusively high-spin Fe^II^ and Fe^III^. The larger than expected *χ*_M_ *T* for **1** is likely due to the anisotropic Fe^II^, as evidenced by the *g* value (see magnetic experimental section in ESI†).

Neutral aqueous solutions of **1** and **2** gave sharp, well-resolved ^1^H NMR spectra. The spectrum for **1** features 20 paramagnetically shifted resonances that range from –50 to 150 ppm (see Fig. S7†), with exchangeable protons appearing at –9.5, 8.5, 29, 40 and 68 ppm established by comparing the spectra obtained in D_2_O and H_2_O. These five resonances are assigned to four structurally inequivalent carboxamide groups and the etidronate hydroxyl group. In comparison, 14 paramagnetically shifted resonances are present in the spectrum for **2**, ranging from –10 to 320 ppm (see Fig. S8†). The resonances at 74 and 83 ppm are assigned to exchangeable protons on the carboxamide groups, as evidenced by their disappearance in the presence of D_2_O. The full width at half maximum for the resonances of **1** and **2** are 65–820 Hz and 44–620 Hz, respectively. The similarity in linewidth suggests that the smaller number of observed paramagnetic resonances in **2** relative to **1** likely arises from peak-averaging caused by a fast electron transfer rate of 6.7(1) × 10^10^ s^–1^, rather than from peak-broadening caused by nuclear relaxation. Furthermore, spin-lattice relaxation times (*T*_1_) for H_2_O, in samples containing 4.9 mM of **1** or **2** buffered at pH 7.4, are 1.30(1) and 1.14(1) s, respectively. The similar resonance linewidth and *T*_1_ profiles suggest a shortening of *τ*_s_ in Fe^III^, which otherwise would have imposed significant nuclear relaxation and thus severe line broadening. Such shortening of *τ*_s_ is likely due to the magnetic coupling to a fast-relaxing Fe^II^,^12^ as well as fast electron-transfer between the two Fe centers. In sum, the significantly different but sharp carboxamide resonances in **1** and **2** suggests the possibility to observe the CEST effect for the Fe_2_ probe in both oxidation states.

To investigate the possibility of CEST, ^1^H NMR spectra were collected for aqueous 3.4 mM solutions of **1** or **2** with 100 mM HEPES and 100 mM NaCl buffered at pH 7.4, using presaturation at frequencies ranging from –100 to 100 ppm referenced to H_2_O. The CEST spectrum, or *Z*-spectrum, shows the extent of H_2_O signal intensity reduction with respect to the saturation frequency, or frequency offset. In the spectrum for **1**, three CEST peaks appear at 29, 40 and 68 ppm with 8.8, 10 and <5% H_2_O signal reduction, respectively (see Fig. S9, top†). Note that any CEST effect stemming from the two upfield labile protons at –9.5 and 8.5 ppm are likely masked by direct saturation of H_2_O. In comparison, two CEST peaks are present in the CEST spectrum for **2**, centered at 74 and 83 ppm with 21 and 22% H_2_O signal reduction, respectively (see Fig. S9, bottom†). Using the omega plot method,^23^ the proton exchange rates were estimated as 6.5(8) × 10^2^ (29 ppm) and 5.0(8) × 10^2^ (40 ppm) s^–1^ for **1**, and 6.8(9) × 10^2^ (74 ppm) and 7.0(8) × 10^2^ (83 ppm) s^–1^ for **2**, respectively (see Fig. S10 and S11†), in agreement with rates reported in mononuclear Fe^II^ carboxamide PARACEST agents.^24^ Most importantly, the orthogonality of CEST peaks for **1** and **2** suggests the possibility for ratiometric measurements.

**Fig. 3:**
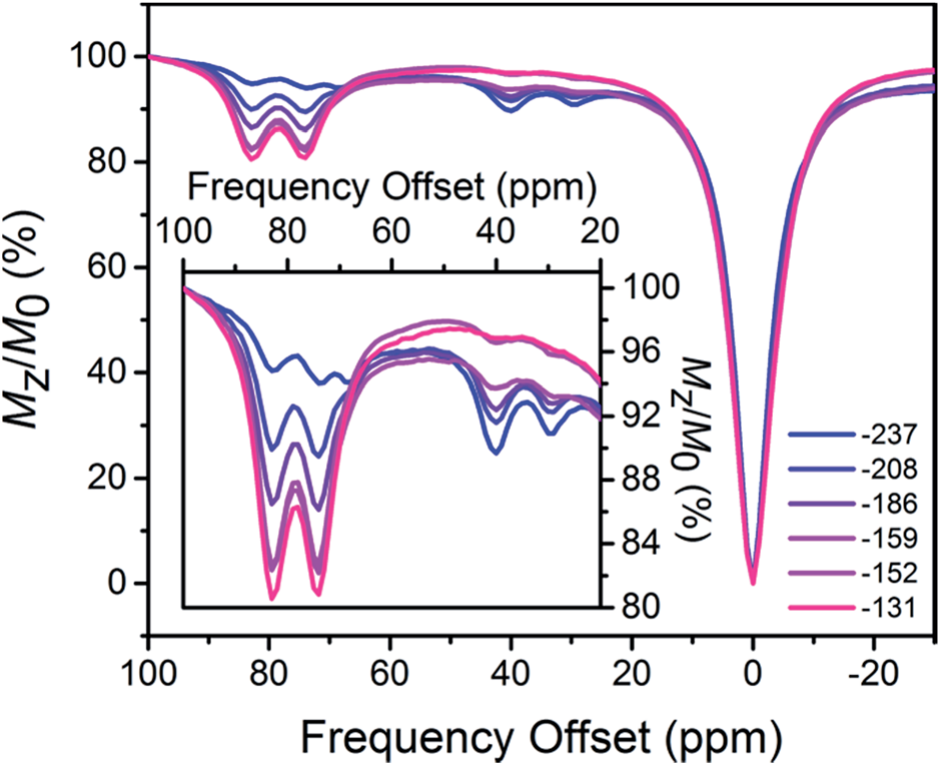
CEST spectra collected at 37 °C for 4.9 mM aqueous solutions of **1** and **2** at pH 7.4, with ratios of **1** : **2** from 9 : 1 (blue) to 1 : 9 (red). The legend gives the independently obtained OCP of each sample (mV *vs.* NHE). Inset: expanded view of the relevant CEST peaks.

**Fig. 4:**
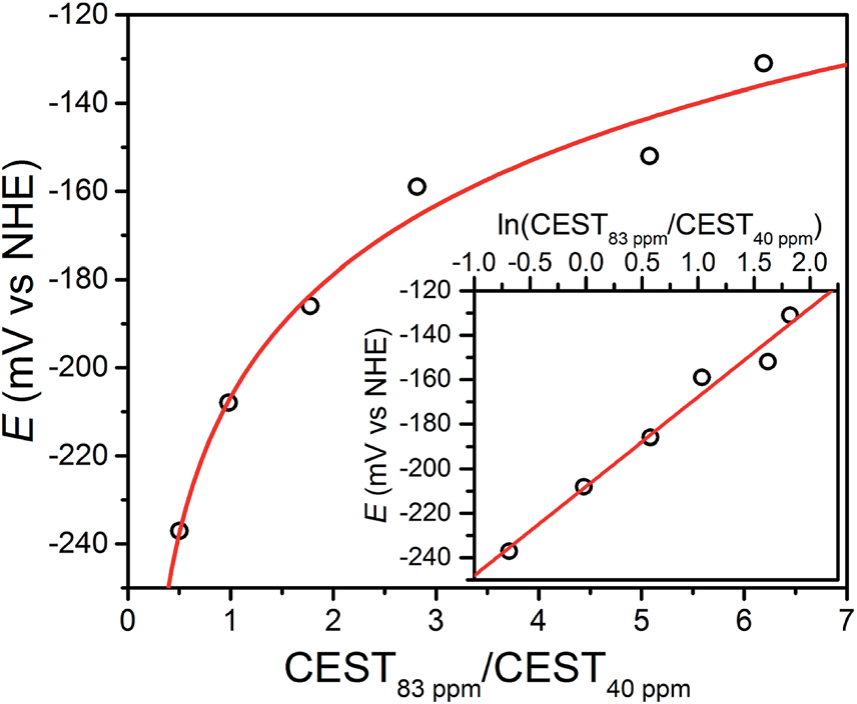
Open-circuit potential (OCP) of aqueous Fe_2_ samples *vs.* ratios of CEST peak intensities from presaturation at 83 and 40 ppm. Inset: semilog form of the plot. Black circles represent experimental data; red lines represent fits.

The open-circuit potential (OCP) of an electrochemical cell provides an experimental measure of the reducing or oxidizing nature of the solution environment. For a system at equilibrium, the OCP represents a collective measure of the ratio between the oxidized and reduced forms of each redox-active species and follows the Nernst equation. Therefore, we constructed a ratiometric calibration curve over a range of OCPs centered around the Fe^II^Fe^II^/Fe^II^Fe^III^ redox couple. Specifically, we collected CEST spectra for a series of solutions containing 100 mM HEPES, 100 mM NaCl, and selected ratios of **1** : **2** (total [Fe_2_] = 4.9 mM) in the range 9 : 1 to 1 : 9, and then correlated these spectral ratios to OCPs obtained independently using a potentiostat (see Fig. 3). Importantly, note that the OCP values stabilized within a variation of <1 mV after seconds, suggesting relatively fast kinetics toward reaching equilibrium (see Fig. S12–S15†). As the OCP becomes more reducing (*i.e.* a higher fraction of **1**), the CEST intensity at 74 and 83 ppm monotonically decreases with a concomitant increase in intensity at 29 and 40 ppm. To construct a calibration curve, the ratio of % CEST effect at 83 and 40 ppm (CEST_83 ppm_/CEST_40 ppm_) was plotted as a function of OCP (see Fig. 4). The corresponding data follow Nernstian behavior, and can therefore be fit to the following equation where OCP varies linearly with the semilog of CEST_83 ppm_/CEST_40 ppm_:

1 OCP (mV) = 40.1 ln(CEST_83 ppm_/CEST_40 ppm_) – 208

The effectiveness of eqn (1) to quantitate OCP in the presence of potassium superoxide and cysteine was examined. First, a solution containing 4 mM of **1** was incubated with 1 mM of KO_2_ in pH 7.4 buffer. The resulting CEST spectrum exhibits peaks at 74 and 83 ppm, in addition to those from **1**, arising from the oxidation product LFe_2_(etidronate) (see Fig. S16†). The % CEST at 83 and 40 ppm is 7.1 and 5.3, respectively, resulting in a calculated OCP of –219 mV based on eqn (1), which is in excellent agreement with the experimentally determined OCP of –225 mV. In another sample, 4 mM of **2** was incubated with 200 mM of cysteine in pH 7.4 buffer. The calculated OCP based on % CEST at 83 and 40 ppm and eqn (1) was –187 mV, which is in good agreement with the experimental OCP of –204 mV (see Fig. S17†). Both reactions exhibit reasonably fast kinetics, with the KO_2_ oxidation reaching equilibrium in 10 minutes and the cysteine reduction in 50 minutes, as evidenced by the temporal stabilization of OCP (see Fig. S18†). These experiments demonstrate the responsiveness of the Fe_2_ probe towards thiol and superoxide, as well as the ability of eqn (1) to quantitate solution OCP dictated by thiol-based reductants and reactive oxygen species-based oxidants.

We next sought to determine how factors such as pH and temperature affect this calibration curve, as these factors exhibit slight heterogeneity in physiological conditions. Most notably, pH affects the exchange rate of CEST-active protons, which leads to changes in CEST peak intensities. However, such changes can be partially compensated by taking the ratio of two CEST peaks in the event that both are altered to similar degree. To investigate pH effects, two series of solutions, buffered at pH 7.3 and 7.5, respectively, were prepared analogously to those at pH 7.4. Fits of the obtained OCPs as a function of CEST_83 ppm_/CEST_40 ppm_ for the two series gave the following two Nernstian equations (see Fig. S19–S22†):

2 pH 7.3: OCP (mV) = 36.4 ln(CEST_83 ppm_/CEST_40 ppm_) – 218

3 pH 7.5: OCP (mV) = 41.5 ln(CEST_83 ppm_/CEST_40 ppm_) – 216

Eqn (1)–(3) are summarized in Fig. S23.† For a given ln(CEST_83 ppm_/CEST_40 ppm_) value, the maximum deviation in OCP over the entire range of potentials was found to be *ca.* 20 mV. This value represents the maximum expected error introduced into the calibration curve by pH inhomogeneity of 7.3–7.5.

In addition to pH, temperature can also alter the intensity and frequency offset of the CEST peak, owing to increased proton exchange rate and the temperature dependence of hyperfine shift.^12^ To investigate effects from temperature variation, the data collected at pH 7.5, which feature slightly more significant CEST effects due to base-catalyzed proton exchange mechanism, were examined at 35 and 39 °C, respectively. Note that the CEST peak at 83 ppm at 37 °C shifted to 84 and 82 ppm at 35 and 39 °C, respectively, while the variable-temperature shift in the CEST peak at 40 ppm was insignificant (see Fig. S24–S27†). Fits of the OCP *vs.* CEST_83 ppm_/CEST_40 ppm_ plots for data obtained at 35 and 39 °C gave the following two Nernstian equations (see Fig. S24–S27†):

4 35 °C: OCP (mV) = 59.9 ln(CEST_83 ppm_/CEST_40 ppm_) – 246

5 39 °C: OCP (mV) = 48.6 ln(CEST_83 ppm_/CEST_40 ppm_) – 211

Using the same analysis used in the pH series, for a given ln(CEST_83 ppm_/CEST_40 ppm_) value, the largest deviation in the OCP readout was found to be *ca.* 40 mV (see Fig. S28†).

The kinetic and thermodynamic properties of **1** and **2** towards ions, air and reductants were examined by comparison of electronic absorption and NMR spectra. In the presence of 4 mM solutions of the ions H_2_PO_4_^–^/HPO_4_^2–^, CO_3_^2–^, SO_4_^2–^, CH_3_COO^–^, or Ca^2+^, incubated at 37 °C for 12 h, 4 mM of **1** or **2** in solutions buffered at pH 7.4 or D_2_O show identical NMR spectra to solutions containing the respective Fe_2_ complex with no added ions (see Fig. S29–S36†). The experiments demonstrate the high stability of the Fe_2_ complexes towards physiological ions of millimolar concentrations.^25^ Finally, the observation of CEST arising from **1** and **2** was confirmed in bovine blood plasma (see Fig. S37†). While the baseline is broader than in the spectra obtained in buffer solution, presumably due to the presence of additional exchangeable protons from proteins in the plasma, the CEST peaks from **1** and **2** can be unambiguously observed and are comparable to those obtained in buffers.

While ions do not introduce interference to the stability of **1** and **2**, the Fe^II^Fe^III^/Fe^III^Fe^III^ redox couple (209 mV *vs.* NHE) makes oxidation of **2** in air a concern, which was studied by electronic absorption spectroscopy. A solution buffered at pH 7.4 containing 0.4 mM of **2** was prepared in a nitrogen glove box and exposed to air while a UV-Vis-NIR spectrum was recorded at 2 h intervals (see Fig. S38,† bottom). Over the course of 40 h, the absorption at 801 nm gradually disappeared, while the absorption at 470 nm shifted to *ca.* 460 nm and decreased in intensity. These spectral changes proceed through an isosbestic point at 445 nm, suggesting a clean conversion to a single, new species. Indeed, a similarly buffered solution containing 0.4 mM of **3** showed an identical UV-Vis-NIR spectrum to that of the 40 h oxidation product of **2** (see Fig. S38†), demonstrating that **2** is cleanly oxidized to the stable **3** in air. Moreover, the reversibility of this oxidation was demonstrated by *in situ* reduction of **3** by glutathione, as monitored by NMR spectroscopy (see Fig. S39 and S40†). This redox reversibility suggests the potential utilization of **3** as a probe precursor, which is stable in air and could undergo reduction to the CEST-active Fe^II^Fe^III^ upon introduction into the reducing extracellular environment of tissue.

To further examine the possibility of using **3** as a probe precursor, we carried out preliminary cell viability experiments using melanoma B16F10 cells as a model. After incubating the cells with media containing various concentrations of **3**, the percentages of viable cells were recorded (see Fig. S41†). In the presence of 8.2 mM of **3**, *ca.* 80% of cells are viable, and this percentage increased up to *ca.* 90% for samples containing lower concentrations of **3**. Overall >80% viability within millimolar probe concentration range is quite promising, as this is the concentration in which PARACEST probes show optimal contrast.

**Fig. 5:**
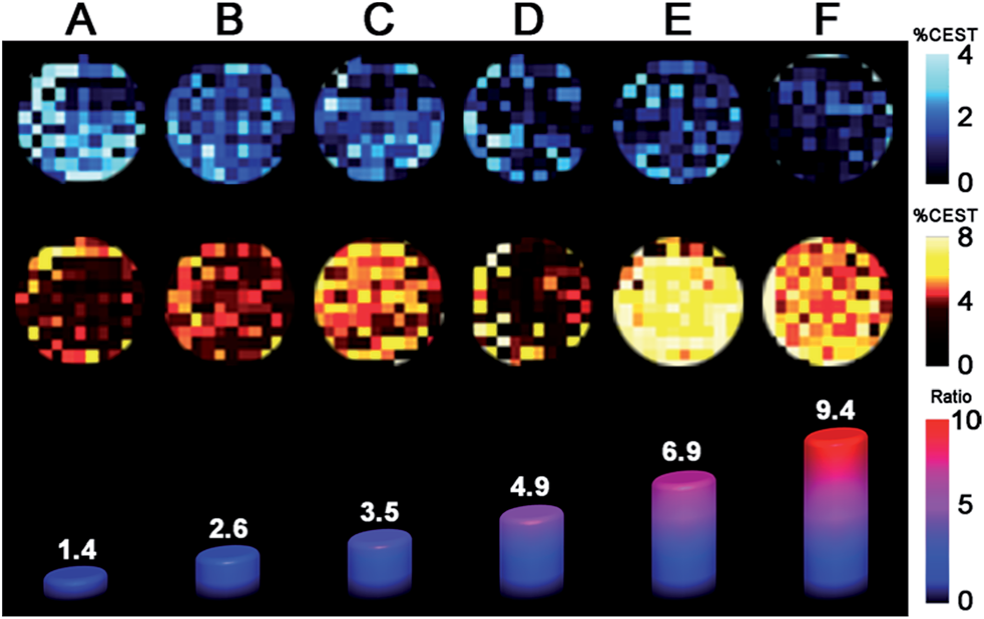
Phantom images of solutions containing 10 mM of Fe_2_ with **1** : **2** ratios ranging from 9 : 1 to 1 : 9 (A–F). Top and middle rows of images represent CEST effects with 14 μT presaturation at 40 and 83 ppm, respectively. The bars along the bottom row represent the ratio of average % CEST for presaturation at 83 and 40 ppm.

Finally, we sought to investigate whether the favorable CEST properties of the Fe_2_ probe observed on a 9.4 T NMR spectrometer could also be realized on phantom images from a 9.4 T preclinical MRI scanner. A series of solutions containing overall 10 mM Fe_2_ with **1** : **2** ratio ranging from 9 : 1 to 1 : 9 were prepared similarly to those in the NMR study. For each sample, two images were acquired with a 14 μT presaturation pulse applied at frequencies of 40 and 83 ppm from the H_2_O signal (see Fig. 5). Control images were acquired at the respective presaturation frequencies with 0 μT power. Presaturation at 40 and 83 ppm reduced the H_2_O intensity up to *ca.* 4 and 8%, respectively, demonstrating that CEST effects from both FeII2 and Fe^II^Fe^III^ redox states can be observed on a MRI scanner. However, the inhomogeneity of phantom intensities, likely stemming from weak CEST effects and therefore a noisy background, makes the contrast across phantoms virtually indistinguishable. Despite the ambiguous visualization of trend in redox status, the OCPs independently measured by a potentiostat can be plotted against the ratios between averaged phantom intensities from 83 and 40 ppm (CEST_83 ppm_/CEST _40 ppm_) to give a Nernstian fit resembling eqn (1)–(5) (see Fig. S42†). Furthermore, the OCPs calculated from the calibration, using intensities from phantom, fall in relatively good agreement with the OCPs measured by a potentiostat (see Table 1). Future efforts will aim to improve homogeneity of phantom images by increasing CEST through combination of chemical and pulse sequence optimizations.

**Table 1:** Comparison of OCP values obtained by Nernstian equation from CEST imaging *vs.* those obtained by potentiostat

OCP (*vs.* NHE)	A	B	C	D	E	F
CEST imaging	–263	–213	–188	–161	–133	–107
Potentiostat	–222	–201	–188	–170	–135	–101

## Conclusions

The foregoing results demonstrate the feasibility of using the Nernst equation to correlate OCP with the ratio of CEST effects from a Fe_2_ PARACEST probe, in a range spanning *ca.* –120 to –230 mV *vs.* NHE. To our knowledge, this study provides the first demonstration of ratiometric quantitation of solution redox status through NMR/MRI measurables. The CEST-active mixed-valence compound **2** is enabled by the presence of fast electron transfer and magnetic coupling to the neighboring fast-relaxing Fe^II^ center, as evidenced by NMR and electronic absorption studies. The potential applicability of the Fe_2_ probe is further highlighted by the potential utilization of the air-stable [FeIII2]^+^ complex as a one-electron oxidized probe precursor, which shows low cell-toxicity and excellent redox reversibility. Finally, a Nernstian calibration curve was constructed using averaged CEST effects from phantom images, and OCPs obtained from this curve are in good agreement with those obtained from a potentiostat.

Whereas the current Fe_2_ probe provides a promising proof-of-concept for quantitation of redox status, perhaps most exciting is that the dinucleating ligand scaffold provides an excellent platform for chemically tuning the Fe^II^Fe^II^/Fe^II^Fe^III^ redox couple. Toward this end, preliminary experiments show that the Fe^II^Fe^II^/Fe^II^Fe^III^ redox couple can be varied over a 120 mV range through either introduction of other bisphosphonate derivatives or chemical modification of the dinucleating ligand. Current work is geared toward tailoring members of this family of molecules to target optimal redox properties and proton exchange properties for *in vivo* applications.
