# Supplementary material for: Ratiometric quantitation of redox status with a molecular Fe2 magnetic resonance probe
Source: Chem Sci. 2017 Apr 19;8(6):4424–30. doi: 10.1039/c7sc00562h (PMC5580376; doi:10.1039/c7sc00562h)
Supplement: Supplementary file 1 [file SC-008-C7SC00562H-s001.pdf]

Electronic Supplementary Material (ESI) for Chemical Science.
This journal is © The Royal Society of Chemistry 2017

Electronic Supplementary Information for:

Ratiometric Quantitation of Redox Status with a Molecular Fe_2 Magnetic Resonance Probe

Kang Du[a], Emily A. Waters[b] and T. David Harris[a]*

[a]*Department of Chemistry, Northwestern University, 2145 Sheridan Road, Evanston, IL, USA, 60208-3113*
[b]*Center for Advanced Molecular Imaging, Northwestern University, 2145 Sheridan Road, Evanston, IL, USA, 60208-3113*

Email: dharris@northwestern.edu

Chem. Sci.

Table of Contents

Experimental Section **S3**
Table S1 | Crystallographic data for LFe_2(etidronate)·$7H_2O$ **S8**
Figure S1 | CV of **1** in pH 7.4 buffer solution **S9**
Figure S2 | Variable temperature Mössbauer spectra of **2** **S10**
Figure S3 | Mössbauer spectrum of **1** at 80 K **S11**
Figure S4 | UV-vis-NIR spectra of **1** and **2** in neutral D_2O **S12**
Figure S5 | Diffuse reflectance spectrum of **2** **S13**
Figure S6 | Variable temperature dc susceptibility data for **1** and **2** **S14**
Figure S7 | NMR spectra of **1** in neutral H_2O and D_2O **S15**
Figure S8 | NMR spectra of **2** in neutral H_2O and D_2O **S16**
Figure S9 | CEST spectra for **1** and **2** in pH 7.4 buffer **S17**
Figure S10 | Omega plot of **1** in pH 7.4 buffer **S18**
Figure S11 | Omega plot of **2** in pH 7.4 buffer **S19**
Figure S12 | Open circuit potential equilibration time in pH 7.4 buffer **S20**
Figure S13 | Open circuit potential equilibration time in pH 7.5 buffer **S21**
Figure S14 | Open circuit potential equilibration time in pH 7.3 buffer **S22**
Figure S15 | Open circuit potential equilibration time in pH 7.4 buffer (phantom) **S23**
Figure S16 |CEST spectrum for a mixture of **1** and KO_2 **S24**

Figure S17 | CEST spectrum for a mixture of **2** and cysteine **S25**
Figure S18 | Open circuit potential equilibration time for oxidation/reduction of **1**/**2** **S26**
Figure S19 | CEST spectra for **1** and **2** mixtures in pH 7.3 buffer **S27**
Figure S20 | Open circuit potential calibration curve at pH 7.3 **S28**
Figure S21 | CEST spectra for **1** and **2** mixtures in pH 7.5 buffer (37 °C) **S29**
Figure S22 | Open circuit potential calibration curve at pH 7.5 (37 °C) **S30**
Figure S23 | Nernstian calibrations at pH of various values **S31**
Figure S24 | CEST spectra for **1** and **2** mixtures in pH 7.5 buffer (35 °C) **S32**
Figure S25 | Open circuit potential calibration curve at pH 7.5 (35 °C) **S33**
Figure S26 | CEST spectra for **1** and **2** mixtures in pH 7.5 buffer (39 °C) **S34**
Figure S27 | Open circuit potential calibration curve at pH 7.5 (39 °C) **S35**
Figure S28 | Nernstian calibrations at various temperatures at pH 7.5 **S36**
Figure S29 | NMR spectra of **1** with/without presence of anions (H_2O buffer) **S37**
Figure S30 | NMR spectra of **1** with/without presence of anions (D_2O) **S38**
Figure S31 | NMR spectra of **2** with/without presence of anions (H_2O buffer) **S39**
Figure S32 | NMR spectra of **2** with/without presence of anions (D_2O) **S40**
Figure S33 | NMR spectra of **1** with/without presence of Ca^{2+} (H_2O buffer) **S41**
Figure S34 | NMR spectra of **1** with/without presence of Ca^{2+} (D_2O) **S42**
Figure S35 | NMR spectra of **2** with/without presence of Ca^{2+} (H_2O buffer) **S43**
Figure S36 | NMR spectra of **2** with/without presence of Ca^{2+} (D_2O) **S44**
Figure S37 | CEST spectra for **1** and **2** in bovine blood plasma at pH 7.4 **S45**
Figure S38 | UV-vis-NIR spectra of **2** oxidized in air and **3** in pH 7.4 buffer **S46**
Figure S39 | NMR spectra of **3** with/without presence of glutathione (H_2O buffer) **S47**
Figure S40 | NMR spectra of **3** with/without presence of glutathione (D_2O) **S48**
Figure S41 | Cell viability of **3** **S49**
Figure S42 | Open circuit potential calibration curve at pH 7.4 (phantom) **S50**
Figure S43 | IR spectra for **1**, **2** and **3** **S51**
References **S52**

Experimental Section

General considerations. Unless otherwise specified, chemicals and solvents were purchased from commercial vendors and used without further purification. Deuterated solvents were purchased from Cambridge Isotope Laboratories. Bovine blood plasma was obtained from commercial sources (Sigma Aldrich lot P4639). When necessary for moisture sensitive experiments, glassware was flame dried or stored in an oven at 150 °C for at least 4 hours, followed by cooling in a desiccator. Air- and water-free manipulations were carried out using standard Schlenk techniques. Acetonitrile was dried using a commercial solvent purification system from Pure Process Technology and stored over 4 Å molecular sieves prior to use. Water was obtained from a purification system from EMD Millipore. Elemental analysis was conducted by Midwest Microlab Inc. Preparative reverse-phase HPLC was performed on a Waters 19 × 250 mm^2 Xbridge C18 Column, using the Varian Prostar 500 system equipped with a Varian 363 fluorescence detector and a Varian 335 UV/Visible Detector. During HPLC, water was used as solvent A and acetonitrile as solvent B. The absorbances at 220 and 285 nm were monitored. Anhydrous hydrogen chloride gas was generated by adding concentrated hydrochloric acid to a stirring solution of concentrated sulfuric acid. The gas was passed through a bubbler filled with sulfuric acid. Synthesis of *N*,*N′*-[(2-hydroxy-5-methyl-1,3-phenylene)bis(methylene)]bis[N-(carboxymethyl)glycinamide] (HL) was based off a procedure reported previously.[1]

Synthesis of (NMe_4)[LFe_2L']·2.7H_2O·THF (1). To a stirred suspension of HL (20 mg, 0.051 mmol) in MeOH (2 mL) was added dropwise a solution of anhydrous $FeCl_2$ (13 mg, 0.10 mmol) in MeOH (2 mL) to give a light purple solution. A solution of etidronic acid monohydrate (11.4 mg, 0.051 mmol) in MeOH (2 mL) was then added dropwise to give a light orange solution, followed by addition of $NMe_4OH{\cdot}5H_2O$ (46 mg, 0.26 mmol) in MeOH (2 mL) to give a light yellow slurry. After stirring for 12 h, the yellow solid was collected by filtration and washed with MeOH (10 mL). The solid was then stirred in THF (15 mL) for 20 min, collected by filtration, and dried under reduced pressure for 12 h to give **1** (20 mg, 50%). Solution magnetic moment $\chi_M T$ = 7.3(3) cm^3K/mol (310 K). Anal. Calcd. for $C_{27}H_{54.4}Fe_2N_7O_{15.7}P_2$: C, 35.99; H, 6.08; N, 10.87. Found: C, 36.00; H, 6.15; N, 10.91. ICP-OES: Fe:P = 0.96:1. UV-Vis absorption spectrum: 453 nm (ε = 48 M^{-1} cm^{-1}). FT-IR (ATR, cm^{-1}): 3289 (m, broad); 3158 (m, broad); 1668 (s); 1615 (s); 1475 (s); 1447 (w); 1311 (s); 1262 (w); 1097 (s); 1055 (s); 991 (m); 877 (m); 802 (w); 660 (m); 560 (s) (see Figure S43).

Synthesis of LFe_2L'·0.7H_2O·0.2THF (2). To a stirred suspension of HL (20 mg, 0.051 mmol) in MeOH (2 mL) was added dropwise a solution of anhydrous $FeCl_3$ (8.2 mg, 0.051 mmol) in MeOH (2 mL) to give a dark purple solution. Anhydrous $FeCl_2$ (6.4 mg, 0.051 mmol) in MeOH (2 mL) was then added dropwise to this solution, with no significant color change observed. A solution of etidronic acid monohydrate (11 mg, 0.051 mmol) in MeOH (2 mL) was then added dropwise, resulting in a dark red solution. A solution of $NMe_4OH{\cdot}5H_2O$ (46 mg, 0.26 mmol) in MeOH (2 mL) was then added dropwise to give a red slurry. After stirring for 12 h, the a red-brown solid was collected by filtration and was washed with MeOH (10 mL). The solid was then stirred in THF (15 mL) for 20 min, collected by filtration, and dried under reduced pressure for 12 h to give **2** (34 mg, 95%). Solution magnetic moment $\chi_M T$ = 7.0(6) cm^3K/mol (310 K). Anal. Calcd. for $C_{19.8}H_{32}Fe_2N_6O_{12.9}P_2$: C, 32.40; H, 4.39; N, 11.45. Found: C, 32.40; H, 4.27; N, 11.38.

ICP-OES: Fe:P = 0.97:1. UV-Vis absorption spectrum: 470 nm (ε = 861 M^{-1} cm^{-1}), 801 nm (ε = 168 M^{-1} cm^{-1}), 1383 nm (ε = 83 M^{-1} cm^{-1}). FT-IR (ATR, cm^{-1}): 3278 (m, broad); 2969 (m, broad); 1655 (s); 1613 (s); 1479 (m); 1453 (w); 1311 (m); 1263 (m); 1120 (s); 1049 (s); 988 (s); 877 (m); 805 (w); 662 (m); 562 (s) (see Figure S43). Slow diffusion of THF vapor into a concentrated solution of **2** in H_2O over the course of 3 d gave dark red plate-shaped crystals of LFe_2(etidronate)·7H_2O suitable for single-crystal X-ray diffraction analysis.

Synthesis of [LFe_2L'](NO_3)·0.9H_2O·1.5THF (3). To a stirred suspension of HL (20 mg, 0.051 mmol) in MeOH (2 mL) was added dropwise a solution of $Fe(NO_3)_3$·9H_2O (41 mg, 0.10 mmol) in MeOH (2 mL) to give a dark purple solution. A solution of etidronic acid monohydrate (11 mg, 0.051 mmol) in MeOH (2 mL) was then added dropwise to give a red solution. A solution of NMe_4OH·5H_2O (46 mg, 0.26 mmol) in MeOH (2 mL) was then added dropwise give a red slurry. After stirring for 12 h, the ensuing red solution was dried under reduced pressure to give a red solid. The solid was stirred in DMF (10 mL) for 30 min, and was then collected by filtration and washed with THF (10 mL) and Et_2O (10 mL). The residue was dried under reduced pressure for 2 h to give **3** (20 mg, 51%) Solution magnetic moment $\chi_M T$ = 8.9(3) cm^3K/mol (310 K). Anal. Calcd. for $C_{20.5}H_{36.7}Fe_2N_7O_{17.4}P_2$: C, 29.52; H, 4.44; N, 11.78. Found: C, 29.52; H, 4.53; N, 11.84. ICP-OES: Fe:P = 1.1:1. FT-IR (ATR, cm^{-1}): 3110 (m, broad); 1652 (s); 1591 (s); 1477 (m); 1417 (w); 1386 (m); 1315 (m); 1095 (m); 1001 (s); 880 (m); 800 (m); 664 (w); 573 (m); 468 (s); 448 (m); 414 (m) (see Figure S43).

X-ray structure determination. A single crystal of LFe_2(etidronate)·7H_2O was directly coated with Paratone-N oil and mounted on a MicroMounts™ rod. The crystallographic data were collected at 100 K on a Bruker APEX II diffractometer equipped with MoKα sealed tube source. Raw data were integrated and corrected for Lorentz and polarization effects using Bruker APEX2 v. 2009.1.[2] The program SADABS was used to apply absorption correction.[3] Space group assignments were determined by examining systematic absences, E-statistics and successive refinement of the structure. Structures were solved by SHELXT[4] using direct methods and refined by SHELXL within the OLEX interface.[(3)] Partially occupied solvent H_2O molecules that were potentially hydrogen bonded were modeled isotropically. Thermal parameters for all other non-hydrogen were refined anisotropically. Crystallographic data and the details of data collection are listed in Table S1.

^{1}H NMR experiments. Variable temperature ^{1}H NMR spectra were collected on an Agilent DD MR-400 system (9.4 T) system. The T_1 of H_2O was obtained by fitting H_2O intensities from experiments with an array of relaxation times implemented in the program *vnmr*. Linewidth analyses were obtained in the program MNOVA.

CEST experiments. Variable temperature CEST experiments were performed on an Agilent DD MR-400 system (9.4 T) system. In a typical experiment, samples containing 100% **1**, 100% **2** or mixture of the two at a desired ratio in buffer containing 100 mM NaCl and 100 mM of HEPES at pH desired were used for CEST experiments. Z-spectra (CEST spectra) were obtained according to the following protocol. NMR spectra were acquired using the presaturation pulse applied for 7 s at a power level of 24 μT. The saturation frequency offsets were screened with a step increase of 1 ppm. The obtained NMR spectra were plotted as normalized water intensity

against frequency offset to produce a Z-spectrum. Direct saturation of the water signal was set to 0 ppm. D_2O was placed in an inner capillary to lock the sample. Exchange rate constants were calculated based off a reported method.[5] The B_1 values are calculated based on the calibrated 90-degree pulse on a linear amplifier. The NMR spectra were acquired at various presaturation powers ranging from 14 to 24 μT applied for 7 s. To correct for baseline, reported values of %CEST are the difference in percent H_2O signal reduction between applied on-resonance and off-resonance pre-saturations.

Solid state magnetic measurements. Magnetic measurements were carried out using a Quantum Design MPMS-XL SQUID magnetometer. Powder samples were sealed in 2 mL polyethylene bags. Dc susceptibility data were collected from 1.8 to 300 K at applied dc fields of 1, 1.5 and 2 T. Dc susceptibility data were corrected for diamagnetic contribution from the sample holders and from the sample (estimated using Pascal's constants[6]). The temperature dependent magnetic susceptibility data for **1** (10-300 K) and **2** (1.8-300 K) and were model using spin Hamiltonian $\hat{H} = -2J(\hat{S}_{Fe1} \cdot \hat{S}_{Fe2})$,[7] where J is the magnetic superexchange coupling constant; and $\hat{S}_{Fe1}$ and $\hat{S}_{Fe2}$ are the spin operators for the Fe ions. The best fits of the data give g = 2.20(3) and 2.00(4) for **1** and **2**, respectively.

Solution magnetic measurements. Magnetic moments of metal complexes were carried out using Evan's method[8] at 310 K. In a typical experiment, compounds (about 4 mM) were dissolved in a mixture of 0.5 w/w % of DMSO in D_2O. A capillary containing same solvent mixture (without the to-be-characterized compound) was inserted into each NMR sample as reference. Diamagnetic correction was carried out based on the empirical formula of each compound (as determined by elemental analysis) using Pascal's constants.[6]

Electrochemical measurements. Cyclic voltammetry was carried out in a standard one-compartment cell inside a nitrogen glove box at room temperature, equipped with a platinum working electrode, a platinum wire as counter electrode and a SCE reference electrode using a CHI 760c potentiostat. The analyte solution was with 100 mM NaCl and 100 mM HEPES buffered at pH 7.4. The voltammogram was converted and shown as values referred to the normal hydrogen electrode (NHE), using a literature conversion factor.[9] Open circuit potentials were measured by the built-in technique "open circuit potential – time" within the CHI660E electrochemical workstation software. The open circuit potential readings were recorded 10 minutes after the experiment started, at which time the reading was stabilized.

Mössbauer spectroscopy. Zero-field ^{57}Fe Mössbauer spectra were obtained at various temperatures with a constant acceleration spectrometer and a ^{57}Co/rhodium source. Prior to measurements, the spectrometer was calibrated at 295 K with α-iron foil. Samples were prepared in an MBraun nitrogen glove box. A typical sample contained approximately 60 mg of compounds (~10 mg of Fe) suspended in a plastic cap in heated eicosane, which solidified upon cooling to ambient temperature, in order to immobilize the sample. Another cap with a slightly smaller diameter was squeezed into the previous sample cap to completely encapsulate the solid sample mixture. All spectra were analyzed using the WMOSS Mössbauer Spectral Analysis Software (www.wmoss.org).

Other physical measurements. Infrared spectra were recorded on a Bruker Alpha FTIR spectrometer equipped with an attenuated total reflectance accessory. Solution and solid-state UV-vis-NIR spectra were obtained using an Agilent Cary 5000 spectrophotometer.

Estimation of electron transfer rate by IVCT analysis.[10] The calculation of ambient temperature electron-transfer rate in **2** is based on a method described in a similar mixed-valence Fe_2 analog. Location of the IVCT ν_{max}, extinction coefficient (ε) and Fe···Fe distance (d) were obtained experimentally as described in the main text. The full width at half maximum ($\Delta\nu_{1/2}$) was determined by fitting IVCT to a Gaussian model in the software OriginPro. The electron-transfer rate (k_{et}) in **2** can be calculated using the following equation:

$$k_{et} = \nu_{et}\exp(-\Delta G^*/RT)$$

where R is the ideal gas constant and T is temperature. The frequency factor for electron transfer, ν_{et}, and the thermal free energy, ΔG^*, are given by:

$$\nu_{et} = 2\pi^{3/2}\nu_{ab}h^{-1}(kT\nu_{max})^{-1/2} \text{ and } \Delta G^* = \nu_{max}(4 - \nu_{ab})^{-1}$$

where h is the Planck constant, k is the Boltzmann constant and ν_{max} is the wavenumber of the IVCT peak maximum. The resonance matrix element, ν_{ab}, is given by:

$$\nu_{ab} = \alpha\nu_{max},$$

where the extent of electron delocalization $\alpha^2 = 4.2 \times 10^{-4}\varepsilon\Delta\nu_{1/2}(\nu_{max}d^2)^{-1}$. Here, ε is the extinction coefficient of IVCT, and d is the Fe···Fe distance determined by X-ray structural analysis.

Viability experiment. Melanoma B16F10 cells (ATCC) were cultured in Dulbecco's Modified Eagle's Media (Life Technologies) supplemented with 10% fetal bovine serum (Fisher), 1 mM each of sodium pyruvate, non-essential amino acids and L-glycine at 37 °C and 5% CO_2. Cells for the experiment were subcultivated for 3 to 4 times after thawing the cell stocks. Cells were incubated with media containing the desired concentration of **3** for 24 h before viability measurements. Cell viability was measured by a Guava EasyCyte Mini Personal Cell Analyzer (EMD Millipore). Each sample subjected for analysis contained 50 μL of well-mixed cell suspension and 150 μL of Guava ViaCount reagent. Stained samples were vortexed for 20 s and immediately subjected to counting using the ViaCount software module. Viability was measured using the EasyFit software module. Cells not treated by **3** were used as a control to account for normal cell death. Reported %viability was normalized with respect to the control samples.

MRI phantom experiment. Samples contained 100 mM of NaCl, 100 mM of HEPES buffered at pH 7.4 and overall 10 mM Fe_2 concentration with **1**:**2** ratio ranging from 9:1 to 1:9. ~0.5 mL of each sample was stored in a 0.5 mL Eppendorf tube, which was placed within another scintillation vial filled with H_2O solution containing 1 mg/mL $CuSO_4$ and 100 mM NaCl for T_1 matching. CEST experiments were carried out on a Bruker Biospec 9.4 T MRI scanner running ParaVision 6.0.1 (Bruker Biospin, Billerica, MA, USA). Temperature was maintained at 37 °C using a warm water circulating system with feedback control from a temperature probe (SA Instruments, Stonybrook, NY, USA). CEST images were acquired using an accelerated spin echo based sequence with a pre-saturation pulse (14 μT, 2 s duration) applied at offsets of 83 and 40 ppm (M_z). Other imaging parameters: TR/TE = 2034/14.9 ms, RARE factor 16, matrix = 64 × 64,

FOV = 3.2 × 3.2 cm, 2 mm slice thickness, and 2 averages). Matched unsaturated images were acquired using identical parameters except that the pulse amplitude was set to 0 μT (M_0). %CEST = (1 – M_z/M_0) × 100%. Only regions of the inner Eppendorf tube, where the sample containing the Fe_2 probe is shown in Figure 5. Averaged intensities of the same regions were used to calculate $CEST_{83\ ppm}/CEST_{40\ ppm}$ for fitting. For the fitting, sample A was a significant outlier likely due to weak CEST signal, and therefore was not taken into account for fitting. Trace amounts of precipitation occurred for sample D, E, F during the phantom experiment, likely due to the affected solubility of **1** and/or **2** in the presence of high buffer concentration. Such precipitate did not alter either the phantom experiment or OCP measurement.

Table S1 | Crystallographic data for LFe_2(etidronate)·$7H_2O$

	**LFe_2(etidronate)·$7H_2O$**
Empirical formula	$C_{19}H_{38}Fe_2N_6O_{19}P_2$
Formula weight, g mol^{-1}	828.19
Crystal system	Orthorhombic
Space group	$Pna2_1$
Wavelength, Å	0.71073
Temperature, K	100
a, Å	21.3916(7)
b, Å	9.4099(3)
c, Å	16.1292(5)
α, °	90
β, °	90
γ, °	90
V, Å^3	3246.7(2)
Z	4
ρ_{calcd}, Mg m^{-3}	1.694
μ, mm^{-1}	1.081
Reflections coll./unique	47460/45560
R_{int}	0.0293
$^aR_1$ (I >2$\sigma(I)$)	0.0497
$^bwR_2$ (all)	0.1170
GooF	1.2370

$^aR_1 = \Sigma||F_0| - |F_C||/\Sigma|F_0|$

$^bwR_2 = [\Sigma w(F_0^2 - F_C^2)^2/\Sigma w(F_o^2)^2]^{1/2}$

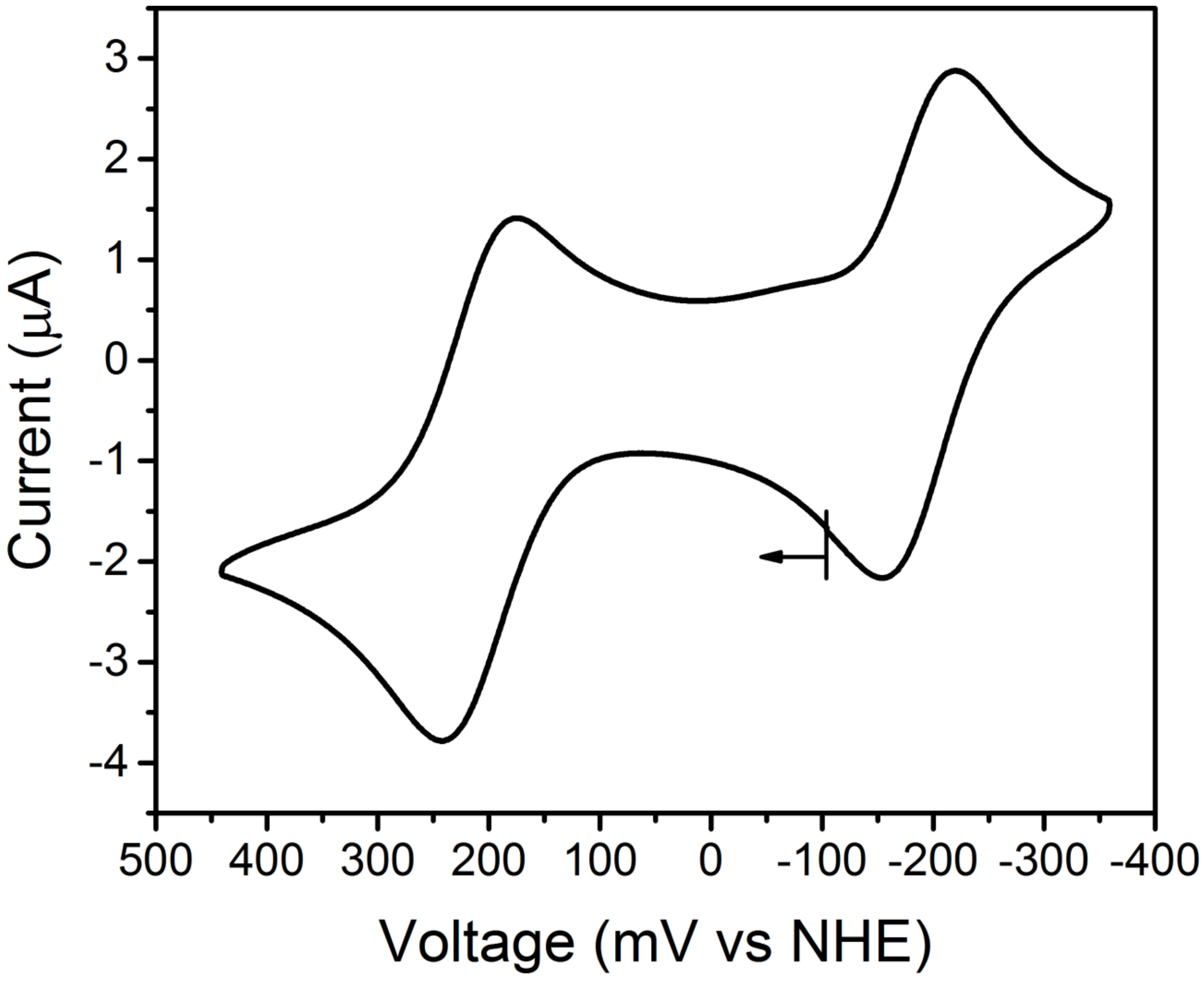

Figure S1. CV of **1** in solution containing 100 mM NaCl, 100 mM HEPES, buffered at pH 7.4. 50 mV/s scan rate.

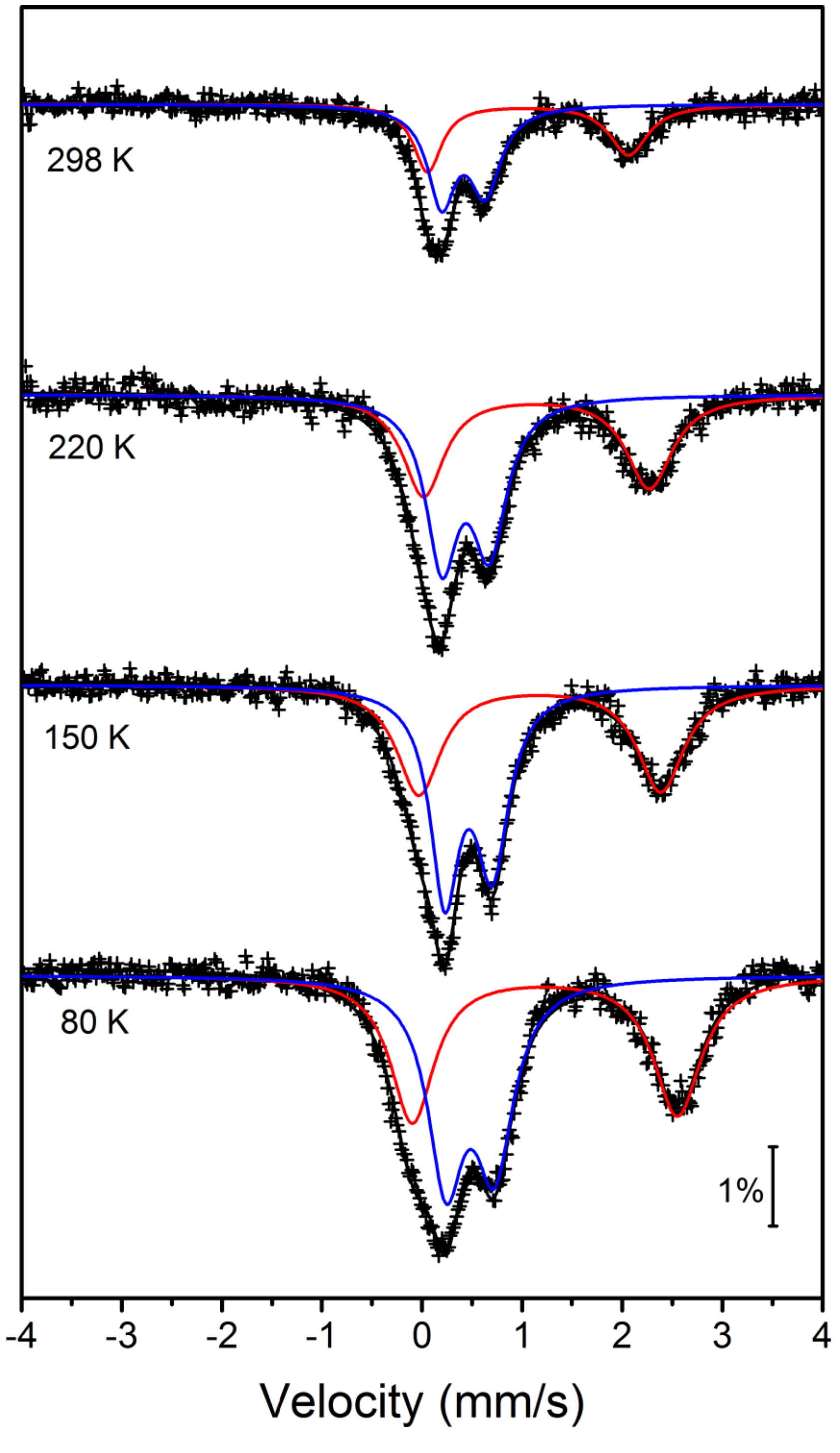

Figure S2. Variable temperature Mössbauer spectra of **2**. Crosses, black line, red line and blue line represent the experimental data, global fit, fit of high spin Fe^{II} and fit of high spin Fe^{III}, respectively.

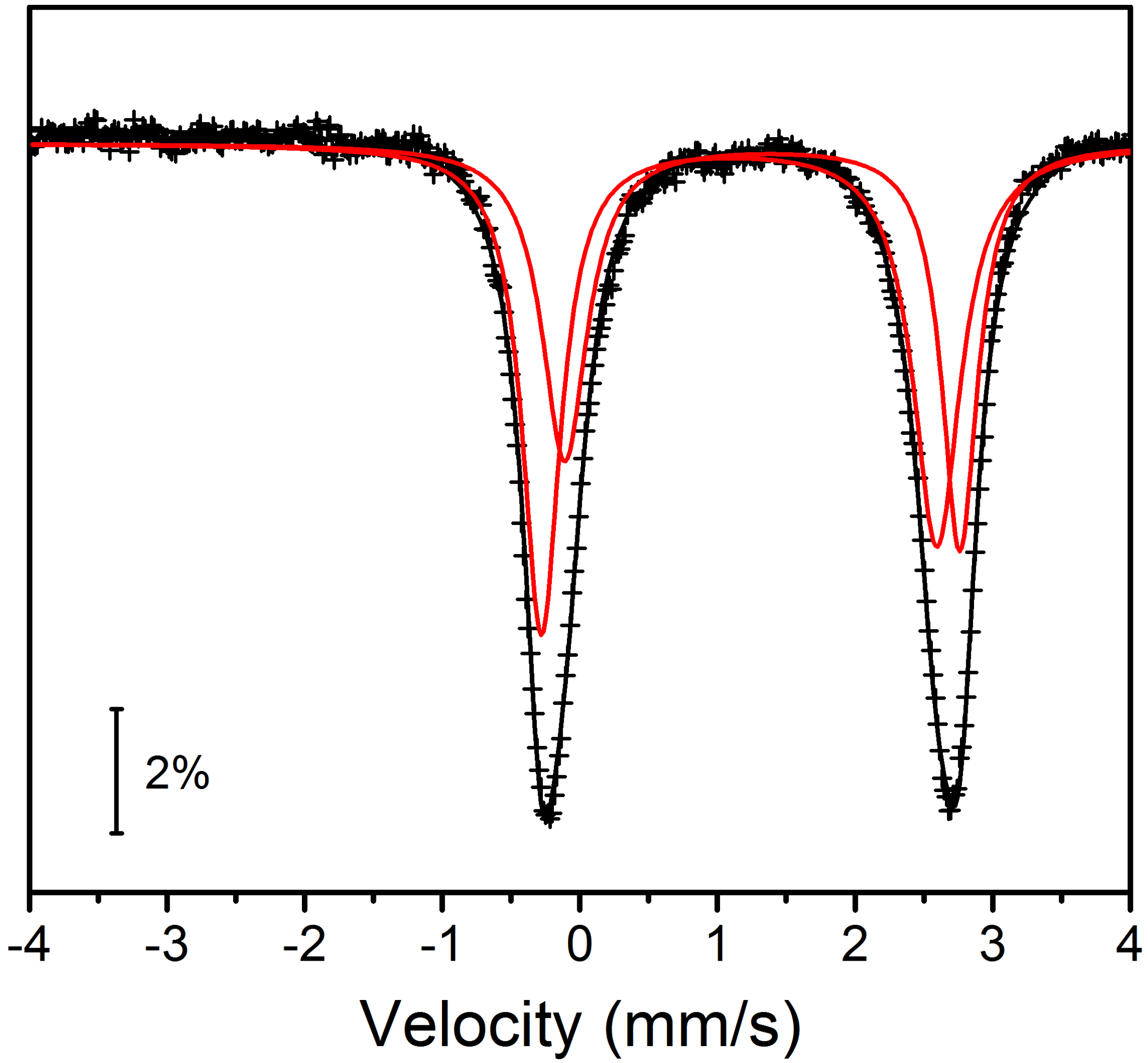

Figure S3. Mössbauer spectrum of **1** at 80 K. Crosses and red line represent the experimental data and fit, respectively.

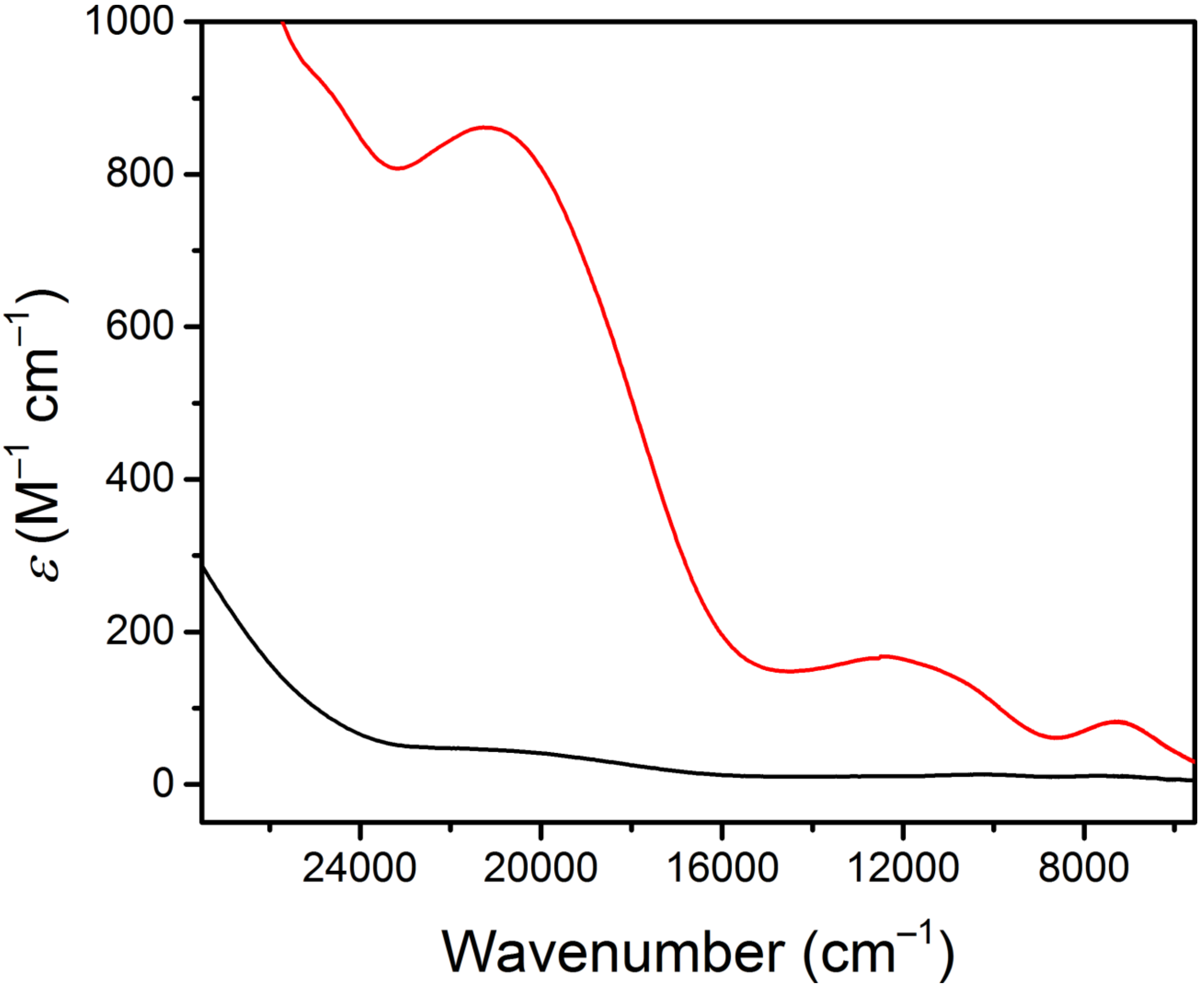

Figure S4. UV-vis-NIR spectra of **1** (black) and **2** (red) in D_2O.

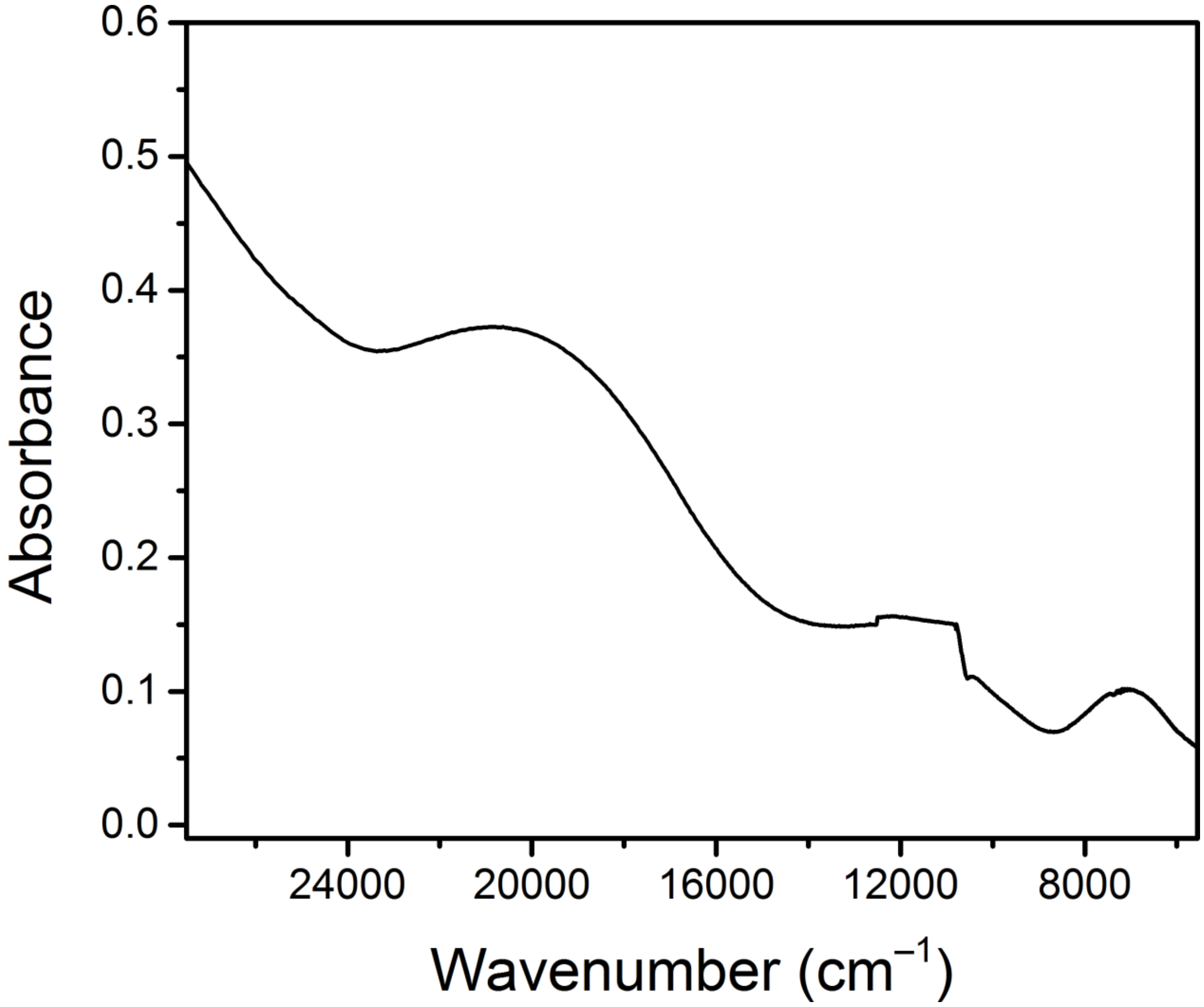

Figure S5. Diffuse reflectance spectrum of **2**. The flat feature at ca. 12000 cm^{-1} is an instrument artifact.

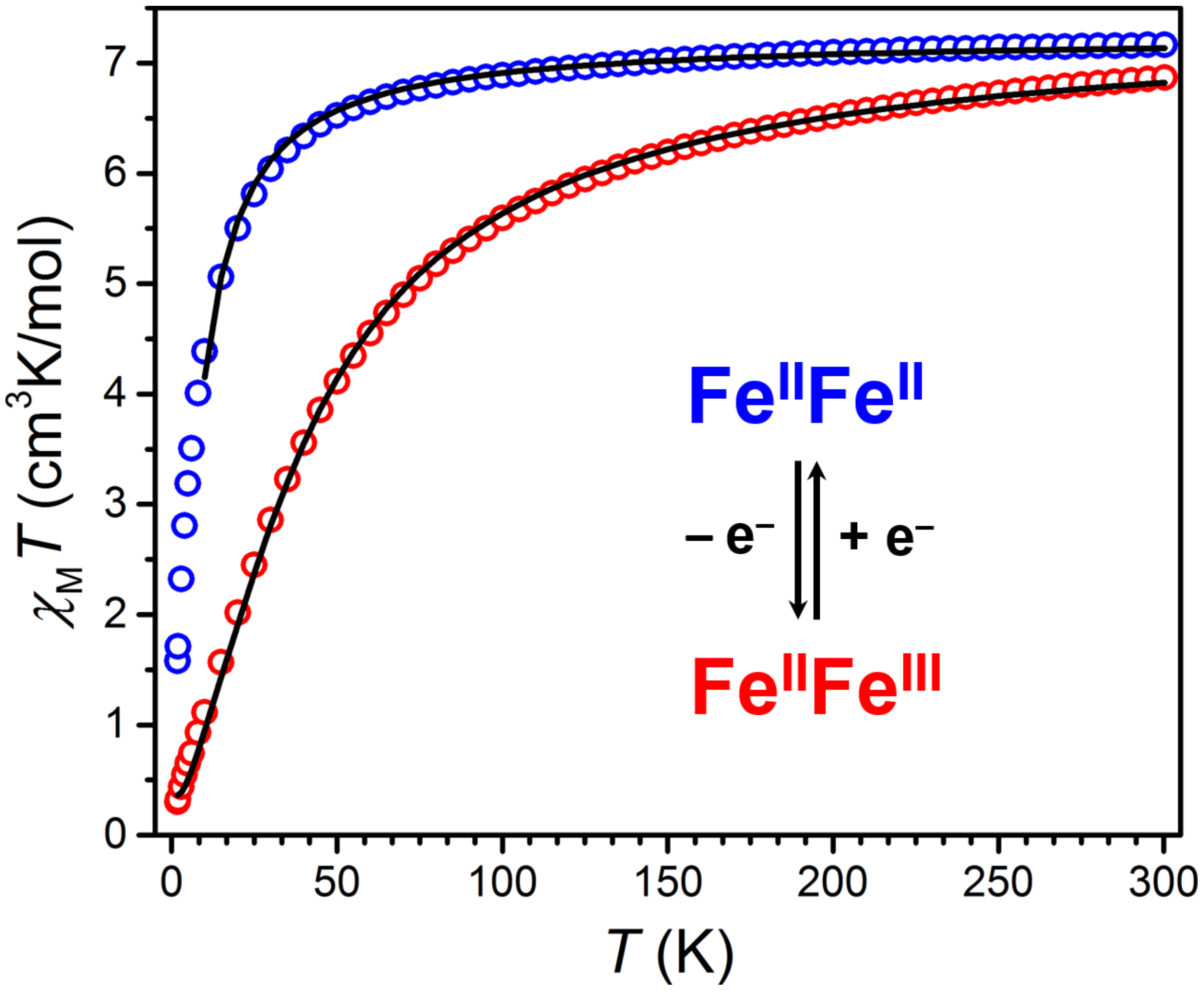

Figure S6. Variable temperature dc magnetic susceptibility data for **1** (blue circles) and **2** (red circles) collected under an applied field of 1 T. The black lines correspond to fits of the data.

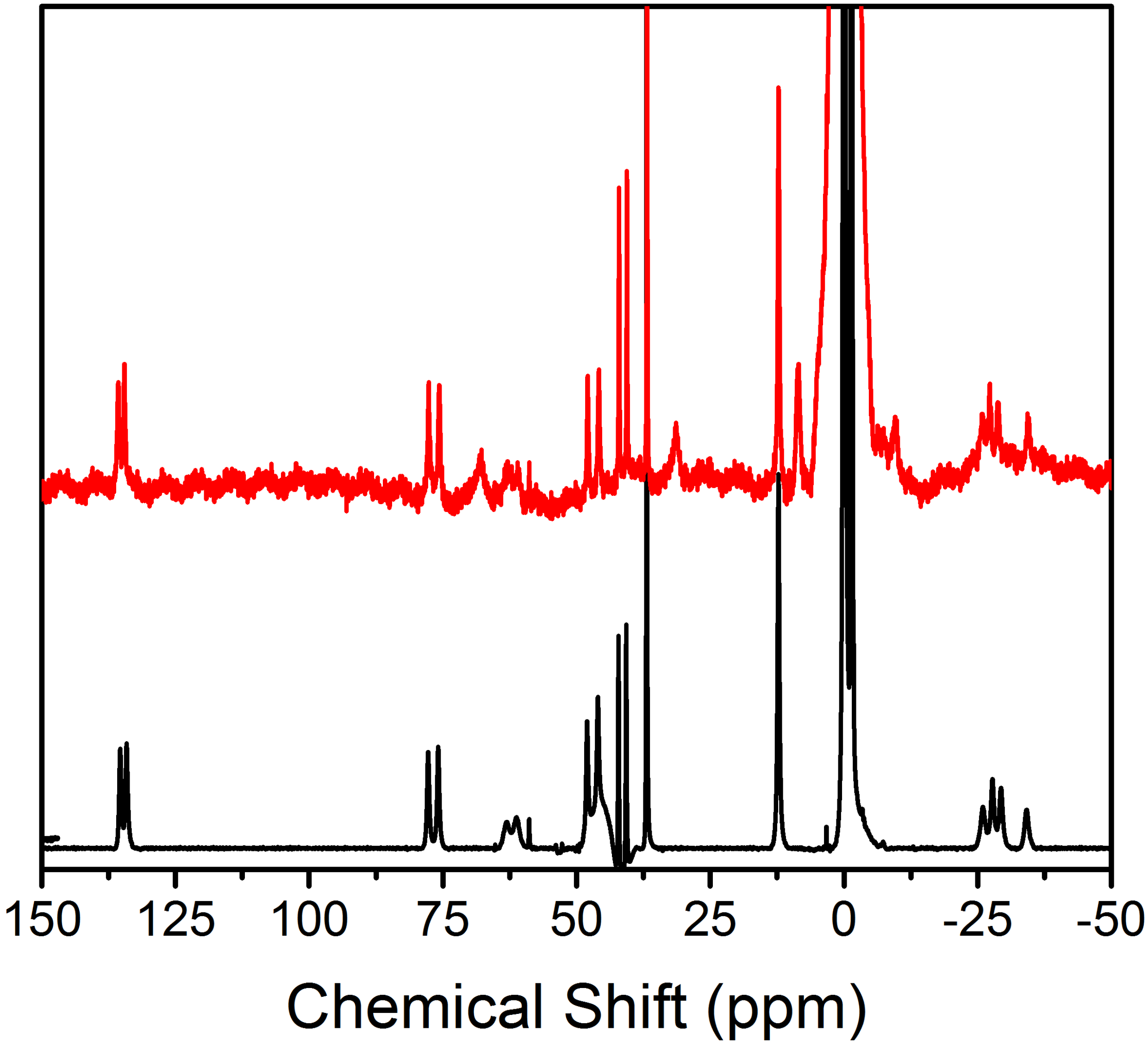

Figure S7. Stacked NMR spectra of **1** in neutral H_2O (red) and D_2O (black) at 37 °C.

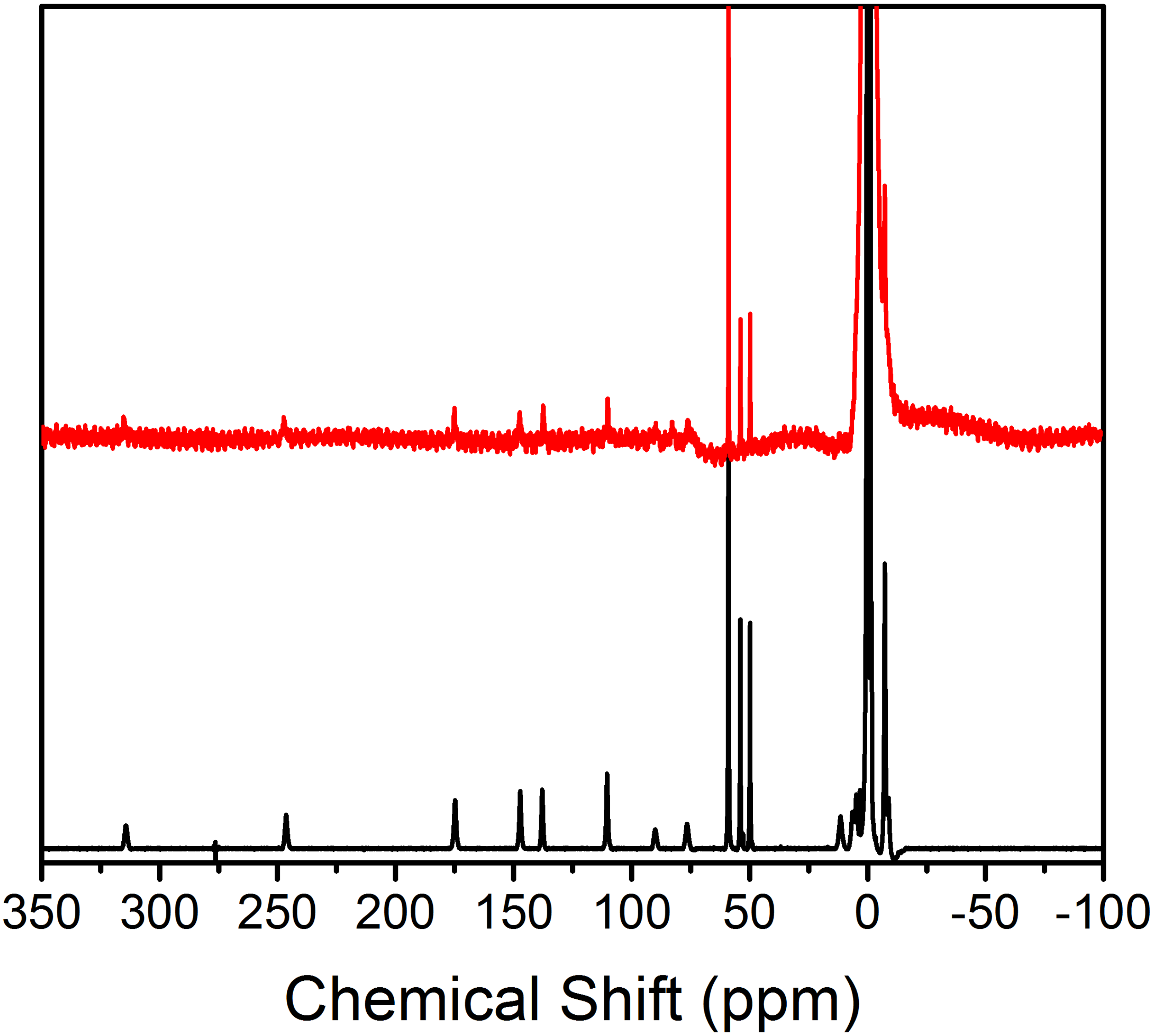

Figure S8. Stacked NMR spectra of **2** in neutral H_2O (red) and D_2O (black) at 37 °C.

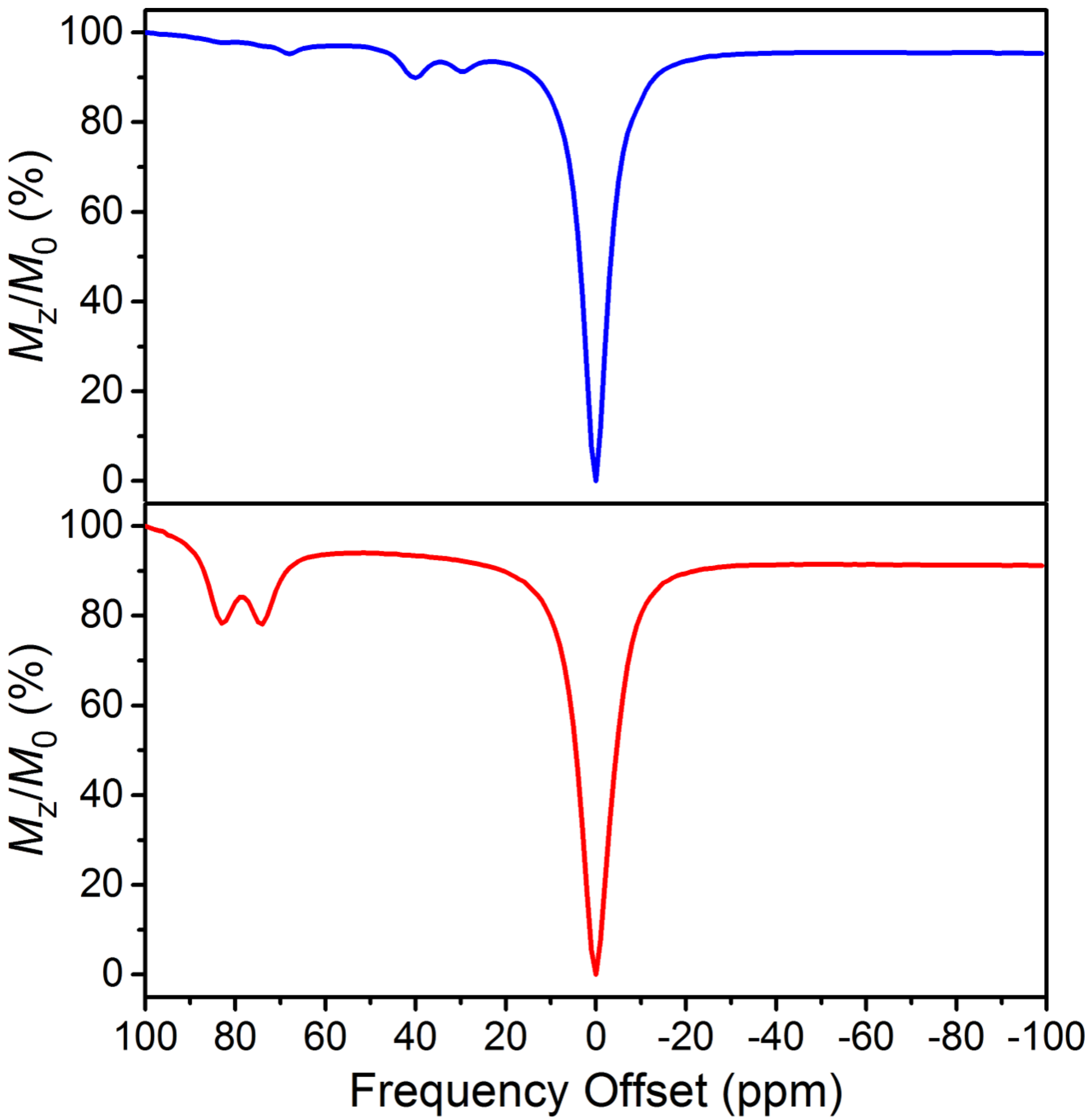

Figure S9. CEST spectra collected at 37 °C for solutions containing 4.9 mM of **1** (top) and **2** (bottom) with 100 mM HEPES and 100 mM NaCl buffered at pH 7.4.

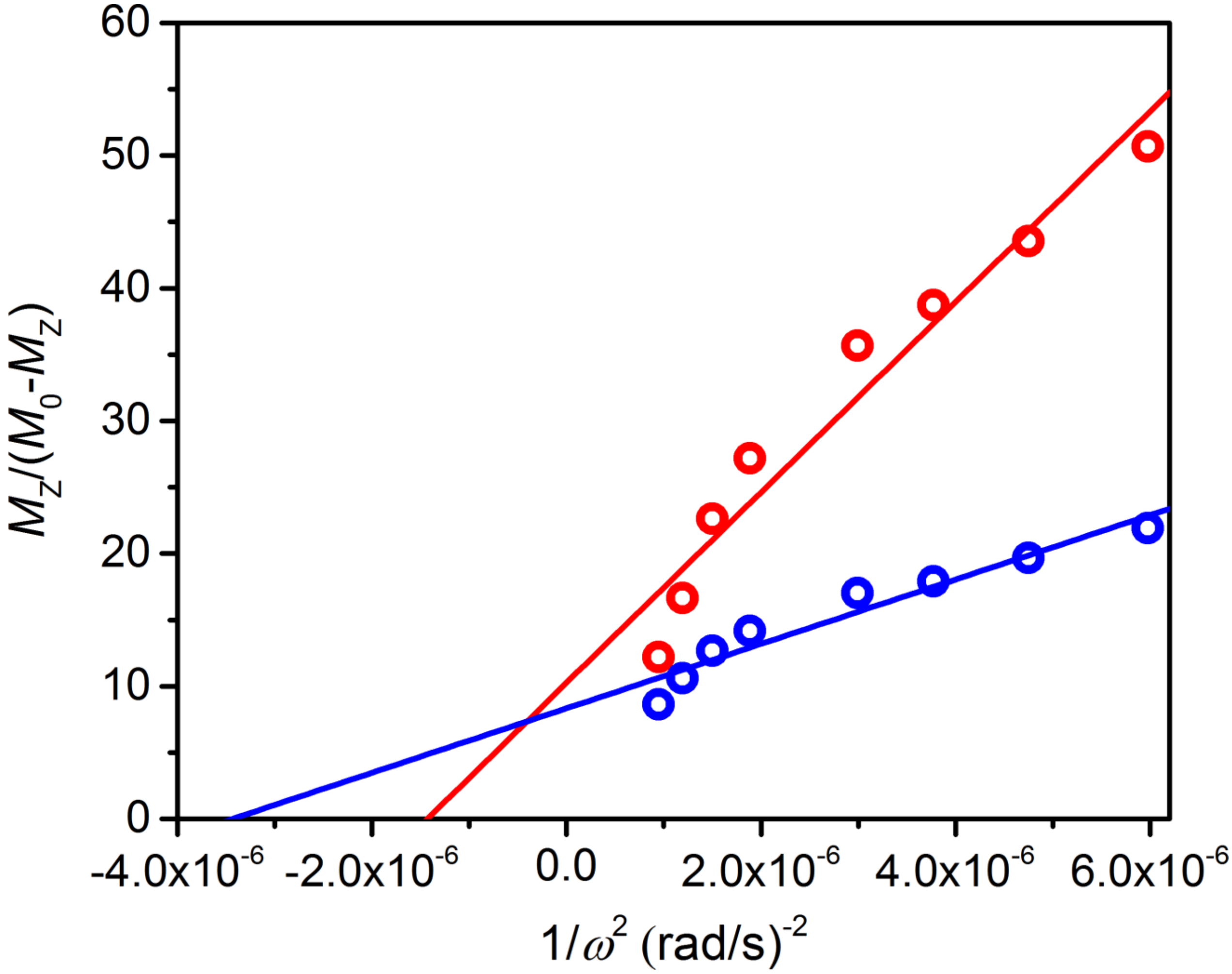

Figure S10. Omega plot of the CEST effect at 29 ppm (red) and 40 ppm (blue) of 4.0 mM **1** in pH 7.4 H_2O with 100 mM HEPES and 100 mM NaCl. Circles represent experimental data and the lines represent the linear fit.

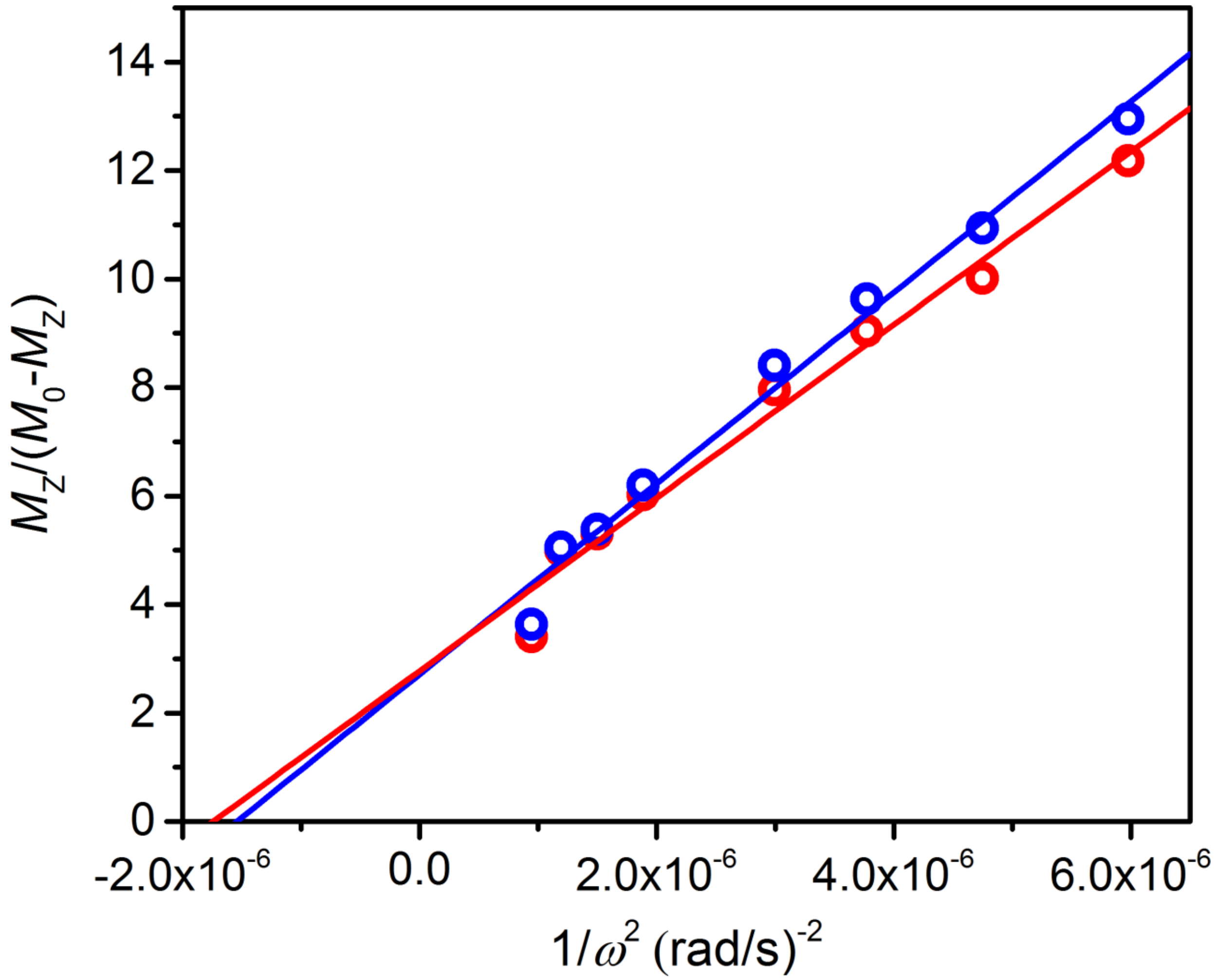

Figure S11. Omega plot of the CEST effect at 74 ppm (red) and 83 ppm (blue) of 4.0 mM **2** in pH 7.4 H_2O with 100 mM HEPES and 100 mM NaCl. Circles represent experimental data and lines represent linear fits.

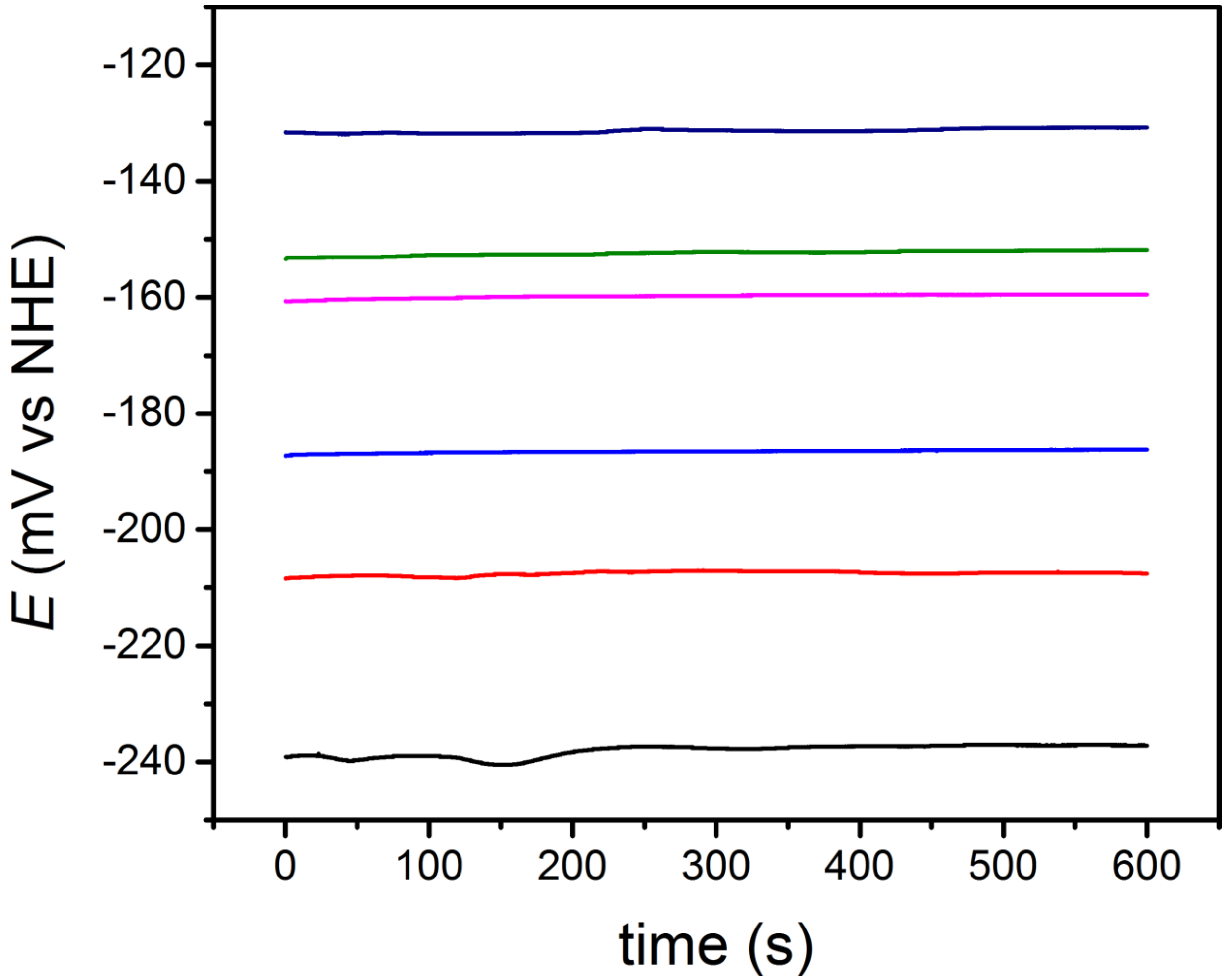

Figure S12. OCPs of solutions containing overall 4.9 mM Fe_2, with the ratio of **1**:**2** ranging from 9:1 (bottom most) to 1:9 (top most), are monitored over time upon mixing (at 0 s). Each solution contains 100 mM NaCl and 100 mM HEPES buffered at pH 7.4.

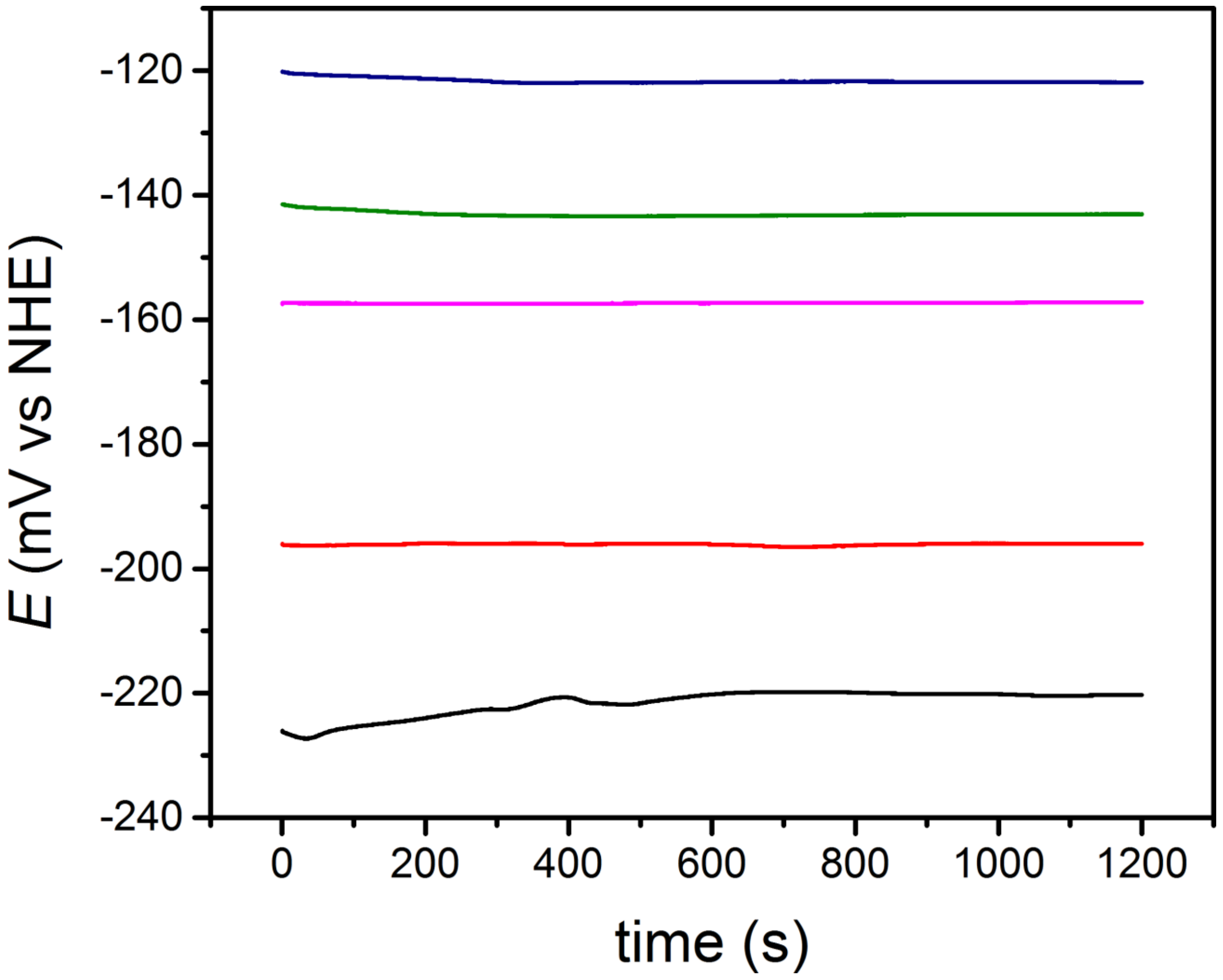

Figure S13. OCPs of solutions containing overall 3.8 mM Fe_2, with the ratio of **1**:**2** ranging from 9:1 (bottom most) to 1:9 (top most), are monitored over time upon mixing (at 0 s). Each solution contains 100 mM NaCl and 100 mM HEPES buffered at pH 7.5.

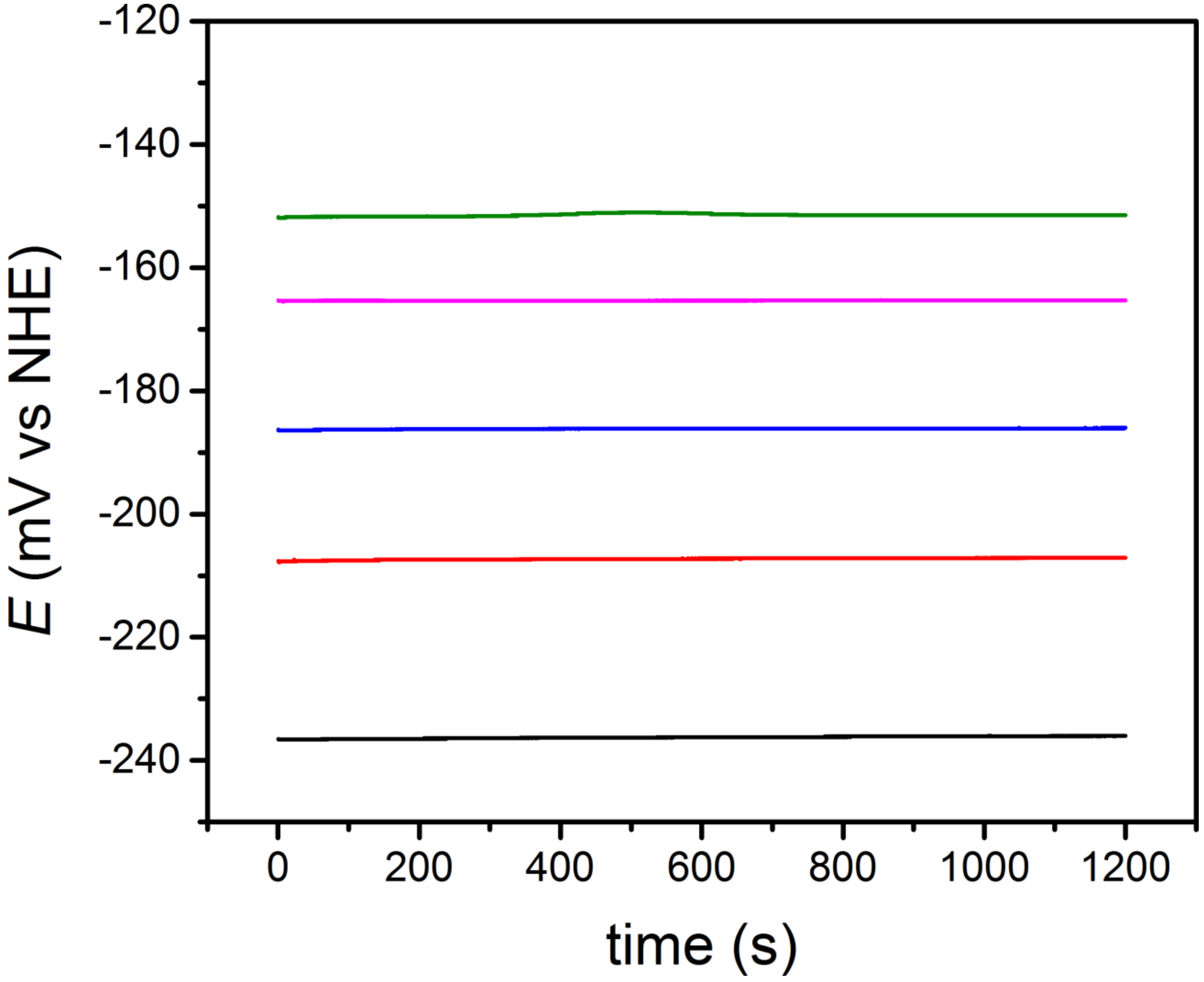

Figure S14. OCPs of solutions containing overall 2.6 mM Fe_2, with the ratio of **1**:**2** ranging from 9:1 (bottom most) to 1:9 (top most), are monitored over time upon mixing (at 0 s). Each solution contains 100 mM NaCl and 100 mM HEPES buffered at pH 7.3.

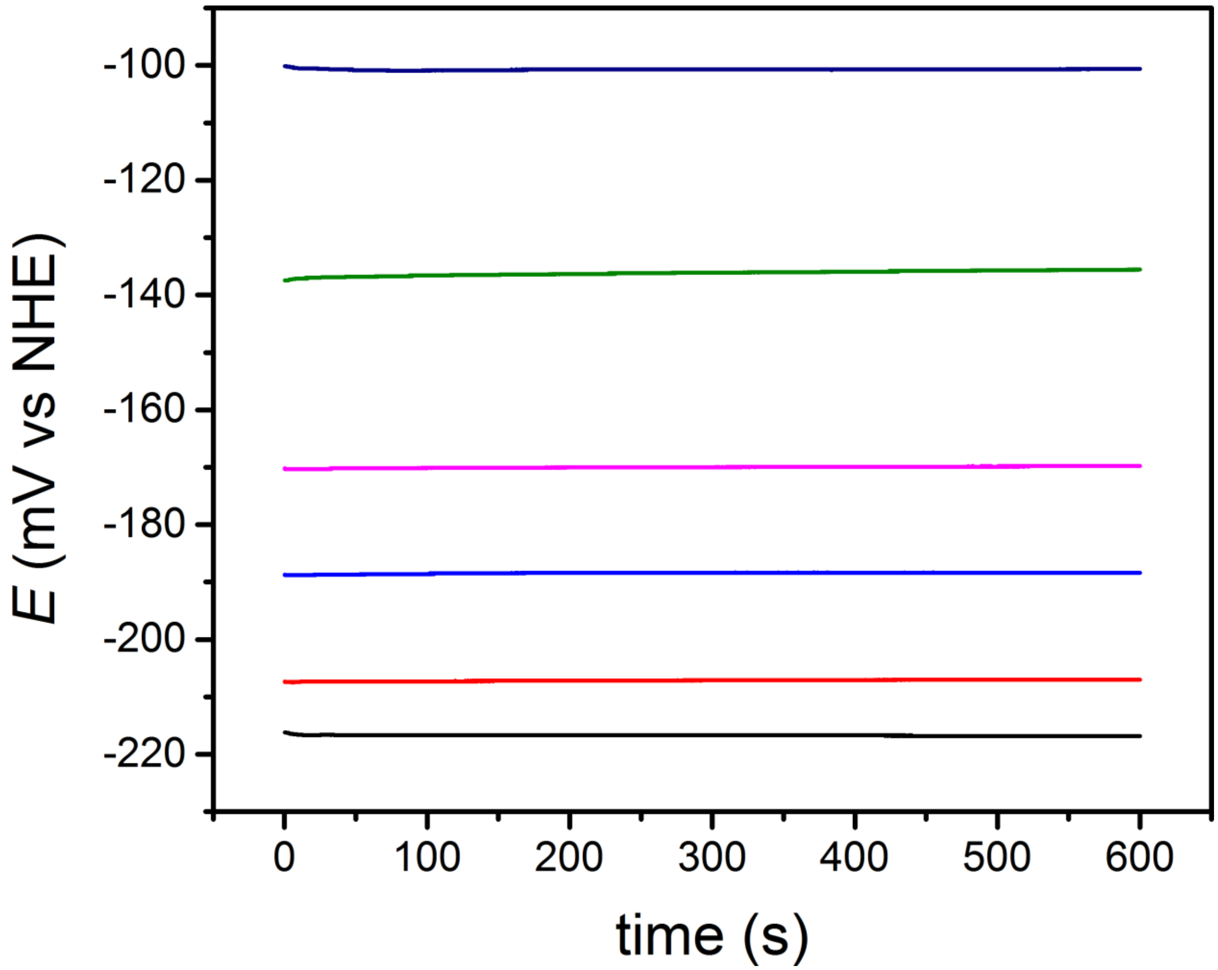

Figure S15. OCPs of solutions containing overall 10 mM Fe_2, with the ratio of **1**:**2** ranging from 9:1 (bottom most) to 1:9 (top most), are monitored over time upon mixing (at 0 s). Each solution contains 100 mM NaCl and 100 mM HEPES buffered at pH 7.4

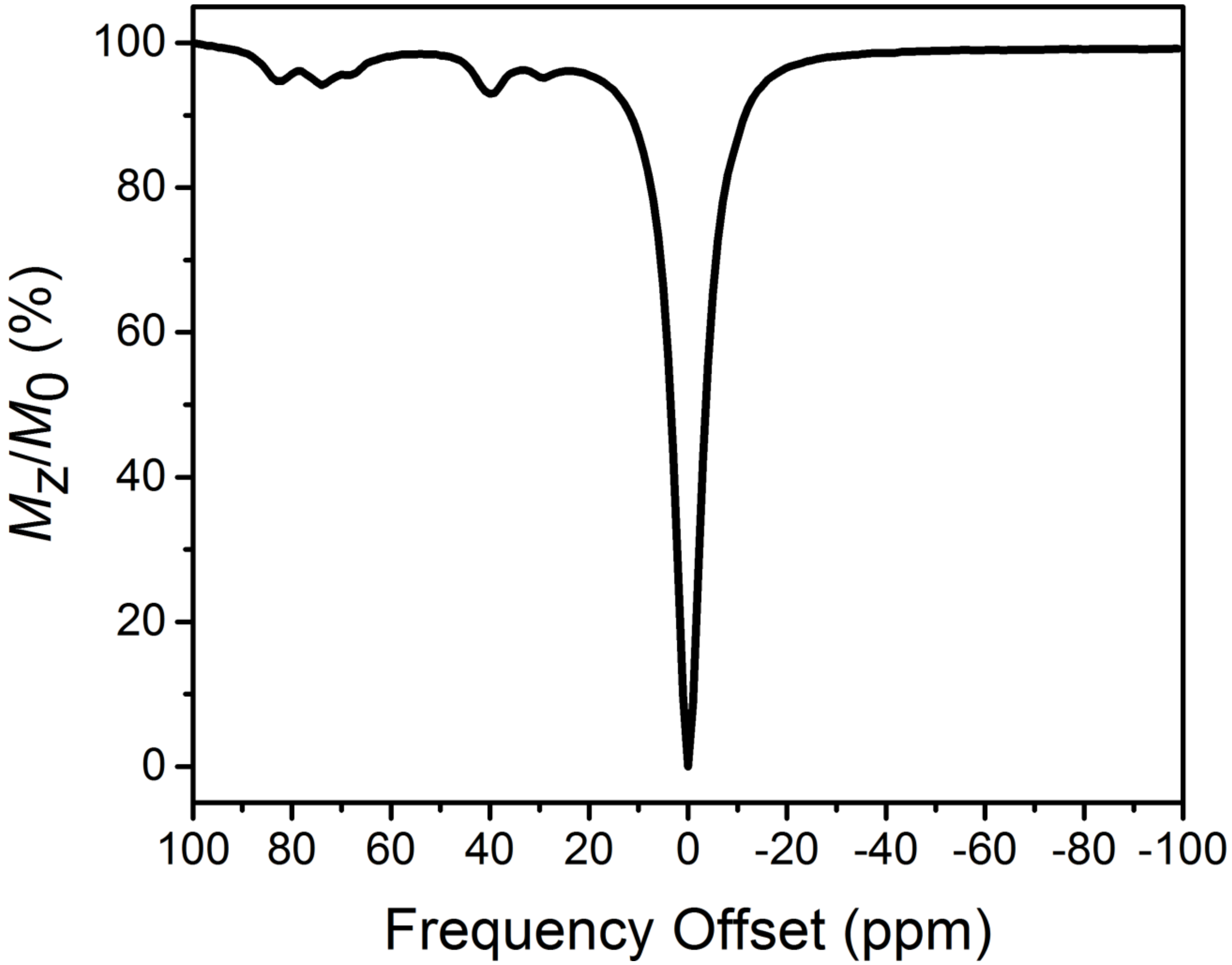

Figure S16. CEST spectra collected at 37 °C for a sample containing 4 mM of **1** mixed with 1 mM of KO_2 in a solution containing 100 mM HEPES and 100 mM NaCl buffered at pH 7.4. Refer to the CEST experimental section for details on baseline correction.

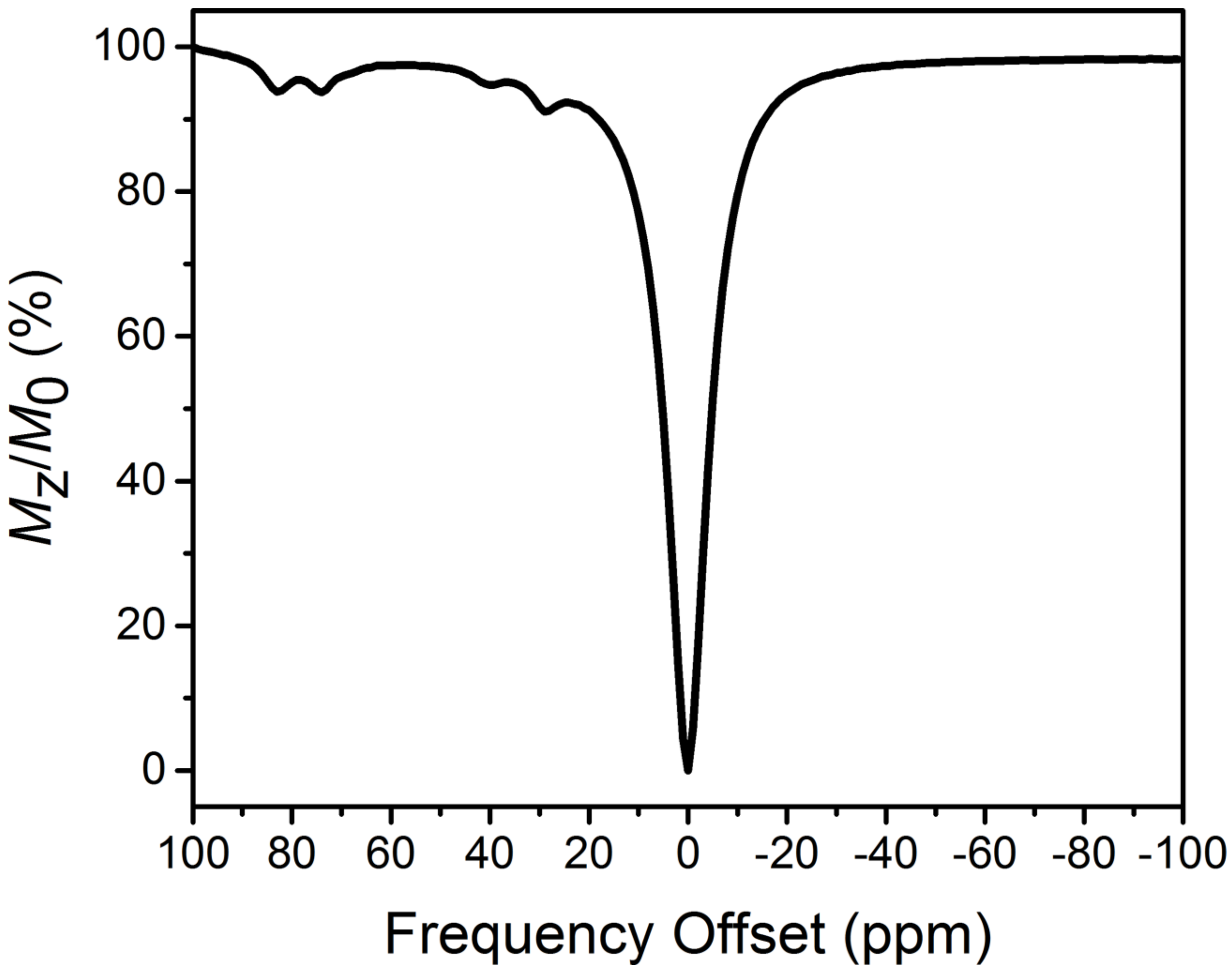

Figure S17. CEST spectra collected at 37 °C for a sample containing 4 mM of **2** mixed with 200 mM of cysteine in a solution containing 100 mM HEPES and 100 mM NaCl buffered at pH 7.4. Refer to the CEST experimental section for details on baseline correction.

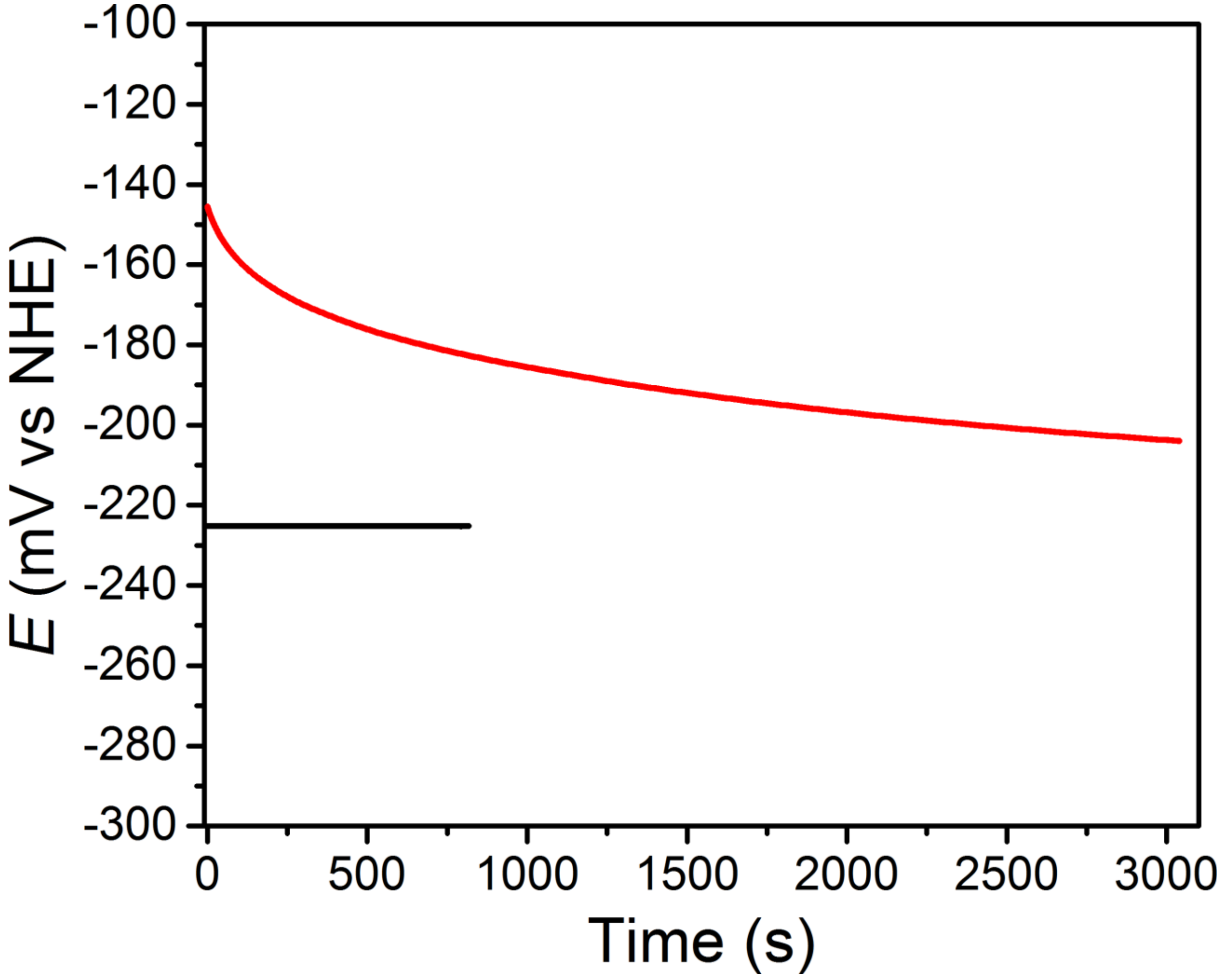

Figure S18. OCPs of two solutions containing 4 mM **1** with 1 mM KO_2 (black) and 4mM **2** with 200 mM cysteine (red) are monitored over time upon mixing (at 0 s). Measurements were stopped when OCP varied less than 1 mV within 5 minutes. Each solution contains 100 mM NaCl and 100 mM HEPES buffered at pH 7.4.

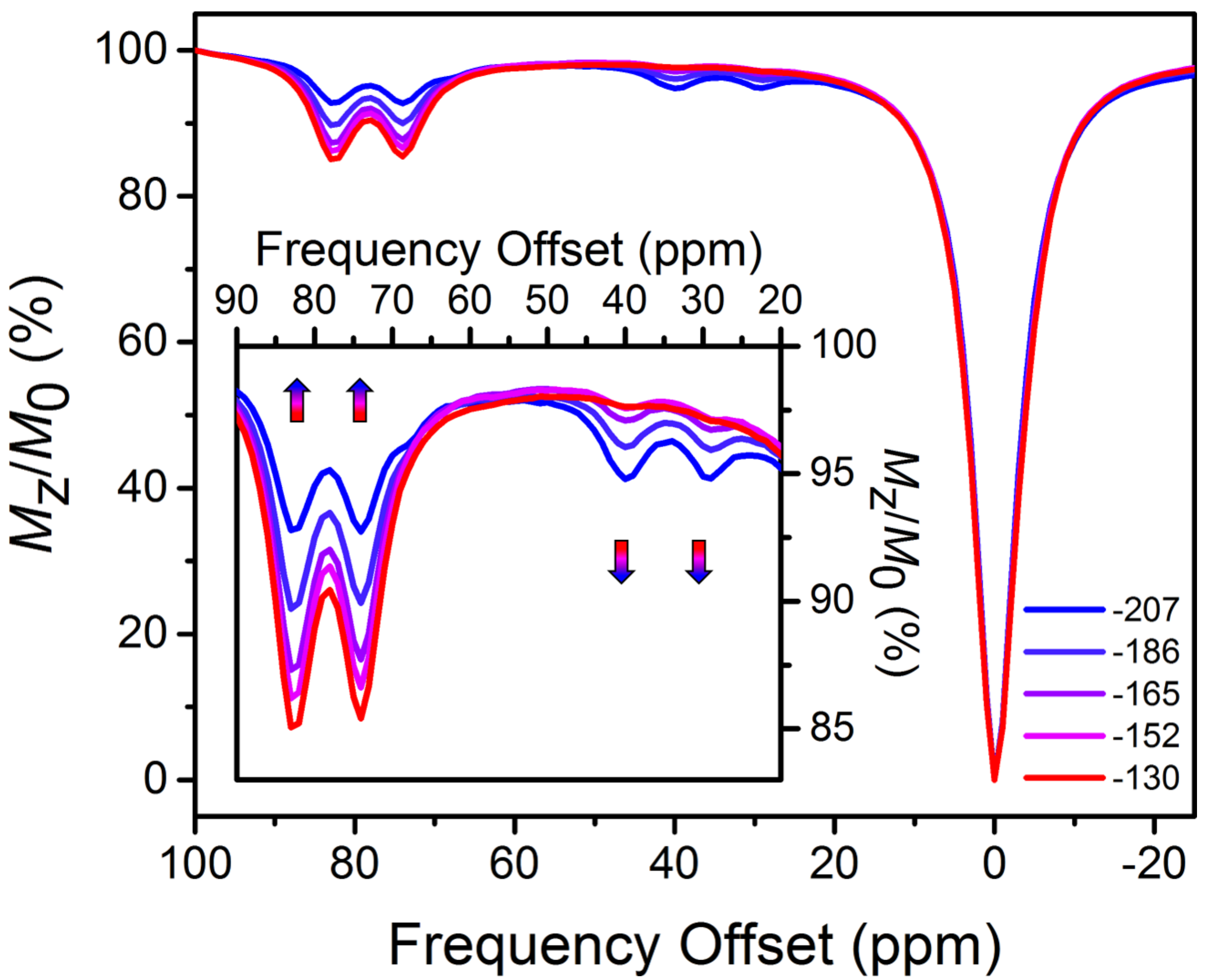

Figure S19. CEST spectra for 2.6 mM aqueous solutions of **1** and **2**, with ratios of **1**:**2** from 9:1 (blue) to 1:9 (red) at 37 °C. Each solution contains 100 mM NaCl and 100 mM HEPES buffered at pH 7.3. The legend gives the independently obtained OCP of each sample (mV vs NHE). Inset: Expanded view of the relevant CEST peaks.

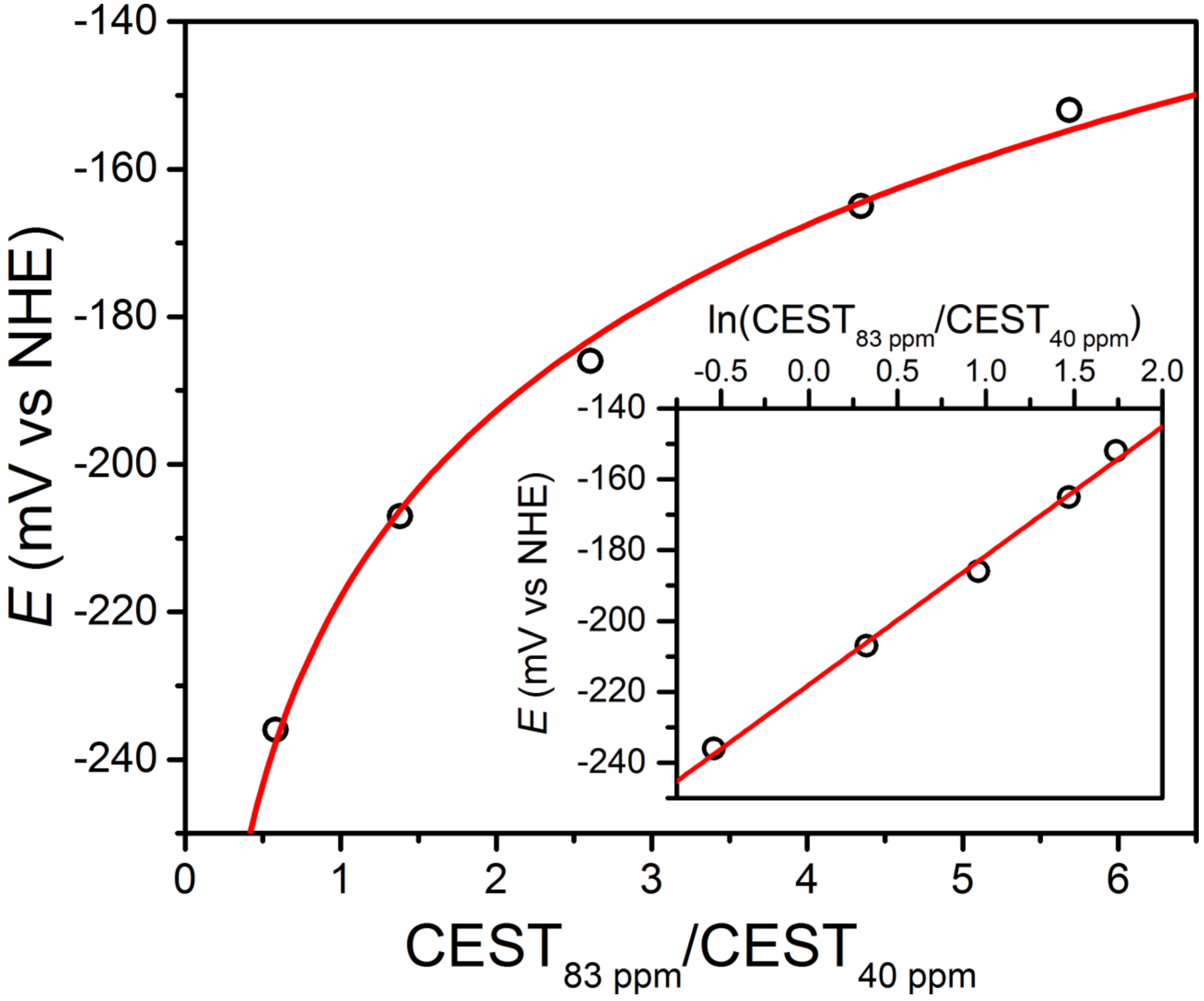

Figure S20. Open circuit potentials for solutions, containing 100 mM HEPES, 100 mM NaCl and 2.6 mM Fe_2 buffered at pH 7.3 at 37 °C, is plotted against both the ratio of CEST effects from application of presaturation at 83 and 40 ppm and the natural log of the ratio (inset). Black circles and the red line represent the experimental data and the fit, respectively.

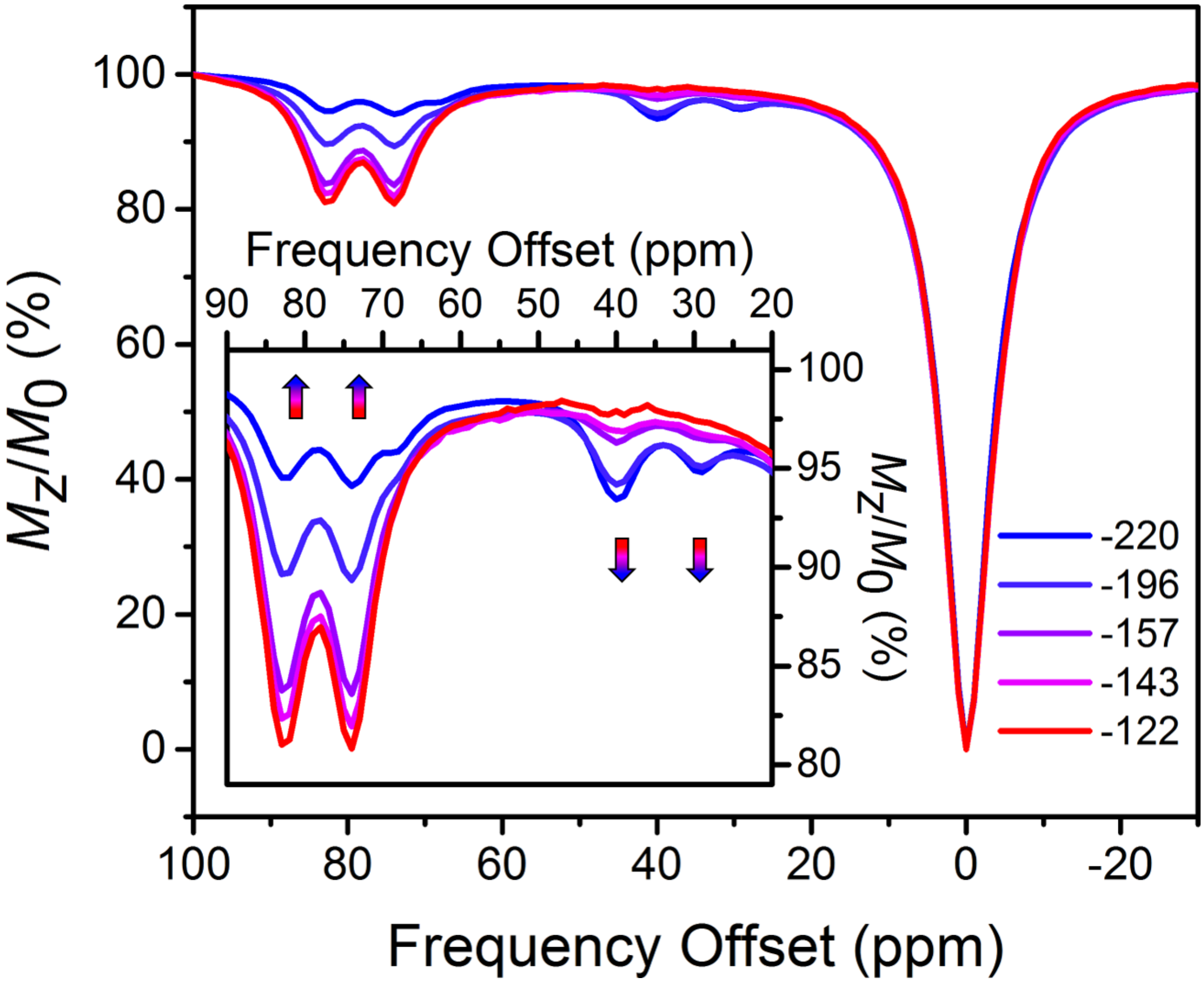

Figure S21. CEST spectra for 3.8 mM aqueous solutions of **1** and **2**, with ratios of **1**:**2** from 9:1 (blue) to 1:9 (red) at 37 °C. Each solution contains 100 mM NaCl and 100 mM HEPES buffered at pH 7.5. The legend gives the independently obtained OCP of each sample (mV vs NHE). Inset: Expanded view of the relevant CEST peaks.

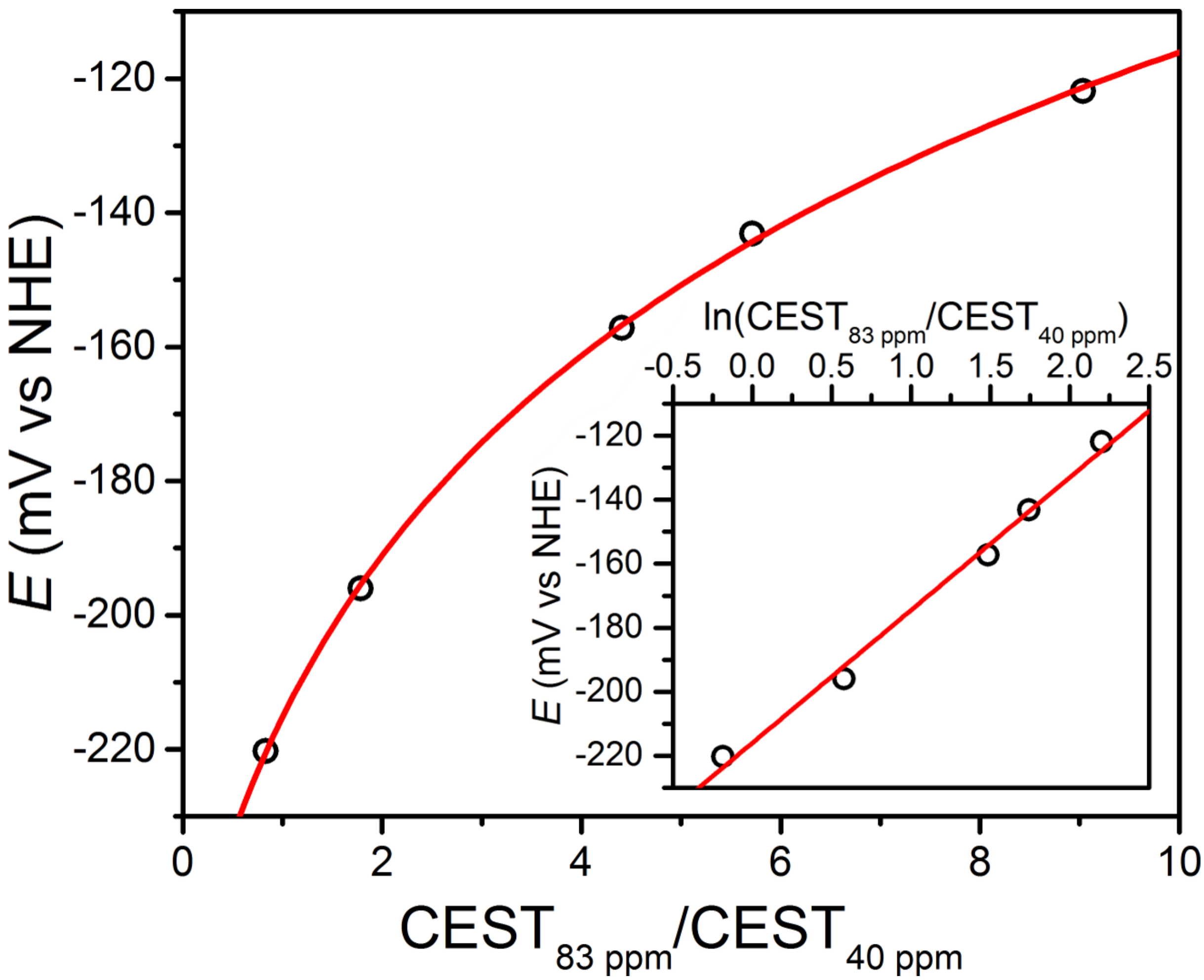

Figure S22. Open circuit potentials for solutions, containing 100 mM HEPES, 100 mM NaCl and 3.8 mM Fe_2 buffered at pH 7.5 at 37 °C, is plotted against both the ratio of CEST effects from application of presaturation at 83 and 40 ppm and the natural log of the ratio (inset). Black circles and the red line represent the experimental data and the fit, respectively.

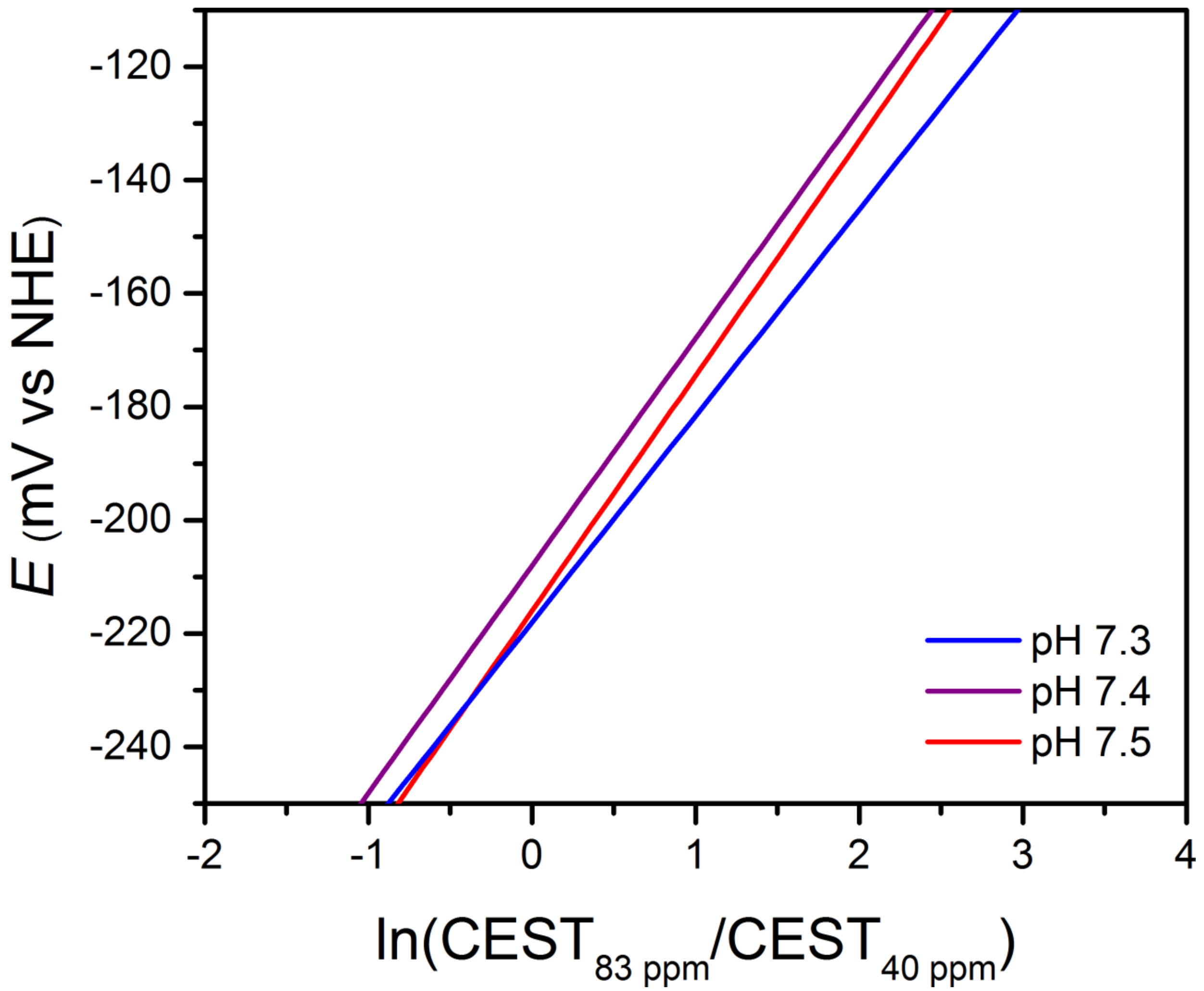

Figure S23. Comparison of Nernstian fits (from Figures 4, S20, and S22) obtained from data at various pH values.

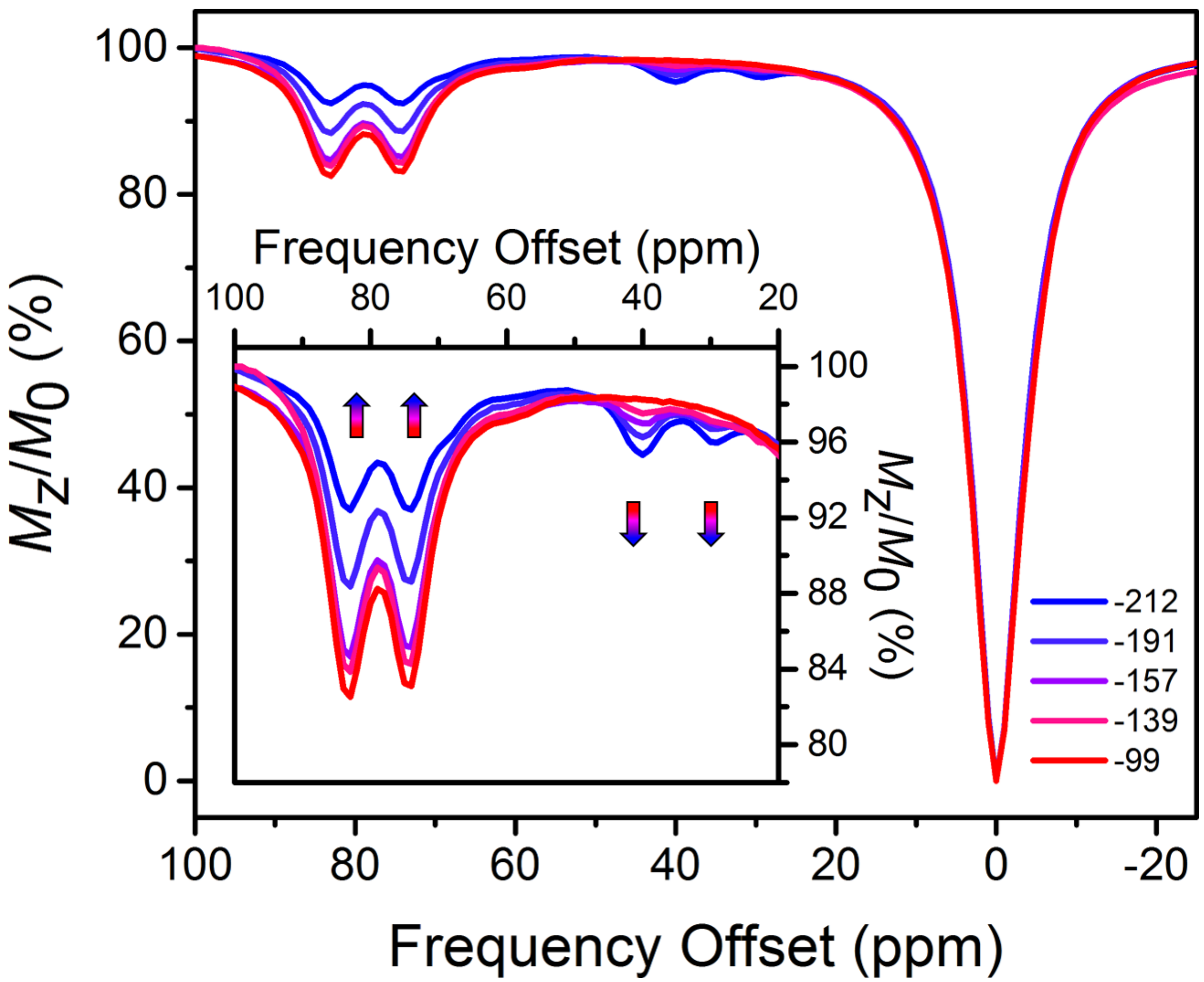

Figure S24. CEST spectra for 3.8 mM aqueous solutions of **1** and **2**, with ratios of **1**:**2** from 9:1 (blue) to 1:9 (red) at 35 °C. Each solution contains 100 mM NaCl and 100 mM HEPES buffered at pH 7.5. The legend gives the independently obtained OCP of each sample (mV vs NHE). Inset: Expanded view of the relevant CEST peaks.

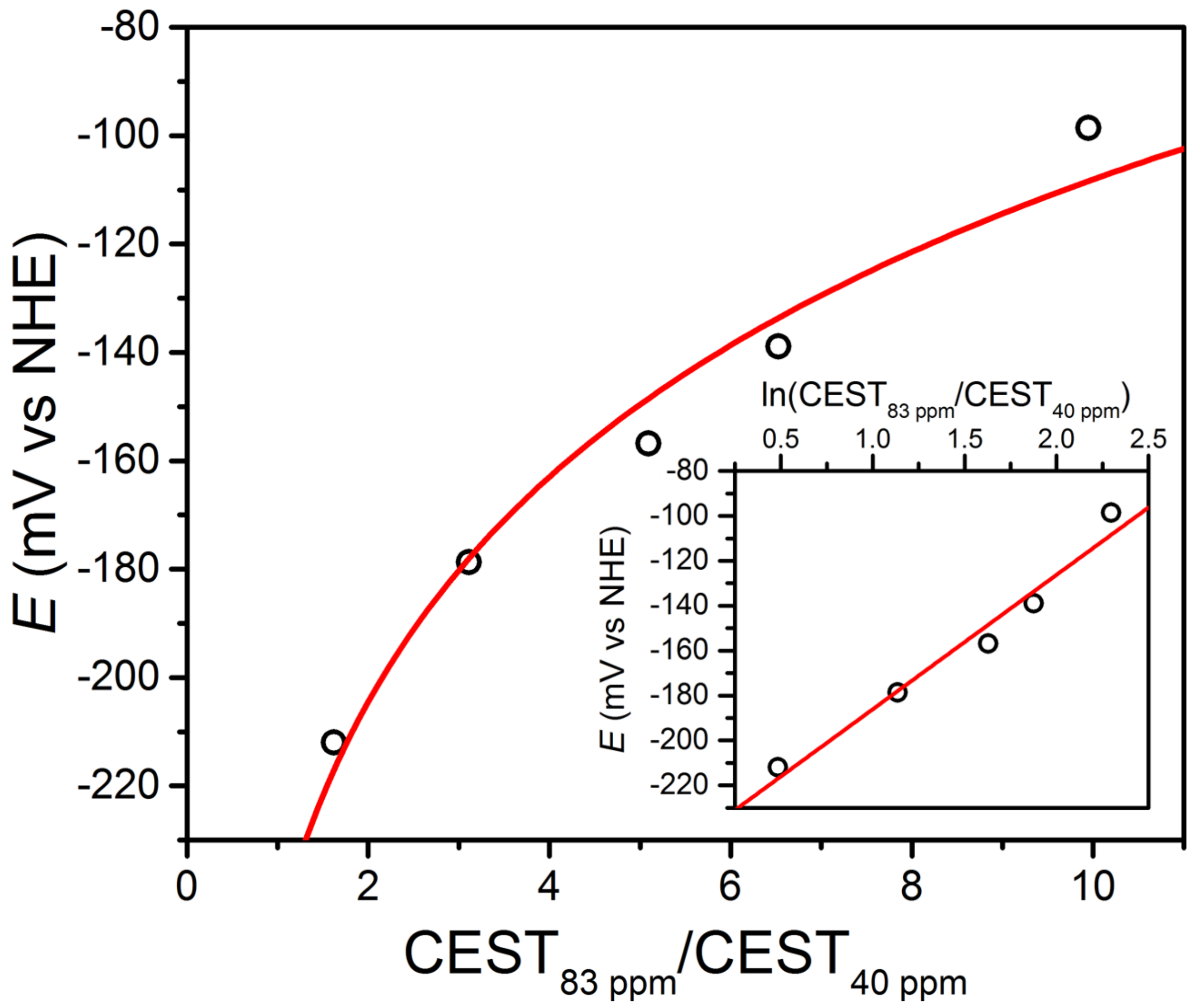

Figure S25. Open circuit potentials for solutions, containing 100 mM HEPES, 100 mM NaCl and 3.8 mM Fe_2 buffered at pH 7.5 at 35 °C, is plotted against both the ratio of CEST effects from application of presaturation at 83 and 40 ppm and the natural log of the ratio (inset). Black circles and the red line represent the experimental data and the fit, respectively.

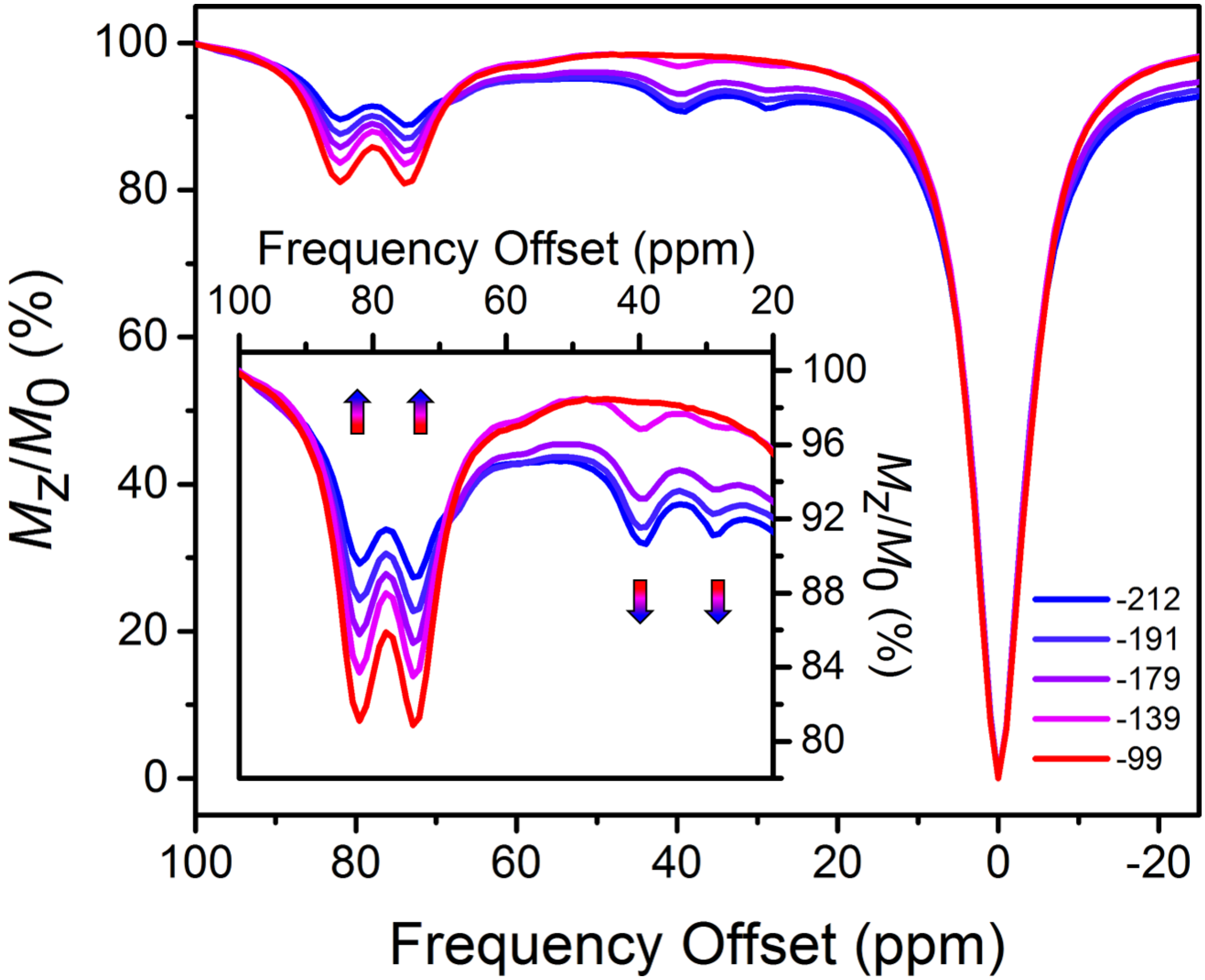

Figure S26. CEST spectra for 3.8 mM aqueous solutions of **1** and **2**, with ratios of **1**:**2** from 9:1 (blue) to 1:9 (red) at 39 °C. Each solution contains 100 mM NaCl and 100 mM HEPES buffered at pH 7.5. The legend gives the independently obtained OCP of each sample (mV vs NHE). Inset: Expanded view of the relevant CEST peaks.

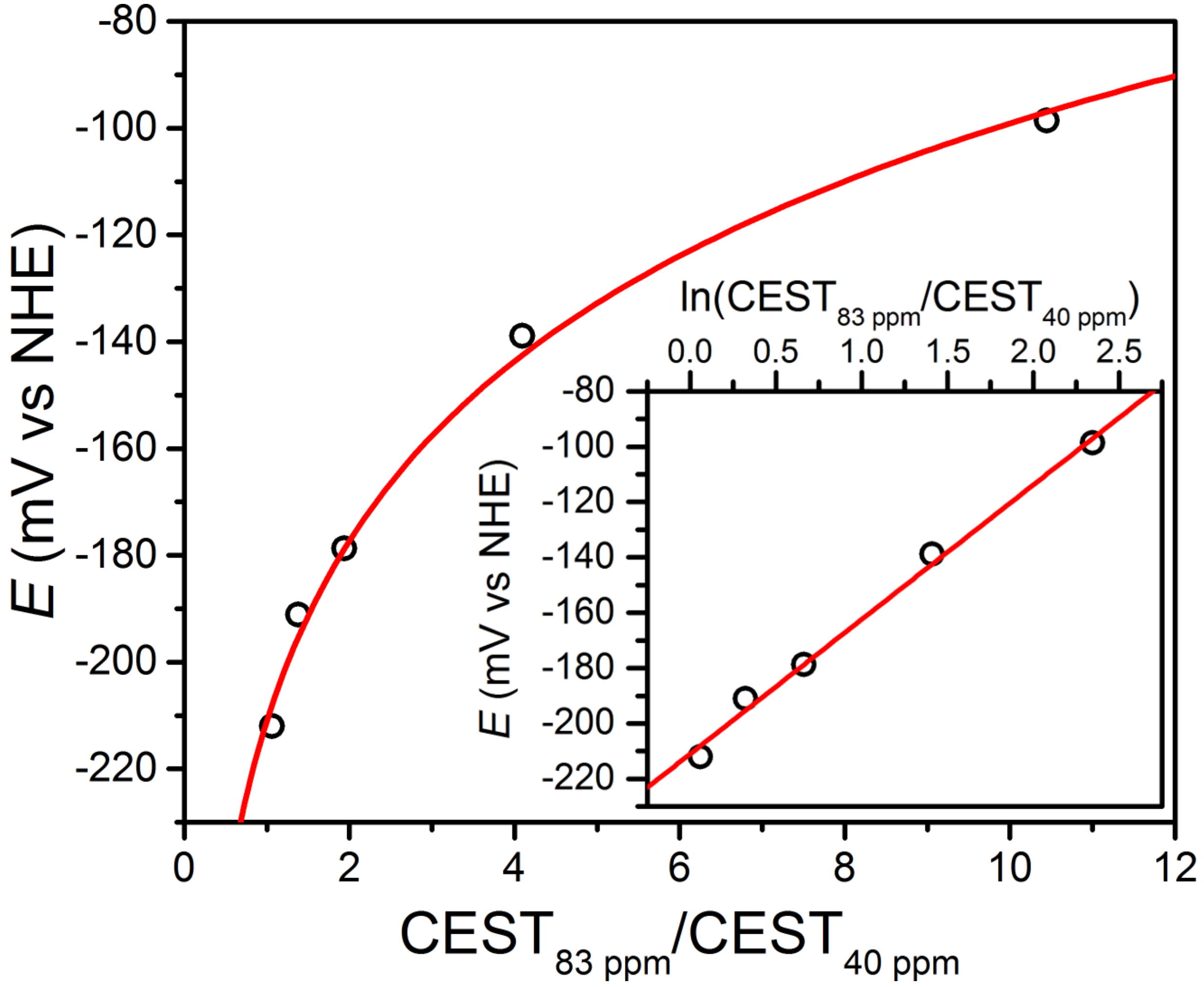

Figure S27. Open circuit potentials for solutions, containing 100 mM HEPES, 100 mM NaCl and 3.8 mM Fe_2 buffered at pH 7.5 at 39 °C, is plotted against both the ratio of CEST effects from application of presaturation at 83 and 40 ppm and the natural log of the ratio (inset). Black circles and the red line represent the experimental data and the fit, respectively.

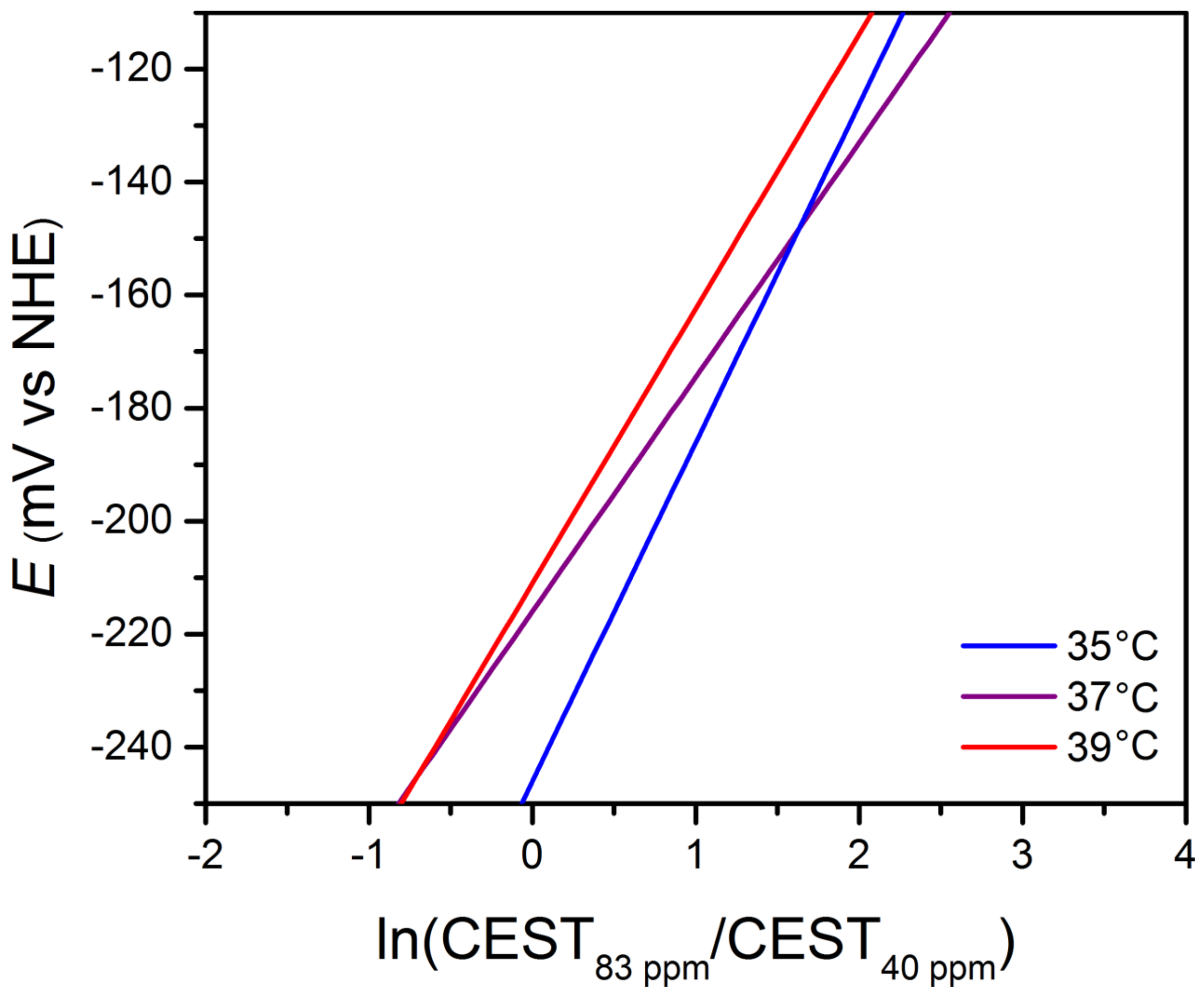

Figure S28. Comparison of Nernstian fits (from Figures S22, S25 and S27) obtained from data at various temperatures.

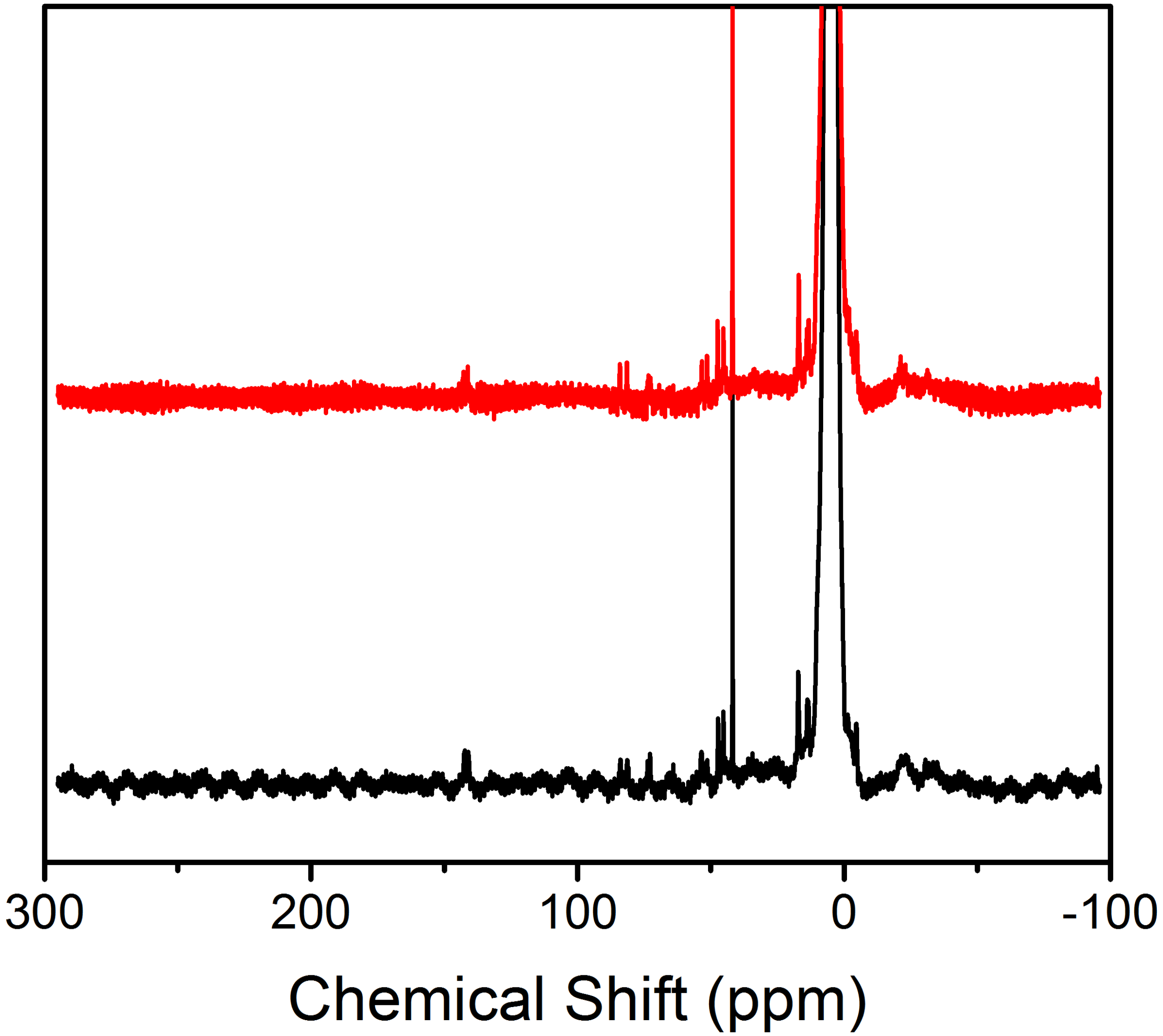

Figure S29. NMR spectra of 4 mM of **1** in pH 7.4 buffer with (top) and without (bottom) presences of 4 mM of each NaOAc, Na_2CO_3, NaH_2PO_4 and Na_2SO_4.

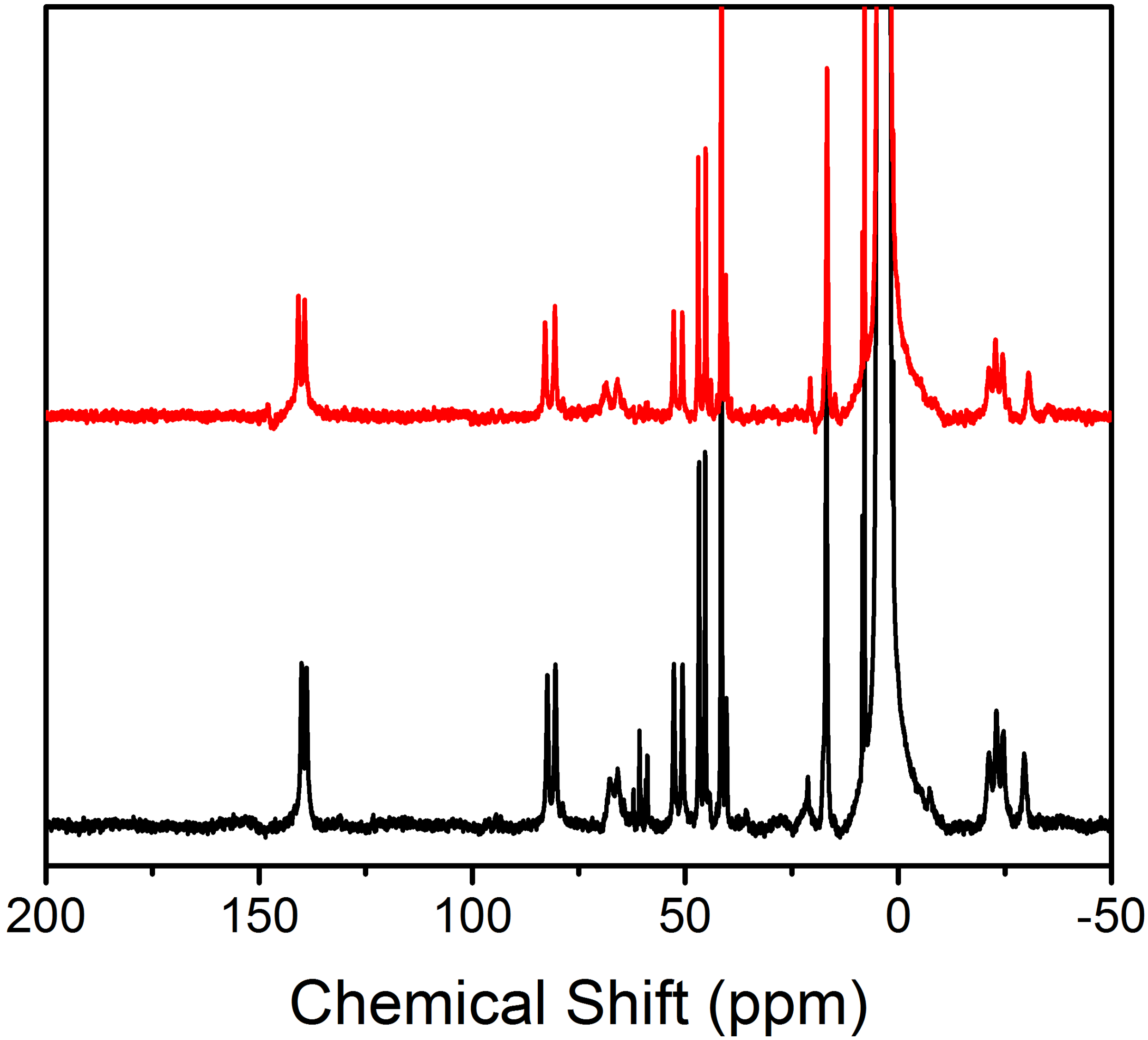

Figure S30. NMR spectra of 4 mM of **1** in D_2O with (top) and without (bottom) presences of 4 mM of each NaOAc, Na_2CO_3, NaH_2PO_4 and Na_2SO_4.

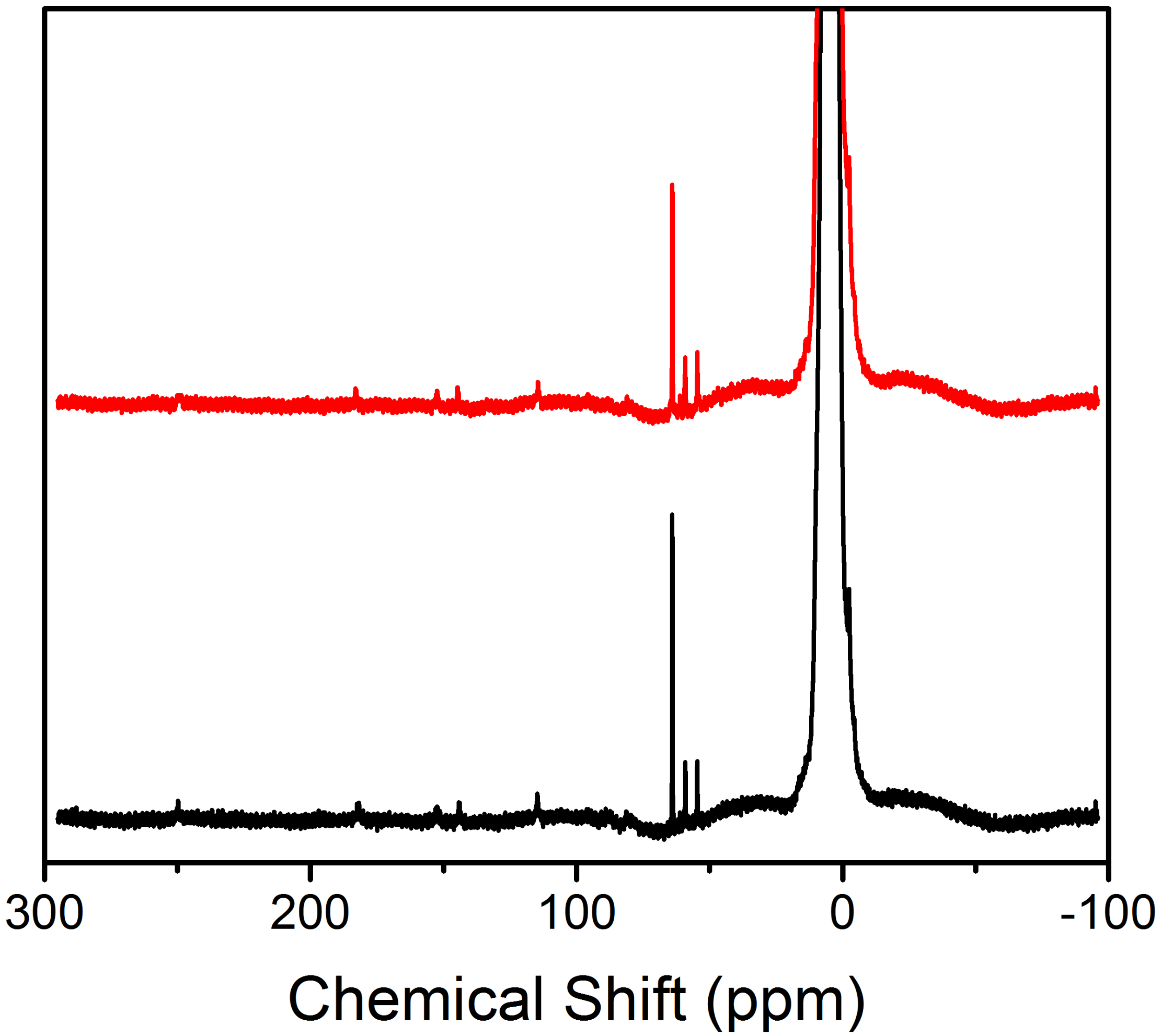

Figure S31. NMR spectra of 4 mM of **2** in pH 7.4 buffer with (top) and without (bottom) presences of 4 mM of each NaOAc, Na_2CO_3, NaH_2PO_4 and Na_2SO_4.

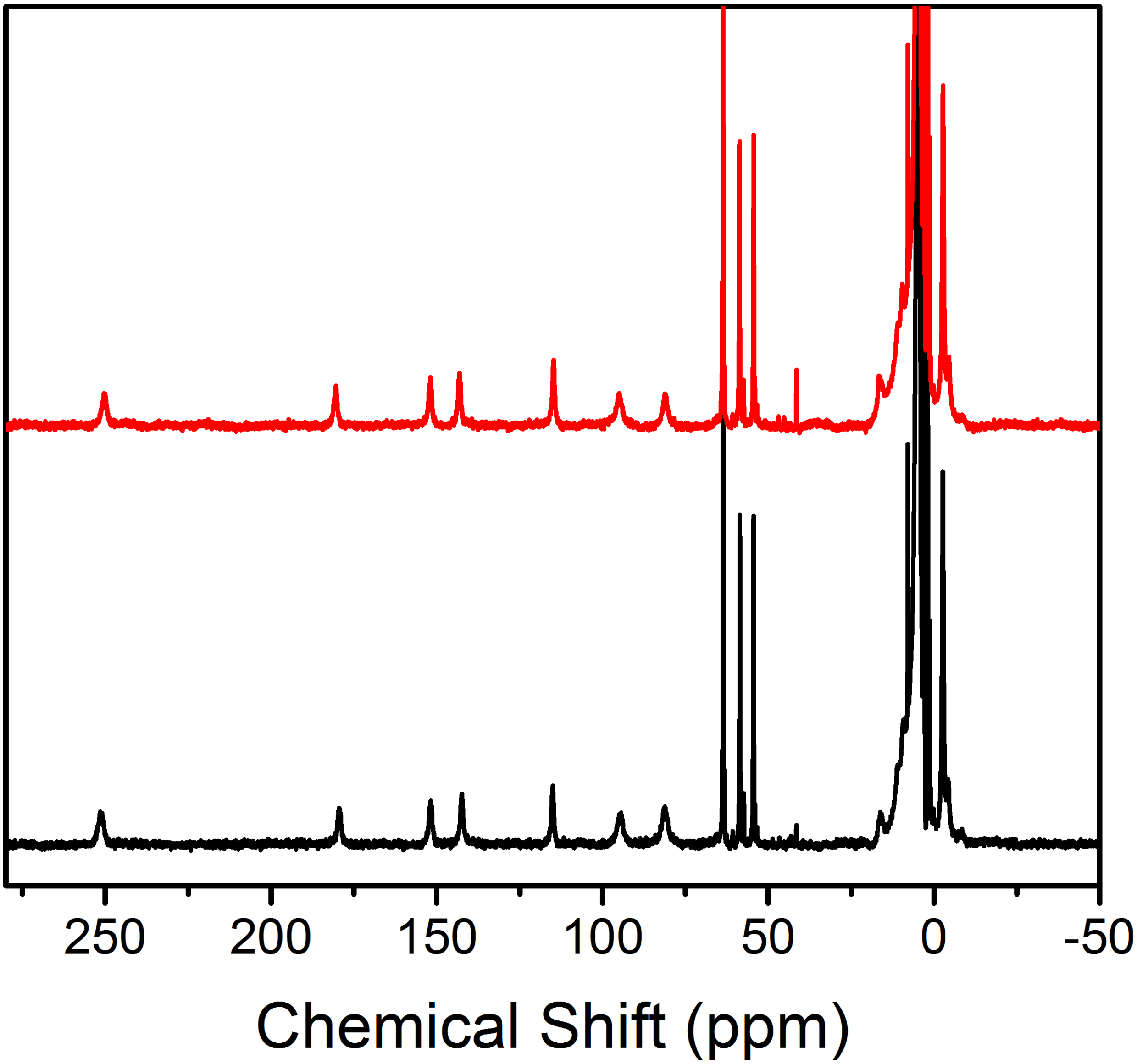

Figure S32. NMR spectra of 4 mM of **2** in D_2O with (top) and without (bottom) presences of 4 mM of each NaOAc, Na_2CO_3, NaH_2PO_4 and Na_2SO_4.

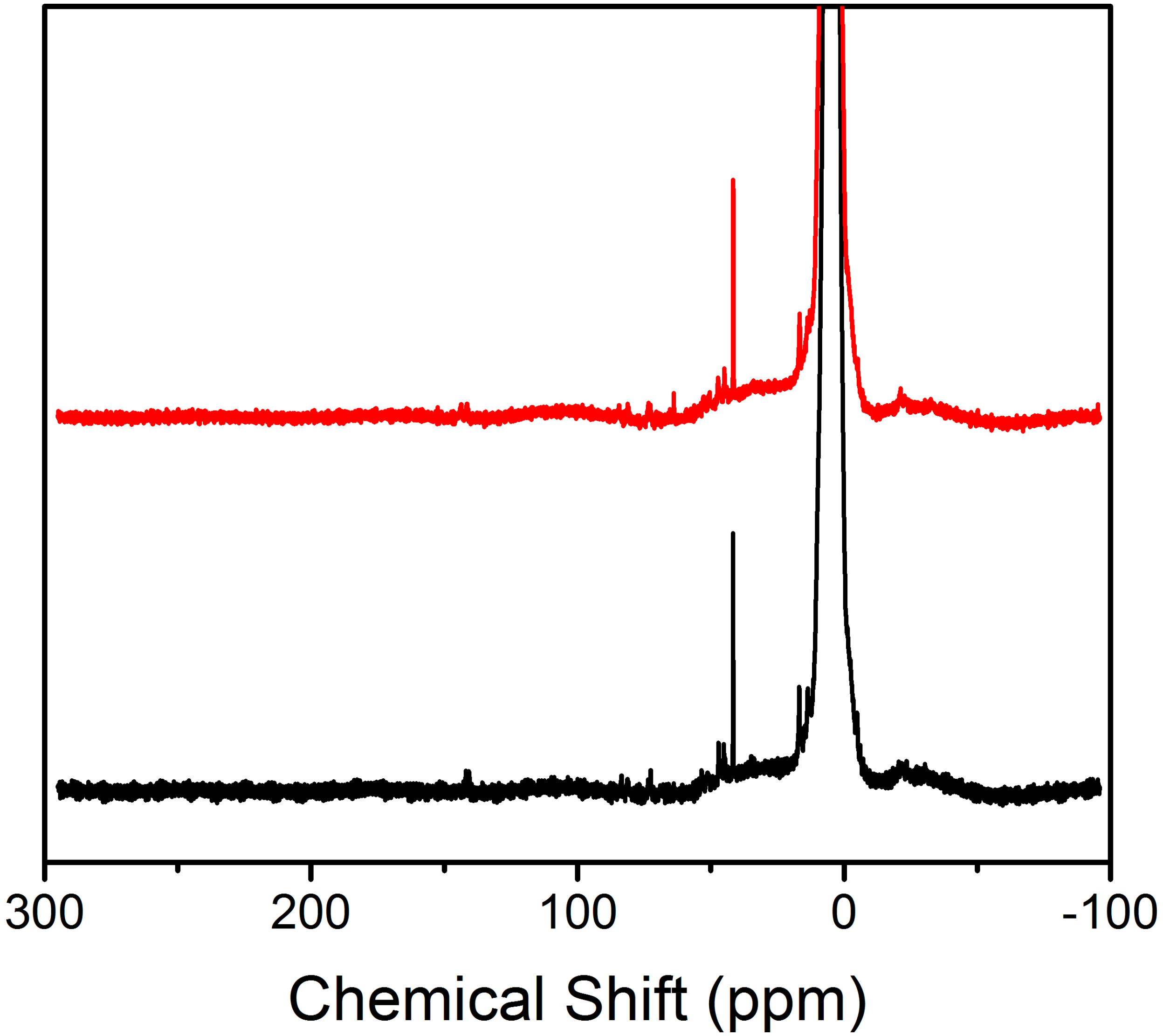

Figure S33. NMR spectra of 4 mM of **1** in pH 7.4 buffer with (top) and without (bottom) presences of 4 mM of $Ca(NO_3)_2$.

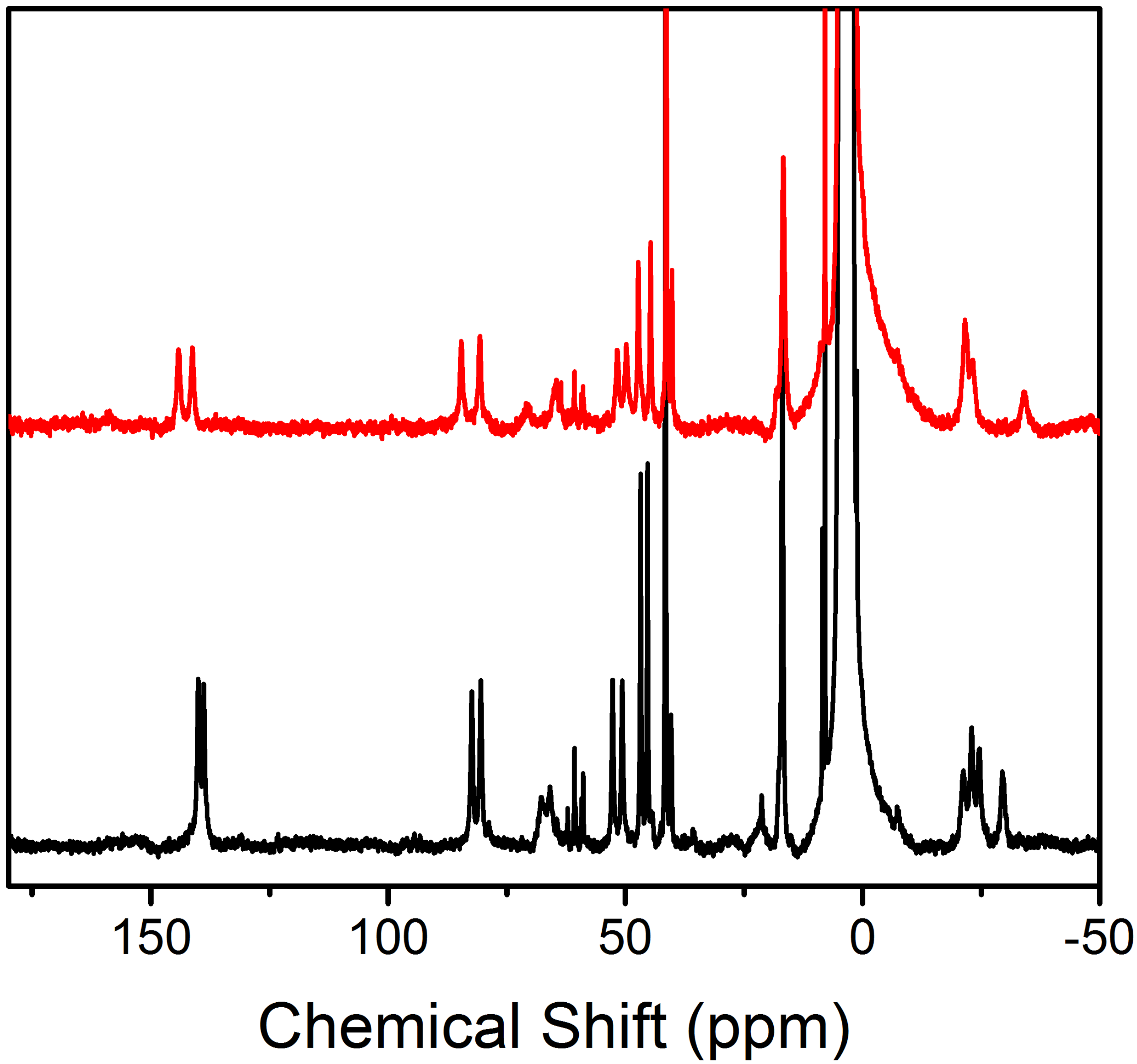

Figure S34. NMR spectra of 4 mM of **1** in D_2O with (top) and without (bottom) presences of 4 mM of $Ca(NO_3)_2$.

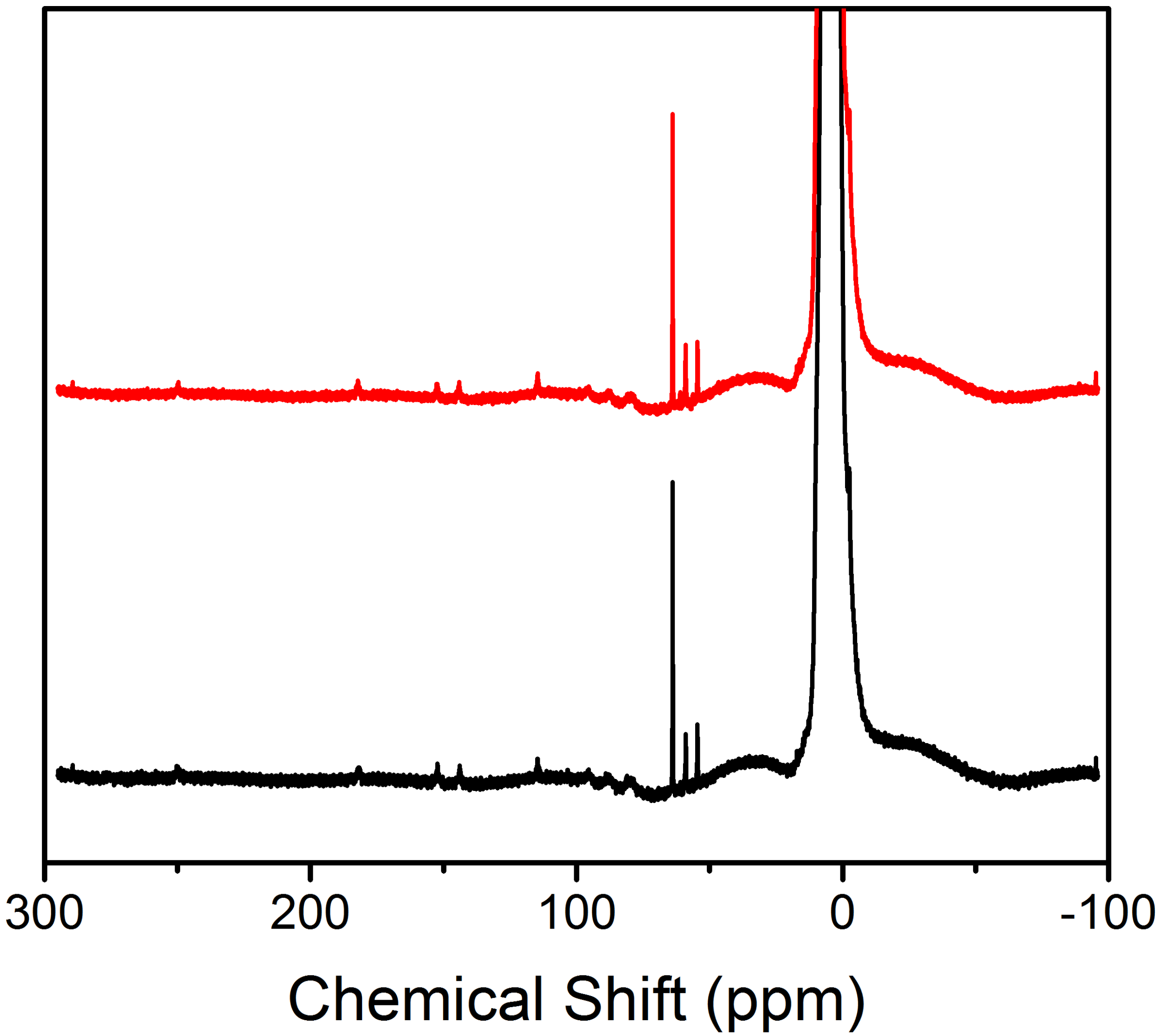

Figure S35. NMR spectra of 4 mM of **2** in pH 7.4 buffer with (top) and without (bottom) presences of 4 mM of $Ca(NO_3)_2$.

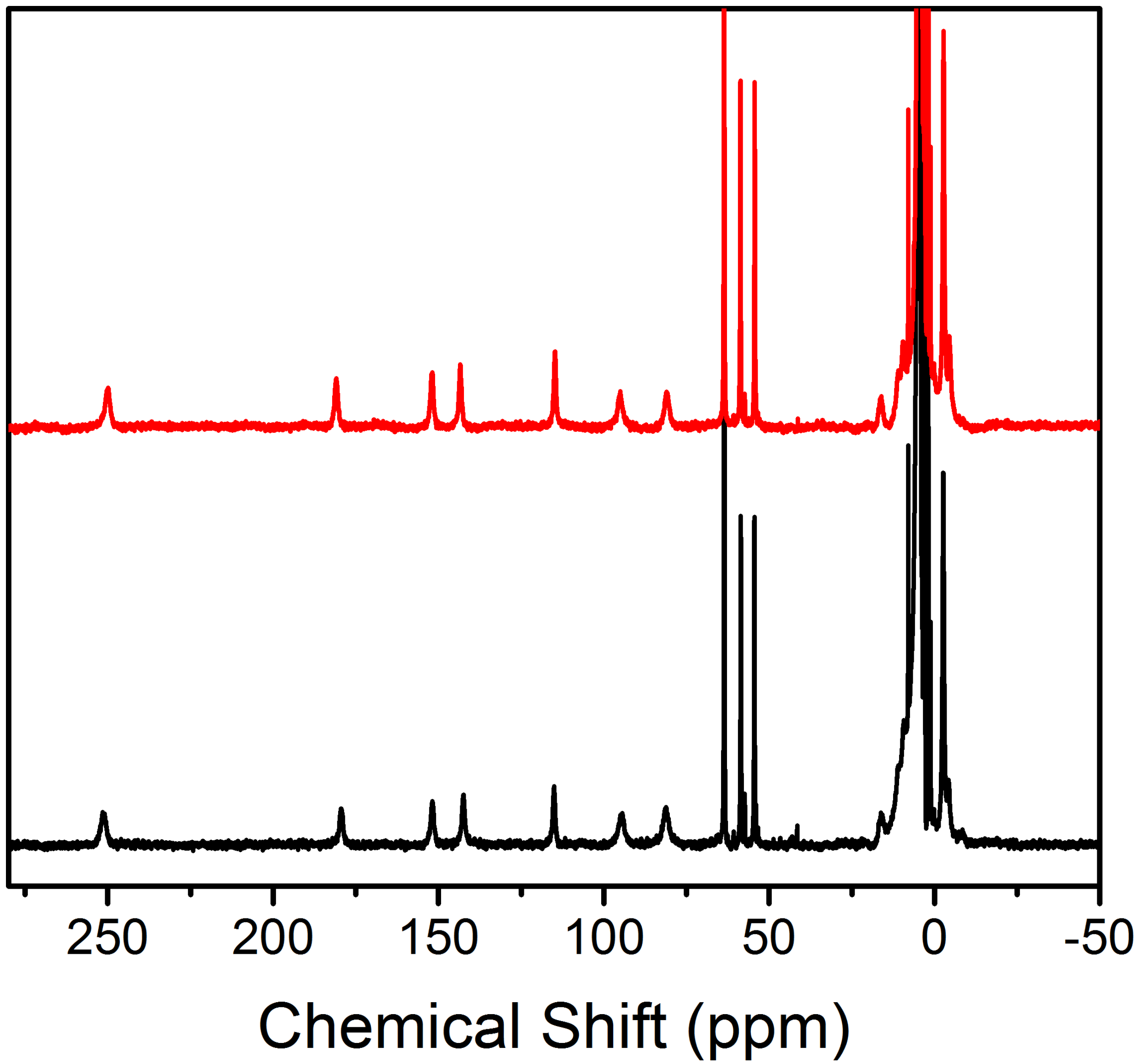

Figure S36. NMR spectra of 4 mM of **2** in D_2O with (top) and without (bottom) presences of 4 mM of $Ca(NO_3)_2$.

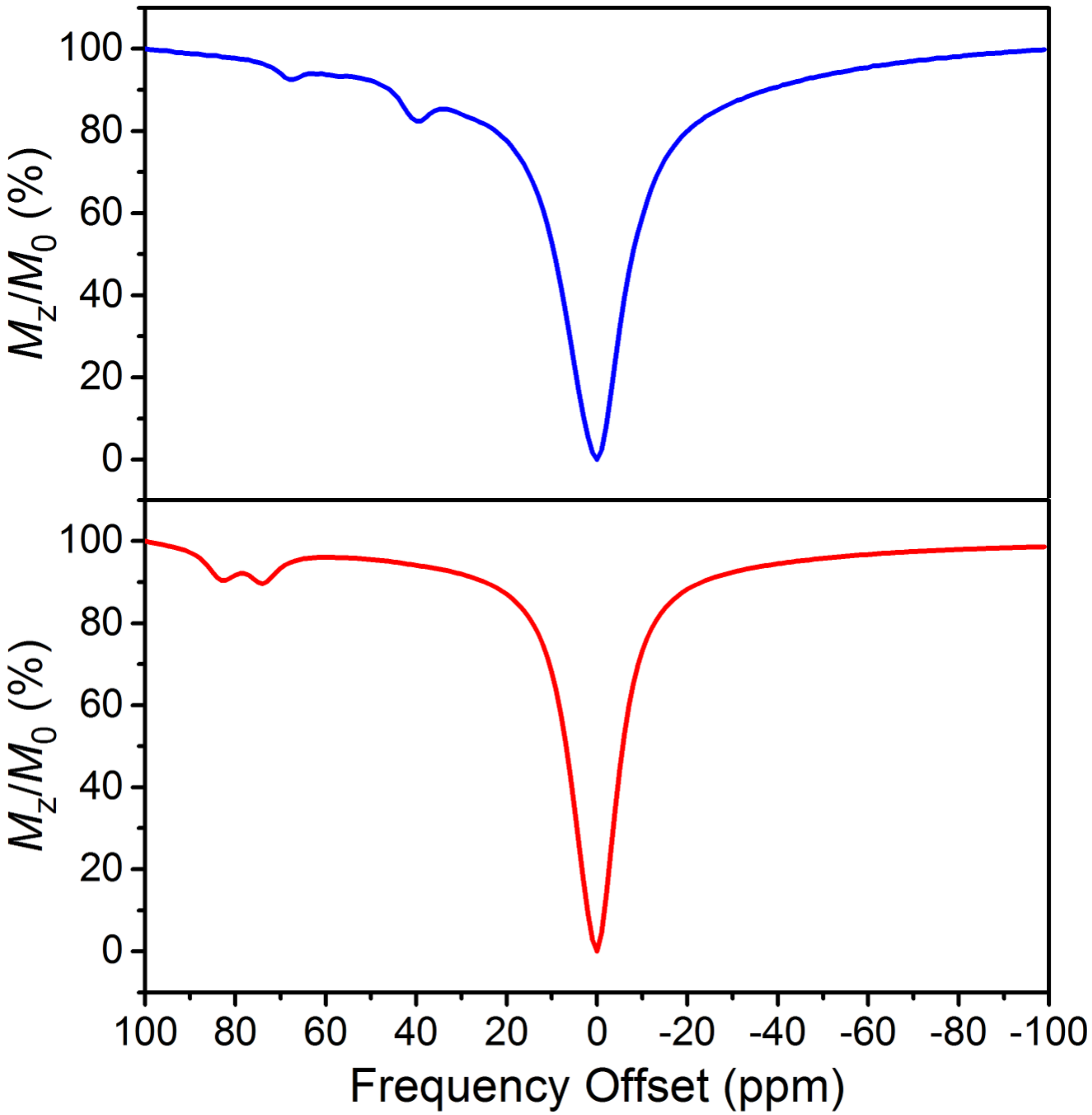

Figure S37. CEST spectra collected at 37 °C for solutions containing 4.0 mM of **1** (top) and **2** (bottom) in bovine blood plasma at pH 7.4.

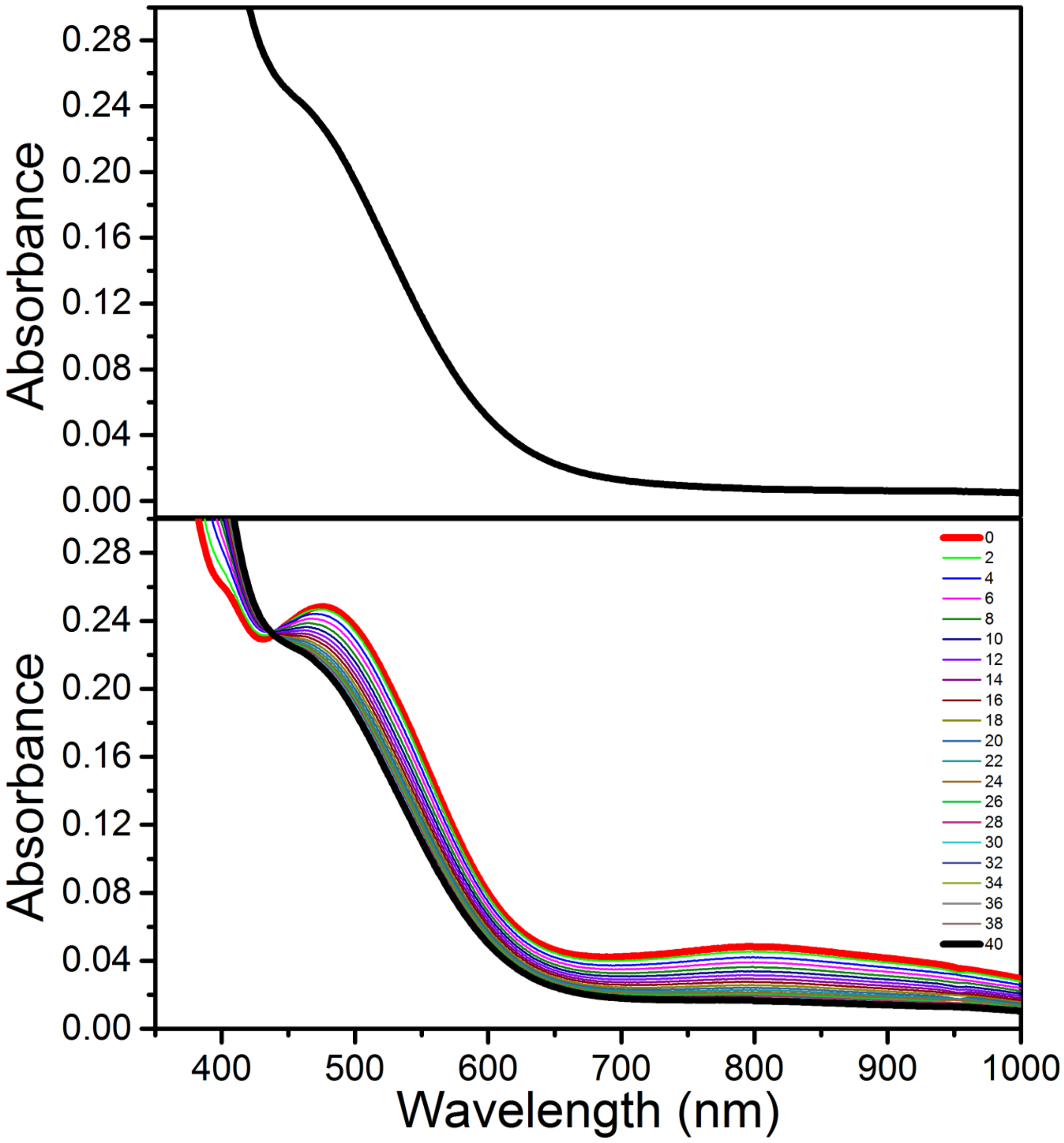

Figure S38. UV-Vis-NIR spectra of 0.4 mM of **3** (top) and 0.4 mM of **2**, buffered at pH 7.4 with different hours of air exposure (bottom, legend indicates hours of exposure).

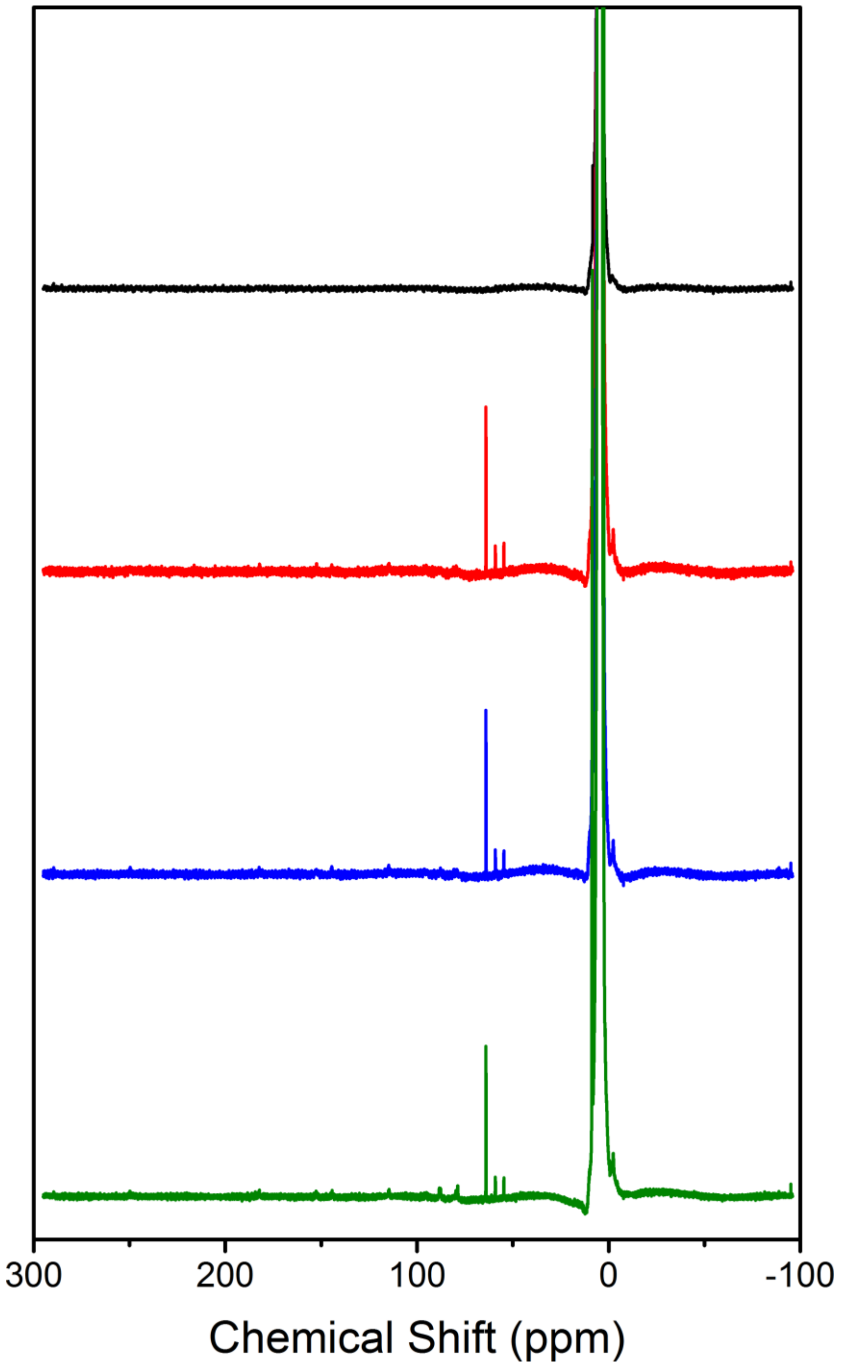

Figure S39. Stacked NMR spectra for 4 mM of **3** in solutions with 100 mM NaCl and 100 mM HEPES buffered at pH 7.4 in the presence of 0 (black), 4 (red), 8 (blue) and 16 (green) mM of glutathione.

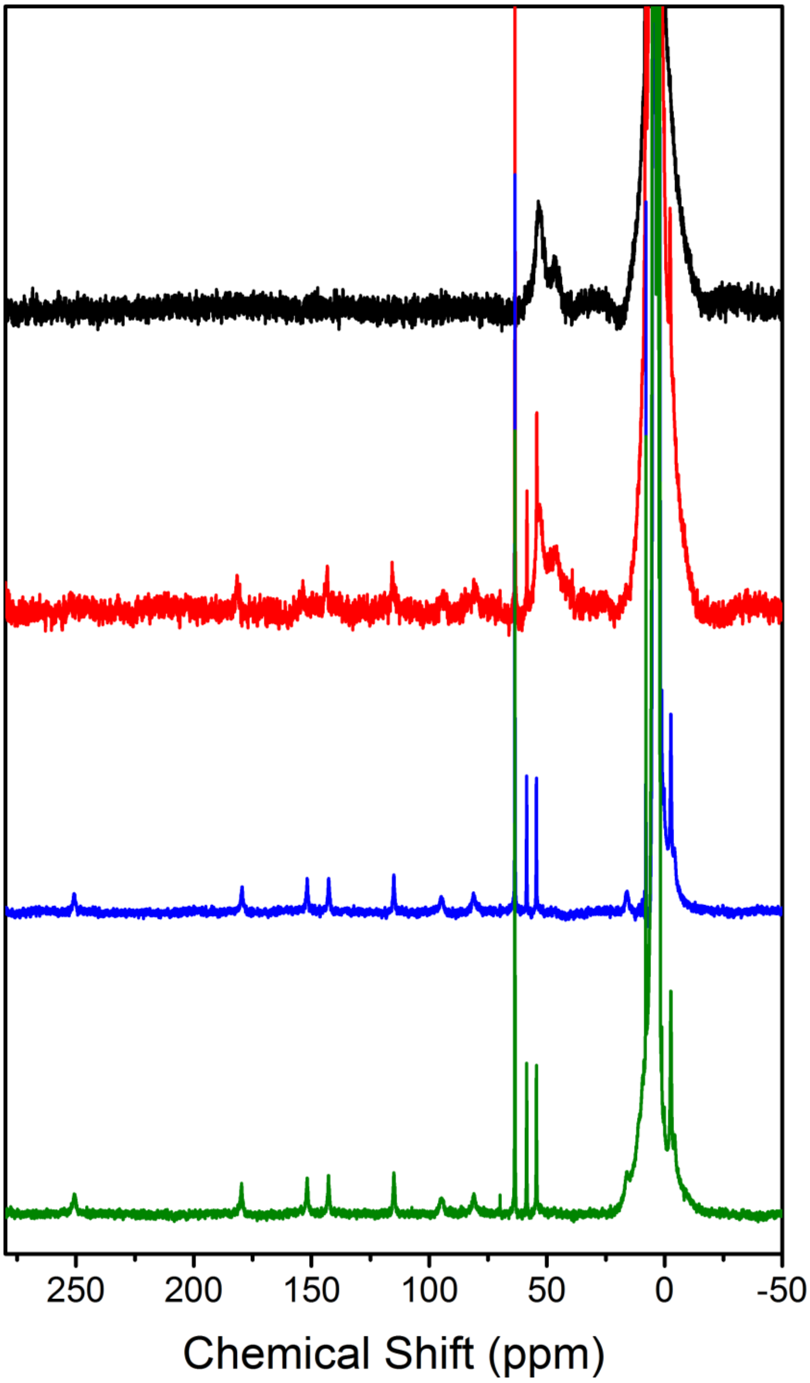

Figure S40. Stacked NMR spectra for 4 mM of **3** in D_2O in the presence of 0 (black), 4 (red), 8 (blue) and 16 (green) mM of glutathione monosodium salt, which is used to avoid acidity build-up caused by glutathione in an unbuffered solution.

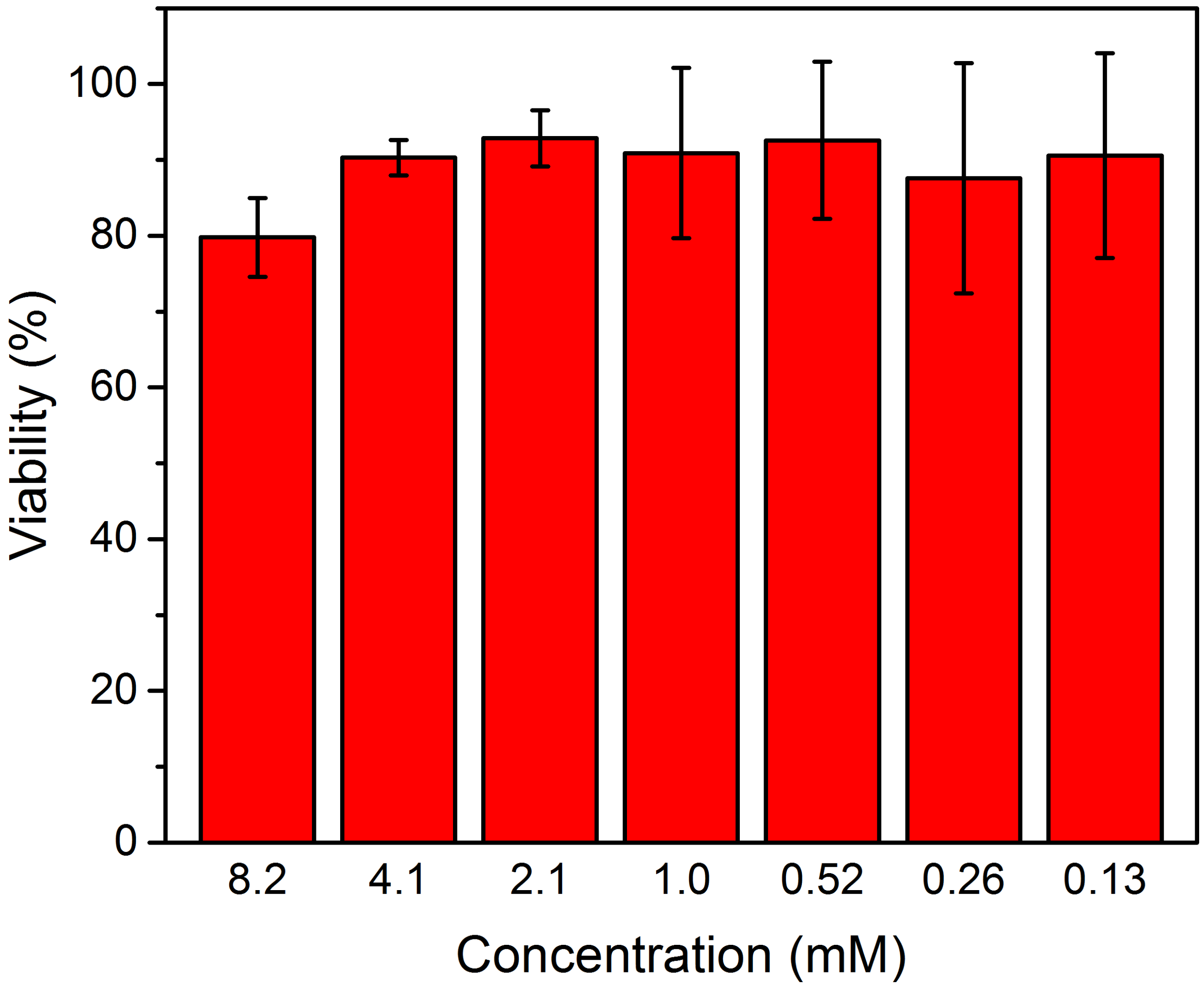

Figure S41. Cells survival (in %) after incubation with different concentrations of **3**.

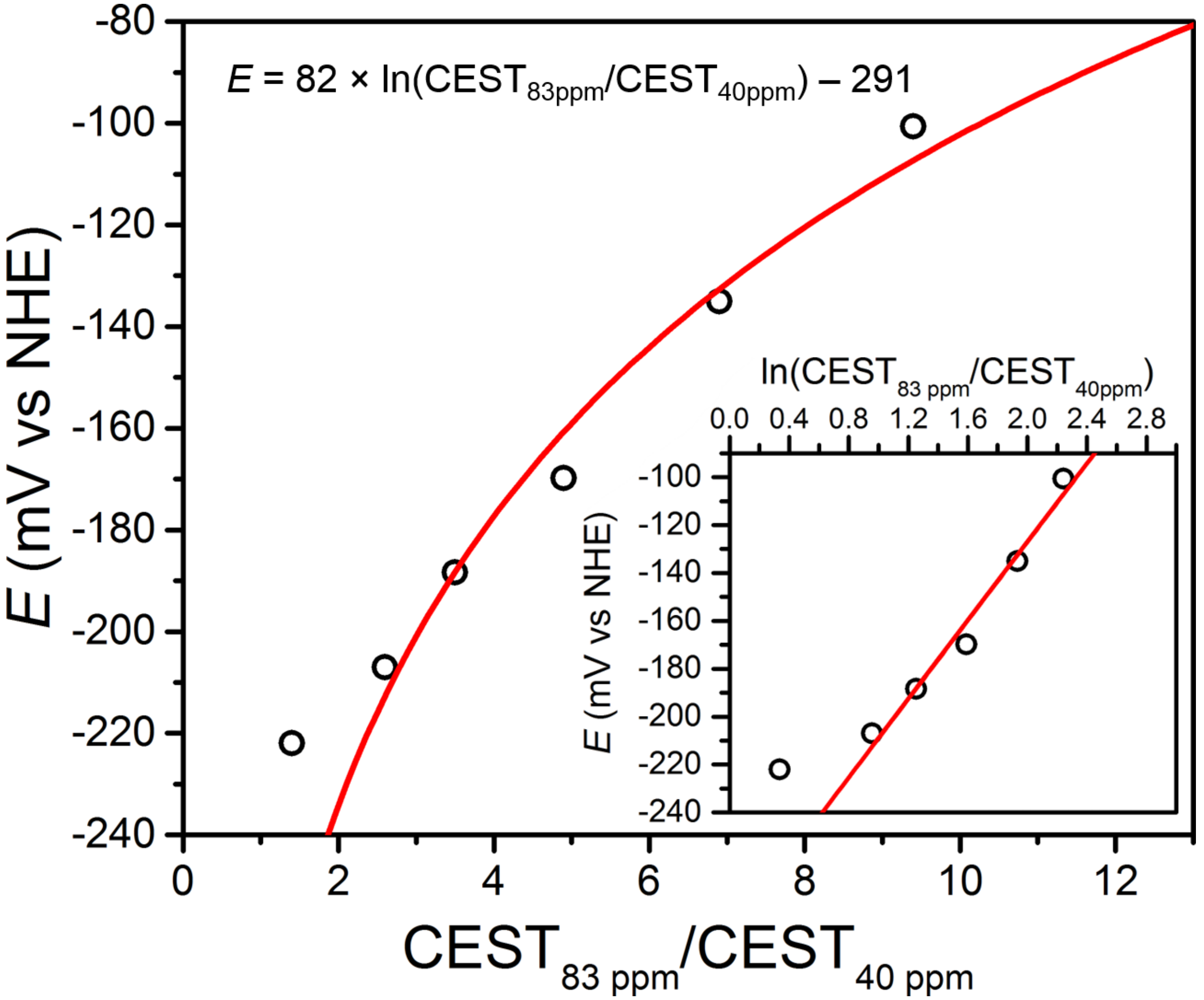

Figure S42. Open circuit potentials for solutions for phantom experiments, containing 100 mM of HEPES, 100 mM of NaCl and 10 mM of Fe_2 buffered at pH 7.4, is plotted against both the ratio of CEST effects at 37 °C from the averaged phantom image intensity with presaturation at 83 and 40 ppm and the natural log of the ratio (inset). Black circles and the red line represent the experimental data and the fit (equation displayed), respectively. Refer to Experimental Section for fitting details.

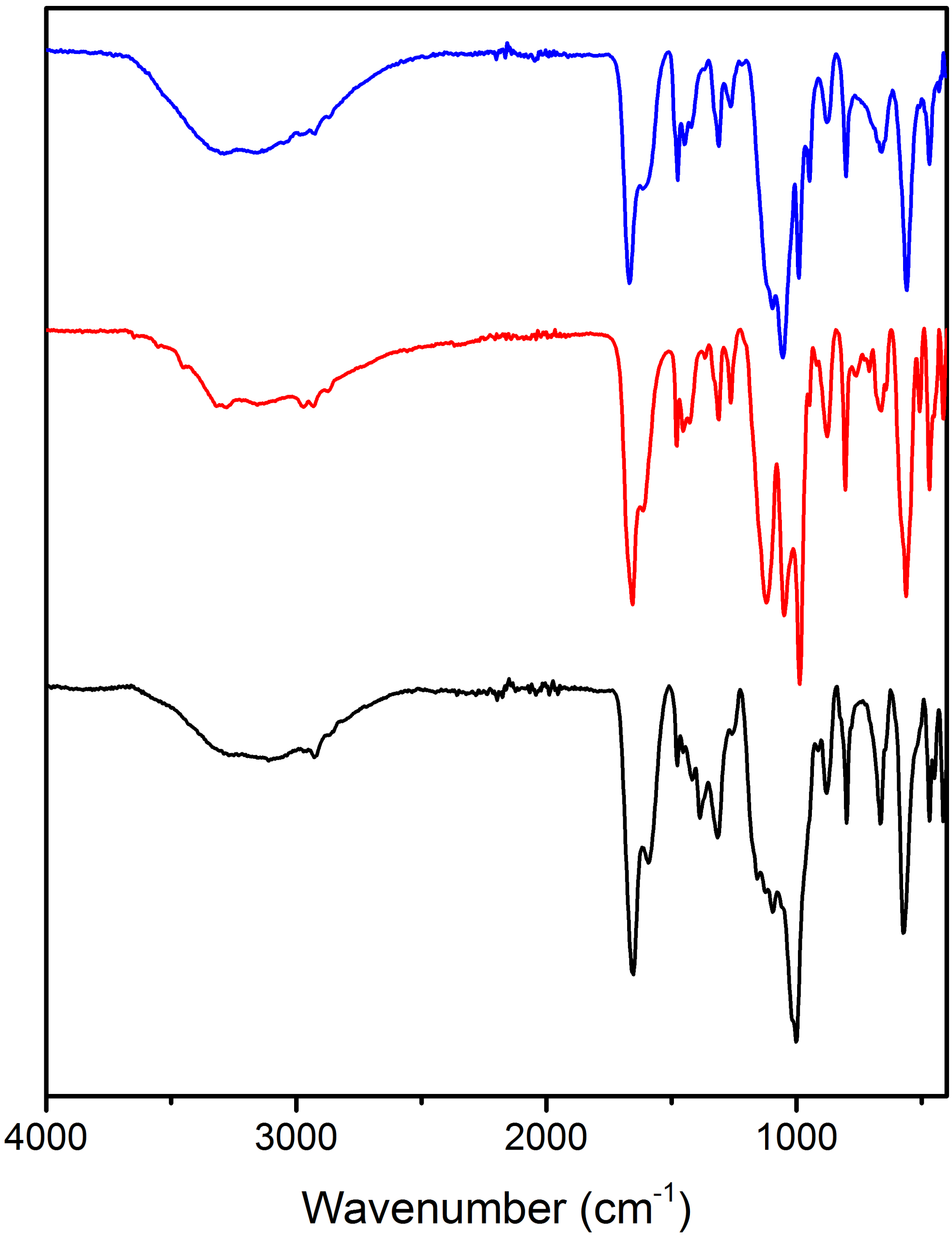

Figure S43. Stacked IR spectra of **1** (blue), **2** (red) and **3** (black).

References

(1) K. Du and T. D. Harris, *J. Am. Chem. Soc.* 2016, **138**, 7804
(2) APEX2, v. 2009 ; Bruker Analytical X-Ray Systems, Inc: Madison, WI, 2009.
(3) G. M. Sheldrick, SHELXTL, Version 6.12; Bruker Analytical X-ray Systems, Inc.: Madison, WI, 2000.
(4) G. M. Sheldrick, *Acta Crystallogr., Sect. A: Found. Adv.* 2015, **71**, 3.
(5) W. T. Dixon, J. Ren, A. J. M. Lubag, J. Ratnakar, E. Vinogradov, I. Hancu, R. E. Lenkinski, A. D. Sherry, *Magn. Reson. Med.* 2010, **63**, 625.
(6) G. A. Bain and J. F. Berry, *J. Chem. Ed.* 2008, **85**, 532.
(7) (a) Magnetic susceptibility data were simulated using the program MagProp: P. L. W. Tregenna-Piggott, *MagProp (part of the NIST DAVE software suite)*, version 2.0, 2008, http://www.ncnr.nist.gov/dave; (b) R. T. Azuah, L. R. Kneller, Y. M. Qiu, P. L. W. Tregenna-Piggott, C. M. Brown, J. R. D. Copley, R. M. Dimeoo, *J. Res. Natl. Inst. Stand. Technol.* 2009, **114**, 341.
(8) (a) D. F. Evans, *J. Chem. Soc.* 1959, 2003; (b) E. M. Schubert, *J. Chem. Educ.*, 1992, **69**, 61.
(9) D. T. Sawyer, A. J. Sobkowiak, J. Roberts, *Electrochemistry for Chemists*, 2nd ed.; John Wiley & Sons: New York, 1995
(10) (a) N. R. Kestner, J. Logan, J. Jortner, *J. Phys. Chem.* 1974, **78**, 2148; (b) J. J. Hopfield, *Proc. Natl. Acad. Sci. U. S. A.* 1974, **71**, 3640; (c) T. J. Meyer *Chem. Phys. Lett.* 1979, **64**, 417; (d) A. S. Borovik, V. Papaefthymiou, L. F. Taylor, O. P. Anderson, L. Que Jr, *J. Am. Chem. Soc.* 1989, **111**, 6183.
